# Supplementary material for: Past and Future Alcohol-Attributable Mortality in Europe
Source: Int J Environ Res Public Health. 2020 Dec 3;17(23):9024. doi: 10.3390/ijerph17239024 (PMC7730378; doi:10.3390/ijerph17239024)
Supplement: Supplementary file 1 [file ijerph-17-09024-s001.zip › Supplementary file 1. Additional information data and methods.pdf]

Past and future alcohol-attributable mortality in Europe - Additional information on data and methods

Estimation of alcohol-attributable mortality

We obtained estimated population-level alcohol-attributable mortality rates by sex, five-year age groups (20-24, …, 80-84), single calendar year (1990-2016) and country from the Global Burden of Disease (GBD) Study 2017 (Stanaway et al. 2018) for 30 European countries (= the countries in the final sample plus Bulgaria, Estonia, Latvia, and Slovakia) through the GBD results tool by IHME (IHME 2019). By applying an attributable fraction (AF) approach, the GBD 2017 study estimated for each country the deaths that can be attributed to alcohol by sex and age. In doing so, the GBD used three types of data: i) mortality data from various causes of death (partly) related to alcohol (see Appendix Box A1 in the main document); ii) alcohol consumption data, which they mainly obtained from a wide range of national representative health surveys; and iii) information on the health risks of alcohol consumption (cause-specific relative risks at different levels of drinking) stemming from a systematic literature review (Stanaway et al. 2018). The GBD estimates of alcohol-attributable mortality, consequently, include both the deaths from causes of death wholly related to alcohol, as well as an estimate of the alcohol-related deaths from causes of death partly related to alcohol (Stanaway et al. 2018). As such these estimates can better reflect the true alcohol-attributable mortality levels compared to estimates that only use cause-specific mortality data, and either include or exclude all deaths from causes that are only partly related to alcohol (e.g., external causes of death)(Trias-Llimos et al. 2018).

However, AF approaches require several assumptions, which are discussed extensively elsewhere (e.g. Trias-Llimós et al. 2018; Rey & Jougla 2014; Rehm et al. 2007; Rehm 2010). An important consequence is that the GBD estimates of alcohol-attributable mortality for the highest ages (65+) are considered implausible, as they are either very high or negative (e.g., Trias-Llimós et al. 2018; Manthey & Rehm 2019). Among the explanations for these potential inaccuracies are that the estimation technique is highly dependent on the limited information on alcohol use at these ages; age-specific RRs of dying are lacking at these ages; and, more generally evidence regarding the impact of alcohol on health at those ages is lacking (e.g., Trias-Llimós et al. 2018; Manthey & Rehm 2019). That is, most RRs that are used as part of the GBD are based on studies that focused on adult populations (e.g. Corrao et al. 2004), whereas the little evidence that exists on mortality risks by age group suggests that the relative risks are not necessarily identical for the older population compared to the adult population (e.g. Roerecke & Rehm 2012). In addition, the old age population is often not well sampled in health surveys and therefore lifetime and current alcohol consumption patterns and estimates may be less accurate for older than for adult populations. This limited information on alcohol use at older ages represents an additional difficulty in estimating alcohol-related mortality (as suggested in Trias-Llimós et al. 2018).
Therefore, we adjusted the GBD estimates of alcohol-attributable mortality for 65+ by applying to them the age pattern for the highest ages (only their shape, not their level) observed for the main group of causes of death wholly attributable to alcohol, which is regarded as more realistic (Trias-Llimós et al. 2018). For this purpose, we used cause-specific mortality data from the WHO Mortality Database (WHO 2018) for the years included in ICD-10. We obtained these data for the following wholly alcohol-related causes of death: 'mental and behavioural disorders due to use of alcohol', 'alcoholic liver disease', 'accidental poisoning by and exposure to alcohol', 'degeneration of nervous system due to alcohol',

‘alcoholic polyneuropathy’, ‘alcoholic gastritis’, ‘alcohol-induced chronic pancreatitis’, ‘foetal alcohol syndrome’, ‘intentional self-poisoning and exposure to alcohol’ and ‘poisoning by and exposure to alcohol’ (ICD-10 codes: F10, K70, X45, G312, G621, G721, I426, K292, K860, Q860, X65, and Y15), as identified by Semyonova et al. 2014.

Subsequently, we calculated ratios between the alcohol-attributable mortality rates based on the WHO data for the five-year age groups from ages 65-69 onwards and the respective rates at ages 60-64 for each country and sex, for all years combined (see Figure S1). We subsequently applied these ratios (which represent the age pattern at the highest ages) to the GBD alcohol-attributable mortality rate at ages 60-64 to obtain the adjusted alcohol-attributable mortality rates for ages 65-69 and older. For example, if for a given population alcohol-related mortality based on causes of death wholly related to alcohol was 20% lower at ages 70-74 than at ages 60-64, we multiplied the GBD alcohol-attributable mortality rates at ages 60-64 by 0.8 to obtain the adjusted alcohol-attributable mortality rate at ages 70-74. For country-years with data for age groups up to 85+ (France, United Kingdom), we applied the ratio for ages 85+ to the age groups 85-89, 90-94, and 95+.

Figure S1 – Sex- and age-specific ratios used to adjust the age patterns for ages 65+ in the GBD data, based on alcohol-attributable cause-specific mortality data (WHO; Human Cause of Death Database)

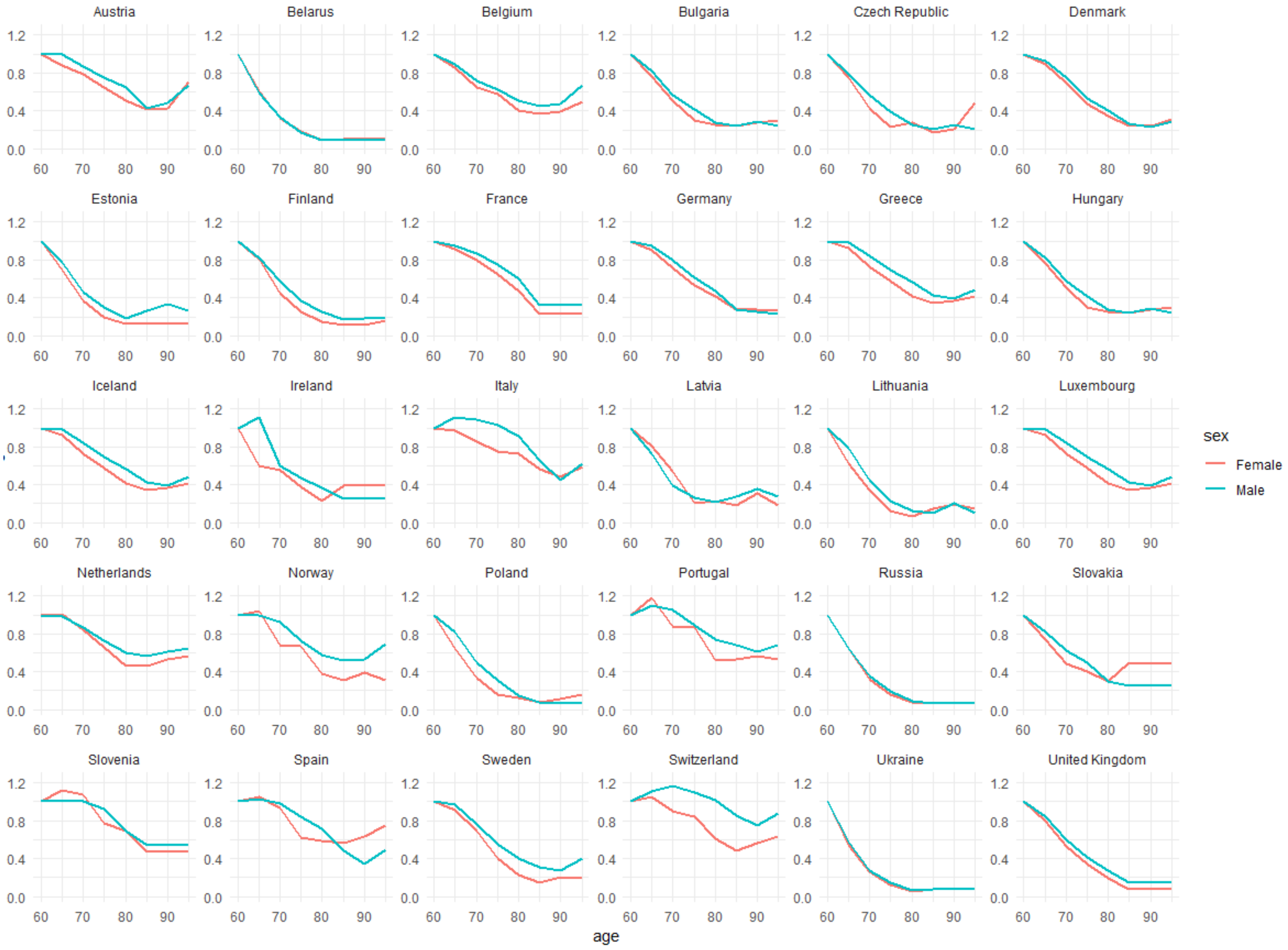

For countries without ICD-10 data in the WHO Mortality Database (Belarus, Hungary, Russia, Ukraine) or with insufficient data from WHO (Iceland, Bulgaria, Greece, Luxembourg), we calculated and applied alternative ratios. That is, for Belarus, Russia and Ukraine we calculated and applied the ratios using the alcohol-related mortality data (ICD-10 codes: F10, K70, X45) that is available from the Human Cause of Death Database (2017). For the remaining countries not included in the HCDD, we used the average WHO weights over the western European countries in our analysis for Greece, Luxembourg and Iceland, and the average WHO weights over the Eastern European countries in our analysis for Bulgaria and Hungary.

Even after this adjustment we ended up with negative age-specific alcohol-attributable mortality rates at the (very) old ages, particularly in Estonia, Latvia and Slovakia, which we did not consider likely, in line with the literature disputing the (cardio)protective effects of alcohol on mortality (e.g. Holmes et al. 2014). Consequently, we excluded Estonia, Latvia, and Slovakia from our analysis, and we conducted our final analysis on the ages 20-84.

See the Appendix of this document for a comparison of our age-specific and age-standardised alcohol-attributable mortality estimates with the GBD estimates.

The resulting age-specific alcohol-attributable mortality rates (20-24, 25-29, …, 80-84) by sex, country, and year (1990-2016) were divided by the respective all-cause mortality rates from the Human Mortality Database (2018) in order to obtain the alcohol-attributable mortality fractions by five-year age groups. Because for Bulgaria, data from the Human Mortality Database were only available up until 2010, we decided to exclude Bulgaria from our analysis as well.

To obtain estimates of alcohol-attributable mortality fractions by single year of age, we applied Loess smoothing (span = 0.5; degree = 2) to the log-transformed fractions by five-year age groups, after carefully considering other smoothing approaches.

To obtain an estimate of alcohol-attributable mortality fractions across the adult ages ($AAMF_{s,t}$) that could be compared over time (both over the past and into the future), we applied direct age standardisation. We standardised the smoothed $AAMF_{x,s,t}$ using the population-specific age distribution of deaths in 2010. The latter information was also obtained from the Human Mortality Database (2018).

Details behind the projection methodology

Age-period-cohort modelling

To project age-specific alcohol-attributable mortality fractions up to 2060, we employed an advanced age-period-cohort projection methodology. As the basis, we utilized the age-period-cohort modelling approach by Clayton and Schifflers (1987). This approach deals with the linear dependency between period and birth cohort (age = period – cohort) by decomposing mortality into the shared linear trend between period and cohort (= drift), a non-linear period effect, and a non-linear cohort effect. To simplify the interpretation and the projection, we clubbed the drift with the non-linear period effect using the Cairns et al. (2009) approach, which is implemented in the Stochastic Mortality Modelling (StMoMo)

package (Villegas et al. 2015) in R. More specifically, this comprised the application of a set of constraints to – in our case – the cohort parameter. Thus, our period parameter captures the entire linear time trend (= includes the drift), while the cohort parameter captures the cohort variations from this overall trend. This approach results in a period parameter that is largely in line with the age-standardised AAMF, and a cohort parameter that is still relatively easy to interpret.

In applying the age-period-cohort model to the age-specific alcohol-attributable mortality fractions (AAMF), we used a generalised logit as the link function. The logit transformation ensured future AAMFs between zero and one, and and enabled us to project (eventually) declining AAMF for selected countries with currently increasing AAMF, in line with our general projection approach. In addition, we generalised the APC model to include more restricted lower bounds of the projected fractions and their projection intervals (PIs), in order to avoid unrealistic crossovers between men and women and between countries.

The final model we applied for each country, by sex, is:

$$logit\left(\frac{AAMF_{x,t} - LB_x}{UB_x - LB_x}\right) = \tilde{\alpha}_x + \tilde{\kappa}_t + \tilde{\gamma}_{t-x} .$$

where $AAMF_{x,t}$ are smoothed alcohol attributable mortality fractions by single years of age (x) and year (t). LB_x stands for the age-specific lower bounds, which are constant over time but differ by population (see below). UB_x stands for the age-specific upper bounds, which we set to one for all populations and time periods. The transformed parameters $\tilde{\alpha}_x$, $\tilde{\kappa}_t$, and $\tilde{\gamma}_{t-x}$ capture the age pattern, the overall time trend (period), and the cohort-specific deviations from the time trend, respectively.

Age-specific lower bounds and age-standardised lower limits

We imposed age-specific lower bounds for each population on the basis of assumed lower limits to age-standardised AAMF. That is, we assumed that in the future, the age-standardised AAMF would remain higher for men than for women, whom historically always exhibited lower AAMF levels. Similarly, based on past observations, we consider it unlikely that among men, the higher current age-standardised AAMF values in Eastern European countries would become lower in the future than those in Western European countries. For this reason, we selected different lower limits of age-standardised AAMF for different groups of countries, and obtained the age-specific lower bounds by applying to these lower limits the population-specific age pattern observed in 2016/LAY.

More specifically, for the selection of the lower limit of age-standardised AAMF, we categorised the countries according to their past trends and their past (peak) levels of age-standardised AAMF. In selecting the actual lower limit per category of countries, we also had to keep in mind that the implementation of the resulting age-specific lower bounds can lead to the omission of past age-specific values when these past values are lower than the lower bound.

The past trends in age-standardised alcohol-attributable mortality fractions (AAMF) (Figure 1) clearly show that for men in selected non-Eastern European countries, the decline was stagnating at levels between approximately 5% and 10%. We do not expect that among men in Eastern European countries, AAMF levels will be lower than these stagnating levels. Therefore, we imposed a lower age-standardised AAMF limit of 5% in Eastern European countries with a high peak (Czech Republic, Hungary, Lithuania,

Poland, and Slovenia). For Belarus, Russia, and Ukraine, where very high peak levels are observed, we imposed a lower limit of 7.5%.

For men in the countries with (decelerating) declines (France, Portugal, Germany, Switzerland, Austria, Greece, Spain, Italy), we selected a largely symbolic lower limit of 1.5% (three times as high as the lower bound for women)(see below). In practice, future AAMF levels (up to 2060) are projected to fall below 5% for Switzerland only (4.9%).

For men in the remaining non-Eastern European countries with either an increase followed by a decline, or a (decelerating) increase, a clear distinction can be made between countries with an (expected) low peak (Norway, UK, Sweden, Iceland), and countries with an (expected) high peak (Belgium, Denmark, Finland, Luxembourg, Ireland); with the Netherlands in between. For the first group, we selected a lower bound of one (although for Iceland, we had to adjust the lower limit slightly downwards to avoid omitting too many past observations). This lower limit was chosen because a level below 1.5% was already observed among men in Iceland in 1990. For men in the countries with an (expected) high peak, we selected a lower limit of 2%, because the observed past peaks in these countries were generally twice as high as those in the countries with observed low peaks. For men in the Netherlands, we selected a lower limit of 1.5%.

Table S1 Categorisation of countries and the selected lower limits of the age-standardised alcohol-attributable mortality fractions (20-84)

	Men			Women	
**Country**	**Categorization past trend**	**Lower limit**		**Categorization past trend**	**Lower limit**
Austria	(decelarating) decline	1.50%		(decelarating) decline	1.00%
France	(decelarating) decline	1.50%		(decelarating) decline	1.00%
Germany	(decelarating) decline	1.50%		(decelarating) decline	1.00%
Greece	(decelarating) decline	1.50%		(decelarating) decline	0.00%
Italy	(decelarating) decline	1.50%		(decelarating) decline	1.00%
Portugal	(decelarating) decline	1.50%		(decelarating) decline	1.00%
Spain	(decelarating) decline	1.50%		(decelarating) decline	1.00%
Switzerland	(decelarating) decline	1.50%		(decelarating) decline	1.00%
Iceland	Low Peak	0.13%		Low Peak	0.08%
Norway	Low Peak	1.00%		Low Peak	0.33%
Sweden	Low Peak	1.00%		Average Peak	0.66%
United Kingdom	Low Peak	1.00%		Low Peak	0.33%
Netherlands	Middle Peak	1.50%		Average Peak	0.66%
Belgium	Average Peak	2.00%		Average Peak	0.66%
Denmark	Average Peak	2.00%		High Peak (West)	1.25%
Finland	Average Peak	2.00%		Average Peak	0.66%
Ireland	Average Peak	2.00%		Average Peak	0.66%
Luxembourg	Average Peak	2.00%		High Peak (West)	1.25%
Czech Republic	High Peak	5.00%		Average Peak	0.66%
Hungary	High Peak	5.00%		High peak (East)	2.00%
Lithuania	High Peak	5.00%		Average Peak	0.50%
Poland	High Peak	5.00%		Low Peak	0.33%
Slovenia	High (past) peak	5.00%		(decelarating) decline	1.00%
Belarus	Very High Peak	7.50%		High Peak (East)	2.00%
Russia	Very High Peak	7.50%		High Peak (East)	2.00%
Ukraine	Very High Peak	7.50%		High Peak (East)	2.00%

For women, stagnation of AAMF was clearly evident only in Greece, at a level of 1%. Therefore, for women in the countries that currently display (decelerating) declines (France, Portugal, Germany, Switzerland, Spain, Italy, Austria), we set a lower limit of 1%. For Greece, implementing lower bounds proved unnecessary.
The differences in AAMF levels between Eastern and Western Europe were much smaller for women than for men, with crossovers already clearly visible. Therefore, for women in the countries with a trend in age-standardised AAMF that is generally increasing, we selected the lower limits that were more in line with the observed differences in the (expected) peak in AAMF. Particularly for women in Western Europe, we took into account the lowest AAMF levels already observed (in Iceland, at < 1%), and the current differences between men and women in the AAMF levels.
For women in Iceland, who exhibit a very low peak, we set the lower limit at 0.075%, which represents half the value of the lower bound set for men in Iceland. For women in countries with an (expected) low peak (Norway, United Kingdom, Poland) we set the lower limit at 0.33%. For Norway and the UK, this level represents one-third of the value of the lower limit selected for men in these countries. For women in countries with (expected) average peak values (Czech Republic, Belgium, Lithuania, the Netherlands, Sweden, Finland, and Ireland) we set a lower limit of 0.66%. For the non-Eastern European countries, this limit is between 1.5 and three times lower than the lower limit for men in the respective countries. For Lithuania, implementing this lower limit proved problematic; therefore, we reduced the lower limit to 0.5%. For women in Western European countries with very high (expected) recent peak values (Denmark, Luxembourg), we set a lower limit of 1.25%, which is 1.6 times lower than the lower limit set for men in these countries, and is two times higher than the lower limit set for the Western European countries with average peak values (which is approximately in line with the differences in observed peak values). For women in Eastern European countries with very high (expected) recent peak values (Belarus, Hungary, Russia, Ukraine), we set the lower limit slightly higher, at 2.0%, to ensure that the differences between men and women in these countries do not become too large in the future.

See Table S1 for the categorisation of the countries according to their past trends in age-standardised AAMF(20-84) and the lower limits we selected by sex and country group.

Age-specific lower bounds were obtained by applying the population-specific age pattern (ages 20-84) observed in the LAY to these lower limits. More specifically, we linearly transformed the population-specific age pattern observed in the LAY so that it would equal the value of the selected population-specific lower limit of the age-standardised AAMF. We did so by dividing, for each age, the age-specific AAMF by the ratio of the actual AAMF20-84 to the desired lower limit of AAMF20-84.

See Figure S2 for the age-specific lower bounds we implemented.

Figure S2. Age-specific lower bounds we implemented, by sex

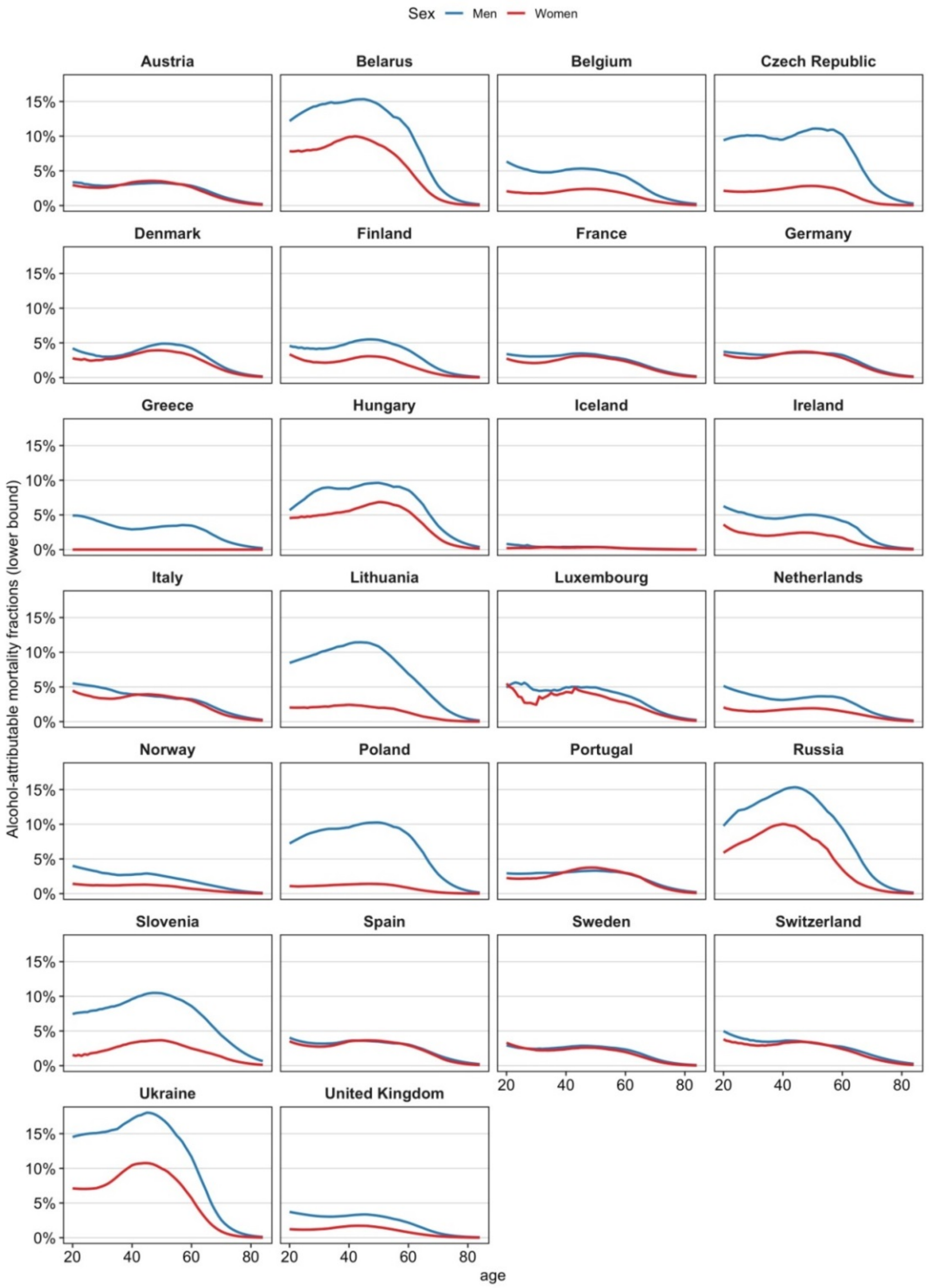

Projection of the parameters

For the projection of the period (k_t) and cohort (g_c) parameters, which we obtained from the application of our APC model to our data, we employed different strategies (see Box S1) for countries with different past trends in k_t and g_c (see Figure S3-S4).

We projected the (recent) trend in the period and cohort parameters mainly using stochastic time-series forecasting (ARIMA). ARIMA(p,d,q) models are a very general class of time-series models for forecasting future values based on past observed values, in which *p* denotes the order of the auto-regressive model (= how many previous time points of the time-series to use in the auto-regression), *d* is the degree of differencing required to obtain a stationary time-series, and q is the order of the moving-average model (= the lag of the error component) (Box et al. 2015). We selected the best-fitting ARIMA model subject to some constraints, based on minimum *AICc* (Akaike Information Criterion), using the forecast package in R (Hyndman et al. 2019).

However, for populations with a trend in age-standardised AAMF that is generally increasing, we extrapolated the period parameter deterministically by means of a quadratic curve. That is, a quadratic curve in the logit of fractions will result in a wave pattern in the normal fractions. Consequently, the resulting projections are in line with the idea of a wave-shaped epidemic, as observed for alcohol in other countries and for smoking in all European countries.

Box S1 - Strategy for the projection of the period and cohort parameters based on their past trends

**Past trend**	**Projection strategy**
**Period parameter (k_t)**	
Continued decline (N = 6)	Projection decline by best ARIMA (p<=3, d=1, q<=3) with drift for whole period
Deceleration of decline (N = 8)	Projection recent decline by best ARIMA (p<=3, d=1, q<=3) with drift for year since trend break
Decline with recent stagnation (N = 6)	Projection recent trend by best ARIMA (p<=3, d=1, q<=3) with drift. If recent increase, project stable level by ARIMA (0,1,0) without drift.
Increase with recent decline (N = 26)	Quadratic over whole period
Increase without recent decline (N = 6)	Quadratic over period that shows the best fit
**Cohort paramater (g_c)**	
Reversed U-shape without recent stagnation (N = 17)	Recent downward trend extrapolation by best ARIMA (p<=3,d=1,q<=3) model with drift
Reversed U-shape with recent stagnation (N = 17)	Recent trend extrapolation (best ARIMA (p<=3, d, q <=3)); when increase => stable trend by ARIMA (0,1,0) with no drift
Recent decline (N = 5)	Recent downward trend extrapolation by best ARIMA (p<=3,d=1,q<=3) model with drift
Fluctuating trend (N = 10)	Mean reverting process around zero by best ARIMA (p<=3,0,q<=2) with zero mean on whole trend
U-shaped (N = 3)	ARIMA (0,1,0) without drift on whole trend

In performing the projections for k_t and g_c, we made sure we were not selecting two very different ARIMA models, in cases in which the past trends in one country looked rather similar for men and women. Moreover, we made sure that there was no resulting long-term divergence between the fractions for men and women.

To ensure more robust estimates and to diminish the projection intervals, we applied the projections to the longest observation window possible: i.e., either the whole time-series if there was no change in the trend, or the trend from a certain trend break.

Figure S3 Past trend period parameter (k_t) for the different countries, according to group

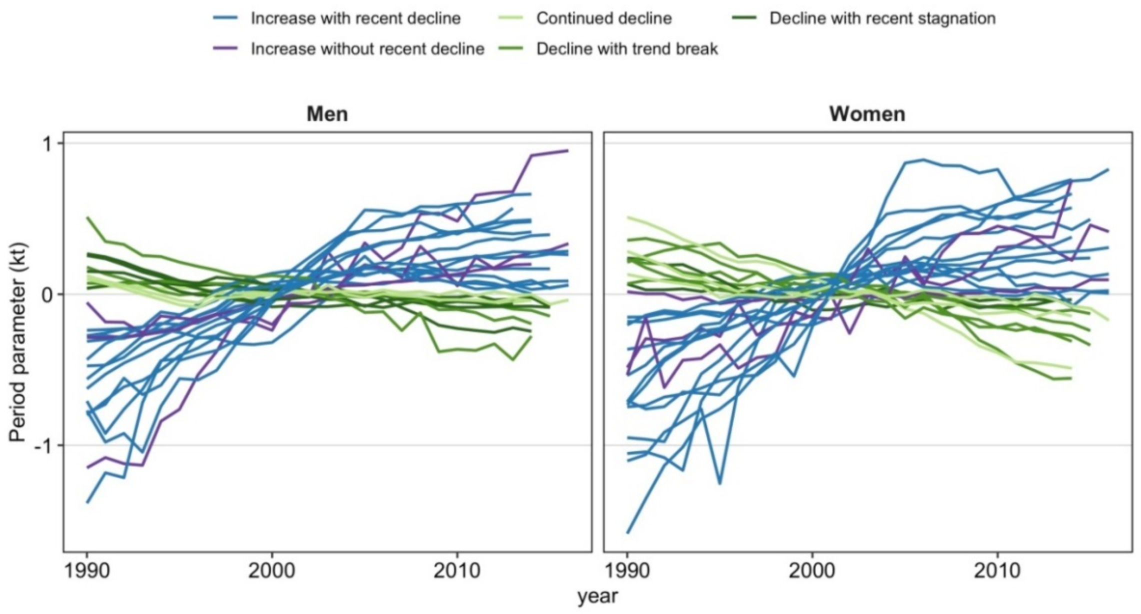

Figure S4 Past trend cohort parameter (g_c) for the different countries compared, according to group

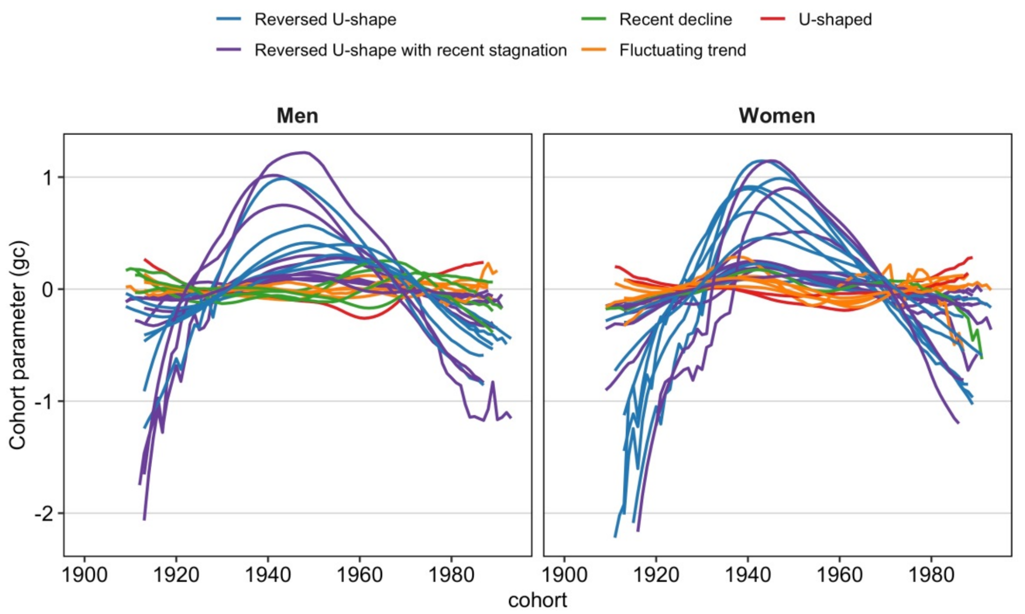

Projection of the period parameter

The period parameter (k_t) is projected into the future by a quadratic curve with correlated errors for populations with predominantly increasing k_t trends, and, for populations in which these trends are mainly declining, by extrapolation of the decline by the best-fitting ARIMA (Auto Regressive Integrated Moving Average) model (p<=3,d=1,q<=3) with drift, based on minimum AICc (Akaike Information Criterion). For populations in which the decline in k_t is followed by a recent increase, we implemented a stable future k_t trend using an ARIMA(0,1,0) without drift.

More specifically, we divided the k_t trends into five categories (see Figure S3) and devised for each of these categories a different projection model to optimally extrapolate the past trend observed.

1) **Continued decline.** For countries with a decline and without a clear trend break in k_t, use the best ARIMA (p, d=1, q) model (according to AICc) for the whole period from the start of the decline, thereby enforcing a drift, and maximum p and q = 3.
2) **Decline with trend break.** For countries with a decline that is not constant, use the best-fitting ARIMA (p, d=1, q) model (according to AICc) for the period that best depicts the recent decline, thereby enforcing a drift, and maximum p and q = 3.
3) **Decline with recent stagnation.** For those countries with a decline with a recent stagnation (deceleration / level / recent increase), project the trend from the break with the best ARIMA (p,1,q) model with drift (according to AICc). If there is a recent increase, enforce a stable trend by applying ARIMA (0,1,0) with no drift over the period with the recent increase.
4) **Increase with recent decline.** For populations with increasing trends in k_t, but with a recent trend break, a quadratic model is used from the start of the increase (mostly the whole period).
5) **Increase without recent decline**. For populations with increasing trends in k_t, but without a recent trend break, a bell-shaped quadratic model is used over the period that best fits the data.

See Table S2 for the specifics of the period projection by country and sex.

Table S2 – Specifics period projection

Population	Trend description kt	Projection principle kt	First year	Last year	Modelling of errors for the quadratic projections / Final kt model for the remaining projections
Belarus_Female	Increasing with decelerating increase	quadratic	1990	2016	Error model: ARIMA(1,0,0) with zero mean
Belarus_Male	Increasing from 1991 with recent stagnation	quadratic	1991	2016	Error model: ARIMA(1,0,0) with zero mean
Belgium_Female	Increasing from 2000 with decelerating increase	quadratic	2000	2015	Error model: ARIMA(1,0,0) with non-zero mean
Belgium_Male	Increasing from 2000 with decelerating increase	quadratic	2000	2015	Error model: ARIMA(0,0,1) with zero mean
Czech Republic_Female	Increasing with recent decline	quadratic	1990	2016	Error model: ARIMA(2,0,1) with zero mean
Czech Republic_Male	Increasing from 1995 with recent decline	quadratic	1995	2016	Error model: ARIMA(1,0,0) with zero mean
Denmark_Female	Increasing with recent decline	quadratic	1990	2016	Error model: ARIMA(1,0,0) with zero mean
Denmark_Male	Increasing with recent decline	quadratic	1990	2016	Error model: ARIMA(1,0,0) with zero mean
Finland_Female	Increasing from 1992 with stagnation	quadratic	1992	2015	Error model: ARIMA(1,0,0) with zero mean
Finland_Male	Increasing with stagnation	quadratic	1990	2015	Error model: ARIMA(1,0,0) with zero mean
Ireland_Female	Increasing with recent decline	quadratic	1990	2014	Error model: ARIMA(1,0,0) with non-zero mean
Ireland_Male	Increasing with recent decline	quadratic	1990	2014	Error model: ARIMA(1,0,0) with non-zero mean
Lithuania_Female	Increasing from 1992; change increase 1998; recent stagnation	quadratic	1999	2014	Error model: ARIMA(1,0,0) with zero mean
Lithuania_Male	Increasing from 1993; change increase 1997; decelerating increase	quadratic	1997	2014	Error model: ARIMA(1,0,0) with zero mean
Norway_Female	Increasing with decelerating increase	quadratic	1990	2014	Error model: ARIMA(1,0,0) with zero mean
Norway_Male	Increasing from 1991 with decelerating increase	quadratic	1991	2014	Error model: ARIMA(1,0,0) with non-zero mean
Poland_Female	Increasing from 1993 with decelerating increase	quadratic	1993	2014	Error model: ARIMA(0,0,2) with zero mean
Poland_Male	Increasing with decelerating increase	quadratic	1990	2014	Error model: ARIMA(1,0,0) with zero mean
Russia_Female	Increasing with recent decline	quadratic	1990	2014	Error model: ARIMA(2,0,0) with zero mean
Russia_Male	Increasing with recent decline	quadratic	1990	2014	Error model: ARIMA(0,0,1) with zero mean
Sweden_Female	Increasing with recent decline	quadratic	1990	2016	Error model: ARIMA(0,0,1) with zero mean
Sweden_Male	Increasing with recent decline	quadratic	1990	2016	Error model: ARIMA(1,0,0) with zero mean
Ukraine_Female	Increasing; change increase 1999; recent stagnation	quadratic	1999	2013	Error model: ARIMA(2,0,0) with zero mean
Ukraine_Male	Increasing; change increase 1998; recent decelerating increase	quadratic	1998	2013	Error model: ARIMA(2,0,0) with zero mean
United Kingdom_Female	Increasing with decelerating increase	quadratic	1990	2016	Error model: ARIMA(2,0,0) with zero mean
United Kingdom_Male	Increasing with decelerating increase	quadratic	1990	2016	Error model: ARIMA(2,0,0) with zero mean
Iceland_Female	Continued increase	quadratic enforced	2002	2014	Error model: ARIMA(1,0,0) with non-zero mean
Iceland_Male	Continued increase	quadratic enforced	1990	2016	Error model: ARIMA(1,0,0) with non-zero mean
Luxembourg_Female	Continued increase from 1992	quadratic enforced	1999	2013	Error model: ARIMA(1,0,0) with non-zero mean
Luxembourg_Male	Continued increase from 2000	quadratic enforced	2000	2014	Error model: ARIMA(1,0,0) with non-zero mean
Netherlands_Female	Continued increase from 1995	quadratic enforced	1995	2011	Error model: ARIMA(1,0,0) with zero mean
Netherlands_Male	Continued increase	quadratic enforced	1995	2015	Error model: ARIMA(2,0,0) with zero mean
Austria_Male	Decreasing without trend break	decline	1990	2014	Kt model: ARIMA(0,1,0) with drift
Italy_Female	Decreasing without trend break	decline	1990	2014	Kt model: ARIMA(0,1,0) with drift
Spain_Female	Decreasing without trend break	decline	1990	2014	Kt model: ARIMA(0,1,0) with drift
Spain_Male	Decreasing without trend break	decline	1990	2014	Kt model: ARIMA(0,1,0) with drift
Switzerland_Female	Decreasing without trend break	decline	1990	2016	Kt model: ARIMA(0,1,0) with drift
Switzerland_Male	Decreasing without trend break	decline	1990	2016	Kt model: ARIMA(0,1,0) with drift
France_Female	Decreasing with trend break in 1998, still decline	recent decline	1998	2015	Kt model: ARIMA(0,1,0) with drift
France_Male	Decreasing with trend break in 1998, still decline	recent decline	1998	2015	Kt model: ARIMA(0,1,0) with drift
Germany_Female	Decreasing with trend break in 1993, still decline	recent decline	1993	2015	Kt model: ARIMA(0,1,0) with drift
Hungary_Female	Decreasing with trend break in 2004, still decline	recent decline	2004	2014	Kt model: ARIMA(0,1,0) with drift
Hungary_Male	Decreasing with trend break in 2006, still decline	recent decline	2006	2014	Kt model: ARIMA(0,1,0) with drift
Portugal_Female	Decreasing with trend break in 1999 and 2001, still decline	recent decline	2001	2015	Kt model: ARIMA(1,1,0) with drift
Slovenia_Female	Decreasing with trend break in 2001, still decline	recent decline	2001	2014	Kt model: ARIMA(1,1,0) with drift
Slovenia_Male	Decreasing with trend break at 2002, still decline	recent decline	2002	2014	Kt model: ARIMA(0,1,0) with drift
Austria_Female	Decreasing with trend break at 1999, no more decline	recent trend	1999	2014	Kt model: ARIMA(0,1,0)
Germany_Male	Decreasing with trend break in 2010, no more decline	recent trend	2010	2015	Kt model: ARIMA(0,1,0)
Greece_Female	Decreasing with trend break in 2000, no more decline	recent trend	2000	2013	Kt model: ARIMA(0,1,0)
Greece_Male	Decreasing with trend break in 2000, no more decline	recent trend	2000	2013	Kt model: ARIMA(0,1,0)
Italy_Male	Decreasing with trend break in 2009, deceleration	recent trend	2009	2014	Kt model: ARIMA(0,1,0) with drift
Portugal_Male	Decreasing with trend break in 1995, deceleration	recent trend	1995	2015	Kt model: ARIMA(0,1,0) with drift

Projection of the cohort parameter

We based the projection of the gamma parameter (g_c) on the trend after omitting (=burning) the outer cohorts to ensure stable trends. To decide how many cohorts to omit/burn we performed a statistical test. That is, by employing a t-test to the cohort parameter, we assessed which cohorts did not differ from zero at a statistical significance level (p) of 0.05. In principle, we burned the first and last five cohorts. However, if the statistical test (i.e., burn cohorts with $p > 0.05$) indicated that only the last three cohorts were not statistically significant from zero, we burned the first and last three cohorts. If the statistical test indicated that seven or more of the last cohorts were not statistically significant from zero, we burned the first and last seven cohorts. For women in Iceland, we burned the six last cohorts, and for men in Greece, we burned the last seven cohorts; thereby deviating from the outcomes of the statistical procedure.
See Table S3 for the N of the outer cohorts that we omitted (=burned) in the end.

Following a close inspection of the trends in g_c after burning the cohorts, we assigned the countries to five different groups (see Figure S4). For each group of countries, we employ a different main strategy. However, these strategies all boil down to the same approach: i.e., the recent trend is extrapolated as much as possible by means of the best-fitting (constrained) ARIMA model. When this leads to an increase, we enforced a stable trend using ARIMA(0,1,0) with no drift.

1. **Reversed U-shape (=bell shaped).** For those populations in which the cohort parameter showed a reversed U-shape without a recent stagnation, we extrapolated the recent downward trend of g_c from a potential trend break by applying the best-fitting ARIMA (p<=3,d=1,q<= 3) model with drift.
2. **Reversed U-shape (= bell shaped) with recent stagnation.** For those populations in which the cohort parameter showed a reversed U-shape with a recent stagnation of the downward trend, we use a recent trend extrapolation of g_c applied to either the last 10 years (when stagnation <= five years before the first burned cohort) or the period since the stagnation (when stagnation > five years before the first burned cohort). We do so by applying the best-fitting ARIMA (p<=3, d, q <=3) model (no enforcement of drift). However, when this leads to an increase, we enforced a stable trend by using ARIMA (0,1,0) with no drift over the respective period.
3. **Recent decline.** For those populations in which the cohort parameter revealed a recent decline, we extrapolated the recent downward trend from a potential trend break by applying the best-fitting ARIMA (p<=3,d=1,q<= 3) model with drift.
4. **Fluctuating trend.** For those populations in which the cohort parameter fluctuates around zero, we extrapolated the fluctuating trend by a mean-reverting process around zero by applying the best-fitting ARIMA(p<=3, 0, q<=2) model with zero mean on the whole trend (after burning).
5. **U-shaped.** For those populations in which the cohort parameter revealed a U-shaped pattern, we projected by means of an ARIMA (0,1,0) without drift on the entire cohort trend (after burning).

We used the longest possible cohort trend in order to produce relatively small prediction intervals.

See Table S3 for the specifics of the cohort projection by country and sex.

Table S3 – Specifics cohort projection - organised according to g_c trend

Population	N burned outer cohorts	gc trend	gc principle	gc first year	gc last year	Final gc model
Austria_Female	7	bell shaped	Recent trend	1972	1987	ARIMA(0,1,0) with drift
Belarus_Female	7	bell shaped	Recent trend	1950	1989	ARIMA(1,1,0) with drift
Czech Republic_Female	7	bell shaped	Recent trend	1962	1989	ARIMA(3,1,0) with drift
Finland_Female	7	bell shaped	Recent trend	1943	1988	ARIMA(1,1,2) with drift
Finland_Male	3	bell shaped	Recent trend	1958	1992	ARIMA(2,1,2) with drift
Lithuania_Female	7	bell shaped	Recent trend	1949	1987	ARIMA(1,1,1) with drift
Netherlands_Female	3	bell shaped, deceleration from 1961	Recent trend	1961	1993	ARIMA(1,1,1) with drift
Netherlands_Male	3	bell shaped	Recent trend	1962	1993	ARIMA(2,1,0) with drift
Norway_Female	7	bell shaped	Recent trend	1946	1987	ARIMA(0,1,0) with drift
Norway_Male	7	bell shaped	Recent trend	1947	1987	ARIMA(1,1,0) with drift
Poland_Female	5	bell shaped	Recent trend	1945	1989	ARIMA(1,1,0) with drift
Sweden_Male	7	bell shaped	Recent trend	1953	1989	ARIMA(3,1,0) with drift
United Kingdom_Female	5	bell shaped	Recent trend	1948	1991	ARIMA(1,1,1) with drift
United Kingdom_Male	7	bell shaped	Recent trend	1961	1989	ARIMA(1,1,0) with drift
Denmark_Female	3	bell shaped	Recent trend	1947	1993	ARIMA(0,1,0) with drift
Denmark_Male	7	bell shaped	Recent trend	1960	1989	ARIMA(2,1,0) with drift
Lithuania_Male	7	bell shaped	Recent trend	1943	1987	ARIMA(1,1,3) with drift
Russia_Male	7	bell shaped; level/increase from 1986	Recent trend	1978	1987	ARIMA(0,2,0)
Iceland_Female	6	bell shaped; increase from 1988	Recent trend	1981	1990	ARIMA(0,1,0)
Russia_Female	7	bell shaped: level/increase from 1987	Recent trend	1978	1987	ARIMA(0,1,0)
Sweden_Female	7	bell shaped; level from 1987	Recent trend	1980	1989	ARIMA(0,1,0)
Ukraine_Female	7	bell shaped; deceleration from 1986	Recent trend	1977	1986	ARIMA(0,2,0)
Ukraine_Male	7	bell shaped; level from 1986	Recent trend	1977	1986	ARIMA(0,2,0)
Austria_Male	3	bell shaped; level/increasing from 1983	Recent trend	1983	1991	ARIMA(0,1,0)
Belarus_Male	7	bell shaped; increase from 1980	Recent trend	1980	1989	ARIMA(0,1,0)
Belgium_Female	3	bell-shaped; decelaration/level from 1979	Recent trend	1979	1992	ARIMA(0,1,0)
Belgium_Male	5	bell shaped; decelaration from 1984	Recent trend	1984	1990	ARIMA(0,1,0)
Germany_Female	7	bell shaped; increase from 1979	Recent trend	1979	1988	ARIMA(0,1,0)
Germany_Male	7	bell shaped; increase from 1979	Recent trend	1979	1988	ARIMA(0,1,0)
Hungary_Female	3	bell shaped; increase from 1974 (stable from 1985)	Recent trend	1974	1991	ARIMA(0,1,0)
Hungary_Male	3	bell shaped; increase/fluctuating from 1978 onwards	Recent trend	1978	1991	ARIMA(0,1,0)
Iceland_Male	3	bell shaped; level from 1984	Recent trend	1984	1993	ARIMA(0,1,0)
Ireland_Female	5	bell shaped; increase from 1974	Recent trend	1974	1989	ARIMA(0,1,0)
Switzerland_Male	7	bell shaped; increase/fluctuating from 1966 onwards	Recent trend	1966	1989	ARIMA(0,1,0)
Ireland_Male	5	recent decline (from 1967 onwards)	Recent trend	1967	1989	ARIMA(0,1,0) with drift
Poland_Male	5	recent decline (from 1969 onwards)	Recent trend	1969	1989	ARIMA(1,1,0) with drift
Slovenia_Female	3	recent decline (from 1975 onwards)	Recent trend	1975	1991	ARIMA(1,1,0) with drift
Slovenia_Male	3	recent decline (from 1974 onwards)	Recent trend	1975	1991	ARIMA(1,1,0) with drift
Spain_Male	5	recent decline (from 1976 onwards)	Recent trend	1976	1989	ARIMA(0,1,0) with drift
Czech Republic_Male	7	fluctuating around 0	Mean reverting process	1913	1989	ARIMA(2,0,2) with zero mean
France_Female	7	fluctuating around 0	Mean reverting process	1913	1988	ARIMA(2,0,1) with zero mean
France_Male	7	fluctuating around 0	Mean reverting process	1913	1988	ARIMA(2,0,0) with zero mean
Greece_Female	5	fluctuating around 0	Mean reverting process	1911	1988	ARIMA(2,0,2) with zero mean
Greece_Male	7	fluctuating around 0	Mean reverting process	1913	1986	ARIMA(1,0,2) with zero mean
Luxembourg_Female	7	fluctuating around 0	Mean reverting process	1913	1987	ARIMA(2,0,1) with zero mean
Luxembourg_Male	3	fluctuating around 0	Mean reverting process	1909	1990	ARIMA(2,0,1) with zero mean
Portugal_Male	7	fluctuating around 0	Mean reverting process	1913	1988	ARIMA(2,0,1) with zero mean
Spain_Female	7	fluctuating around 0	Mean reverting process	1913	1987	ARIMA(2,0,2) with zero mean
Switzerland_Female	3	fluctuating around 0	Mean reverting process	1909	1993	ARIMA(3,0,2) with zero mean
Italy_Female	5	u-shaped	Last values	1911	1989	ARIMA(0,1,0)
Italy_Male	7	u-shaped	Last values	1913	1987	ARIMA(0,1,0)
Portugal_Female	7	u-shaped	Last values	1913	1988	ARIMA(0,1,0)

Main outcomes

We projected age-specific and age-standardised alcohol-attributable mortality fractions (20-84) by sex, country, and year up to 2060 by means of medians and their 95% projection intervals by performing 50,000 simulations. Median age-standardised AAMF and their 95% projection intervals were obtained by age-standardising each sample path.

- **Simulations**

For the deterministic quadratic curve projections of the period parameter k_t, we obtained correlated errors and related prediction intervals by applying the best-fitting mean-reverting process to the errors (i.e., the difference between the observed and the fitted values). In doing so, we restricted p to maximally two, and q to maximally four. Also, we avoided the ARIMA(0,0,0) model, as this approach would not result in correlated errors. In these cases, we chose the best model (based on the AICc) out of two options: ARIMA(1,0,0) or ARIMA(1,0,1). See Table S2.

For each of the simulations, we projected the period and the cohort trends independently, which, together with the age pattern, formed a single forecast sample path. The point forecast of $AAMF_{x,s}$ was then given by the median over the generated 50,000 sample paths, and the 95% prediction intervals were obtained by calculating the appropriate quantiles. To construct the forecasts, we did not take into account the parameter uncertainty in the age, period, and cohort parameters (a_x, $k_{t,}$, and g_c in the observed period are taken as known, not estimated, values). Point forecasts and projection intervals for the age-standardised alcohol-attributable mortality fractions were obtained by age-standardising over each sample path separately.

- **Full projections by country and sex**

For the full projections by country and sex, including the projection of the period and cohort parameter, please see the two supplementary PDF files.
The fitted age-specific and fitted age-standardised AAMF values represent the fitted values in which the burned estimates for the youngest cohorts are replaced with the projected cohort values.
Regarding the age-specific AAMF plots, it should be noted that we compared the observed values for a five-year age group with the fitted value for a single year of age. For example, for the 20-24 age group, we compared the observed value for this age group with the fitted value for age 22, whereas the average age for the age group was 22.5. This approach led to small differences. It should also be noted when appraising the age-specific fit that for the older age groups in particular, the AAMF values are very much zoomed in.

Software

For our analysis, we used the R software version 3.6.2 in R Studio 1.2.5033.

References

Box GE, Jenkins GM, Reinsel GC, Ljung GM (2015) Time series analysis: forecasting and control. John Wiley & Sons.

Cairns AJ, Blake D, Dowd K, Coughlan GD, Epstein D, Ong A et al. (2009) A quantitative comparison of stochastic mortality models using data from England and Wales and the United States. *North Am Actuar J* 13(1):1–35.

Clayton D, Schifflers E (1987) Models for temporal variation in cancer rates. II: age–period–cohort models. *Stat Med* 6(4):469–81.

Corrao G, Bagnardi V, Zambon A, La Vecchia C (2004) A meta-analysis of alcohol consumption and the risk of 15 diseases. *Prev Med* 38:613-619.

Holmes MV, Dale CE, Zuccolo L, Silverwood RJ, Guo Y, Ye Z et al. (2014) Association between alcohol and cardiovascular disease: Mendelian randomisation analysis based on individual participant data. *BMJ* 349:g4164.

Human Cause-of-Death Database (2017) French Institute for Demographic Studies & Max Planck Institute for Demographic Research (Germany). Available at www.causeofdeath.org (accessed 30 June 2017).

Human Mortality Database (2018) University of California, Berkeley (USA), and Max Planck Institute for Demographic Research (Germany). Available at www.mortality.org (accessed 27 September 2018).

Hyndman R, Athanasopoulos G, Bergmeir C, Caceres G, Chhay L, O'Hara-Wild M et al. (2014) Forecast: Forecasting functions for time series and linear models. R package version 8.8. Available at http://pkg.robjhyndman.com/forecast.

Institute for Health Metrics and Evaluation (IHME) (2019). Global Burden of Disease Study 2017. GBD Results tool. Available online: https://gbd2017.healthdata.org/gbd-search/ (accessed 6 April 2018)

Manthey J, Rehm J (2019) Mortality from Alcoholic Cardiomyopathy: Exploring the Gap between Estimated and Civil Registry Data. *J Clin Med* 8(8):1137.

Rehm, J. (2010). Commentary on Rey et al.(2010): How to Improve Estimates on Alcohol-Attributable Burden?. *Addiction* 105(6):1030–1031.

Rehm J, Sulkowska U, Manczuk M, Boffetta P, Powles J, Popova S, et al. (2007) Alcohol accounts for a high proportion of premature mortality in central and eastern Europe. *Int J Epidemiol* 36:458-467.

Rey G, Jougla E (2014) Are alcohol-attributable mortality estimates reliable? *Eur J Public Health* 24:3-4.

Roerecke M, Rehm J (2012) The cardioprotective association of average alcohol consumption and ischaemic heart disease: a systematic review and meta-analysis. *Addiction* 107:1246-1260.

Semyonova VG, Gavrilova NS, Sabgayda TP, Antonova OM, Nikitina SY, Evdokushkina GN (2014) Approaches to the Assessment of Alcohol-Related Losses in the Russian Population. In: Anson J, Luy M, eds. Mortality in an International Perspective. Cham: Springer International Publishing, pp. 137–68.

Stanaway JD, Afshin A, Gakidou E, Lim SS, Abate D, Abate KH et al. (2018) Global, regional, and national comparative risk assessment of 84 behavioural, environmental and occupational, and metabolic risks or clusters of risks for 195 countries and territories, 1990–2017: a systematic analysis for the Global Burden of Disease Study 2017. *Lancet* 392(10159):1923–94.

Trias-Llimós S, Martikainen P, Mäkelä P, Janssen F (2018) Comparison of different approaches for estimating age-specific alcohol-attributable mortality: The cases of France and Finland. *PLoS ONE* 13(3):e0194478.

Villegas A, Kaishev VK, Millossovich P (2015) StMoMo: An R package for stochastic mortality modelling. Presented at the 7th Australasian Actuarial Education and Research Symposium.

World Health Organization (2018) WHO Mortality Database. Available at http://www.who.int/healthinfo/statistics/mortality_rawdata/en/ (accessed 11 April 2018).

Appendix A1 - Comparison of our adjusted age-specific and age-standardised alcohol-attributable mortality rates with the respective original GBD rates

Appendix Figure A1 – Comparison of the age-specific alcohol-attributable mortality rates in 2014 (or latest available year), original GBD estimates versus our adjusted estimates, 30 European countries, by sex

a) Males

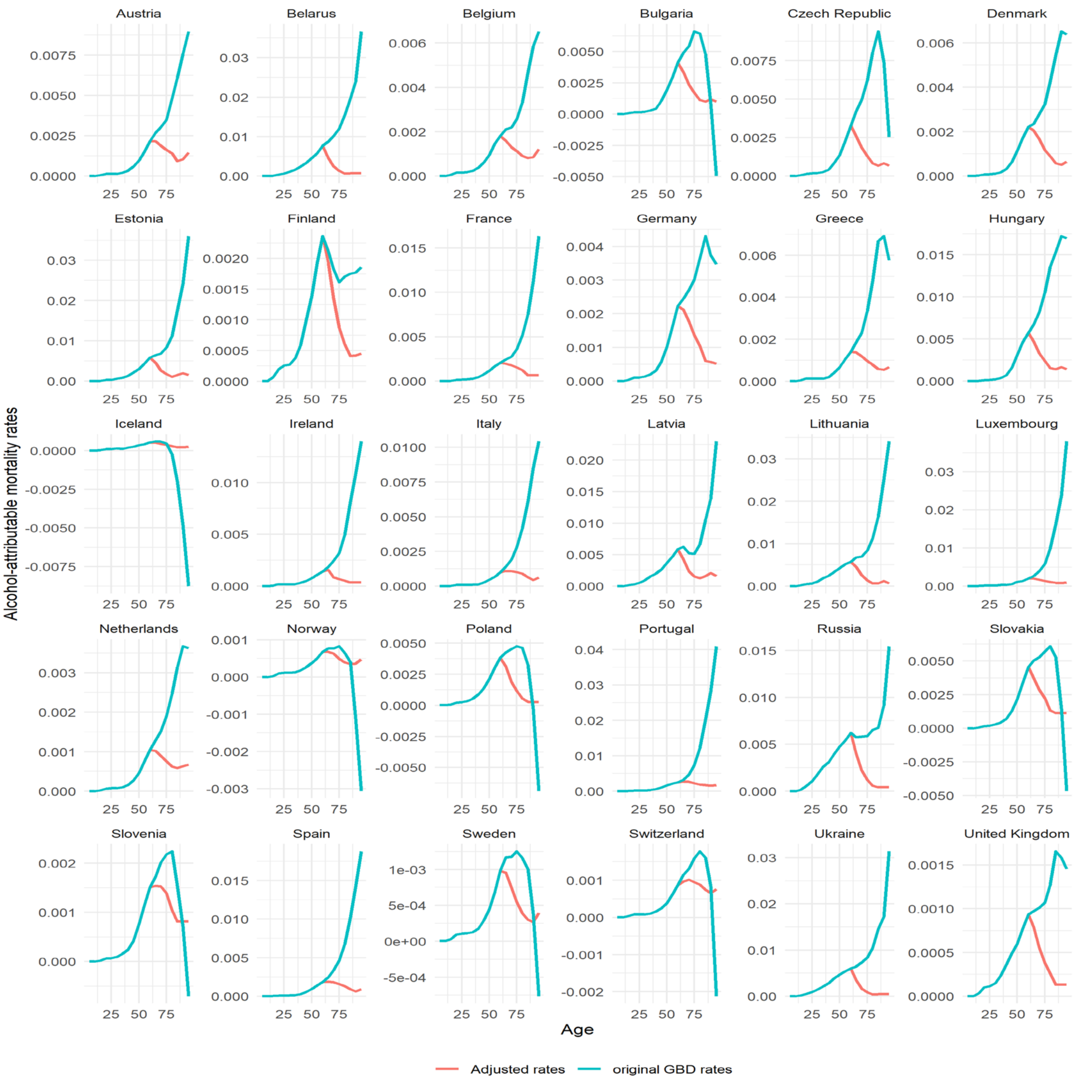

b) Females

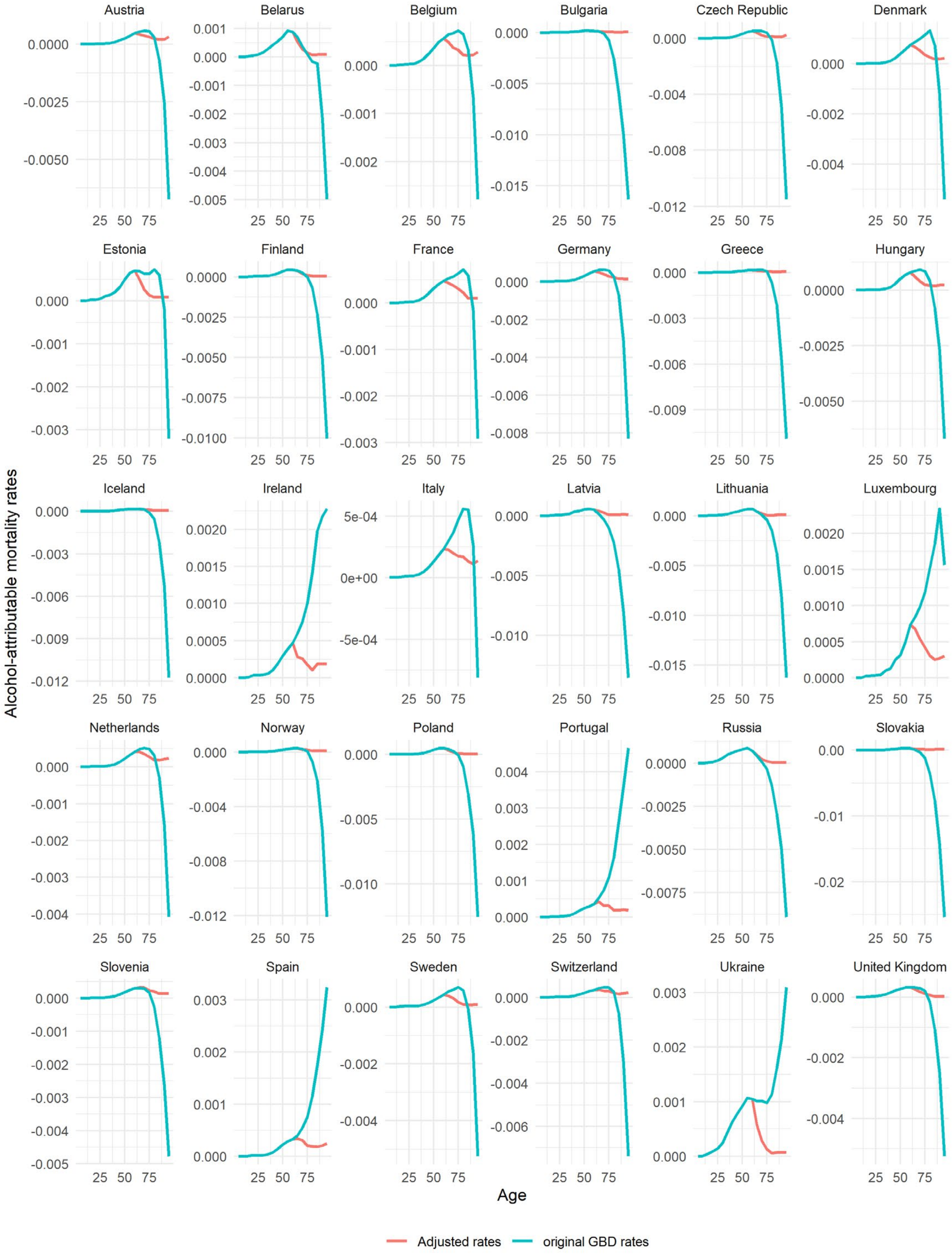
Austria
Belarus
Belgium
Bulgaria
Czech Republic
Denmark
Estonia
Finland
France
Germany
Greece
Hungary
Iceland
Ireland
Italy
Latvia
Lithuania
Luxembourg
Netherlands
Norway
Poland
Portugal
Russia
Slovakia
Slovenia
Spain
Sweden
Switzerland
Ukraine
United Kingdom
Alcohol-attributable mortality rates
Age
Adjusted rates
original GBD rates

Appendix Figure A2 – Comparison of the age-standardised* alcohol-attributable mortality rates (20-99), 1990-2014, either based on the original GBD estimates or based on our adjusted estimates, 30 European countries, by sex

a) Males

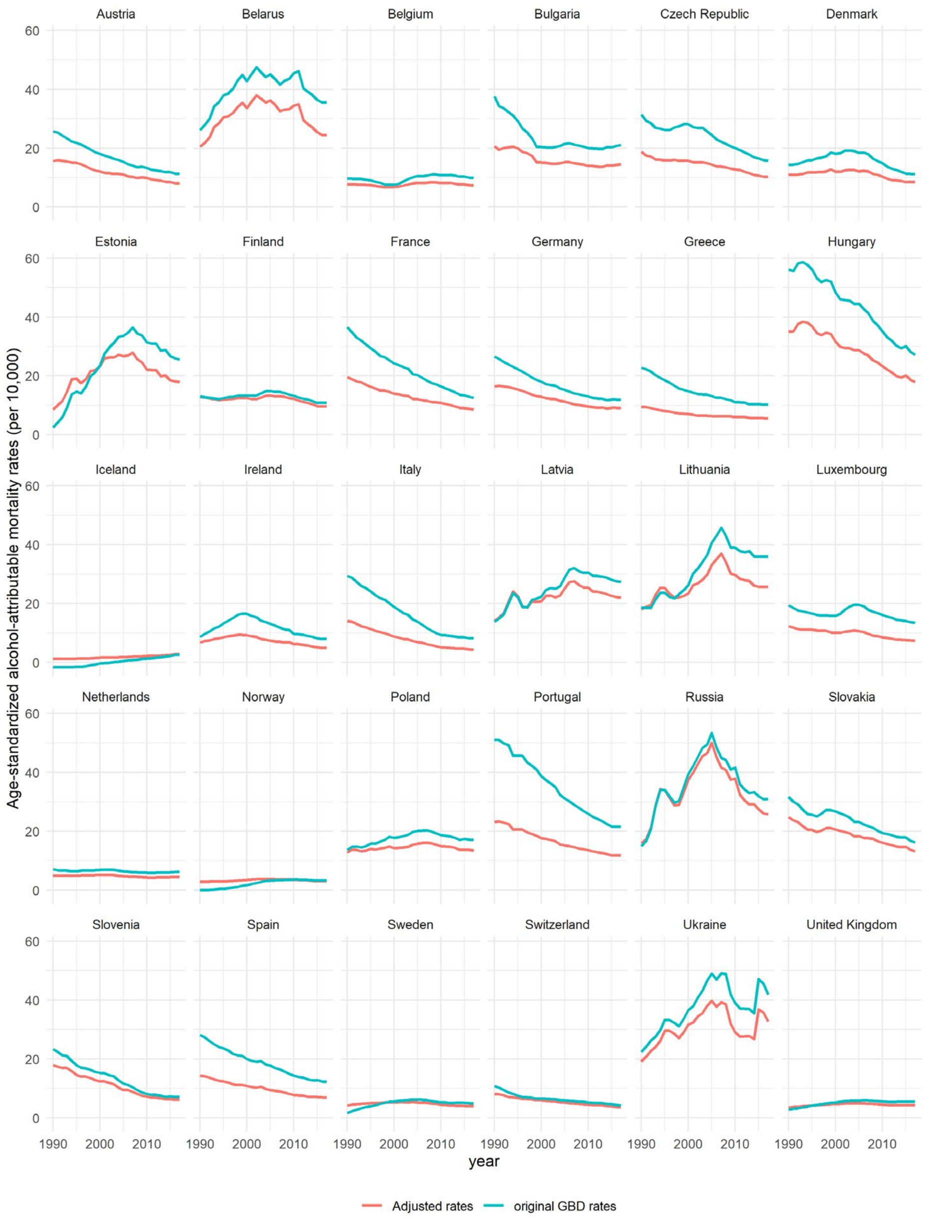

b) Females

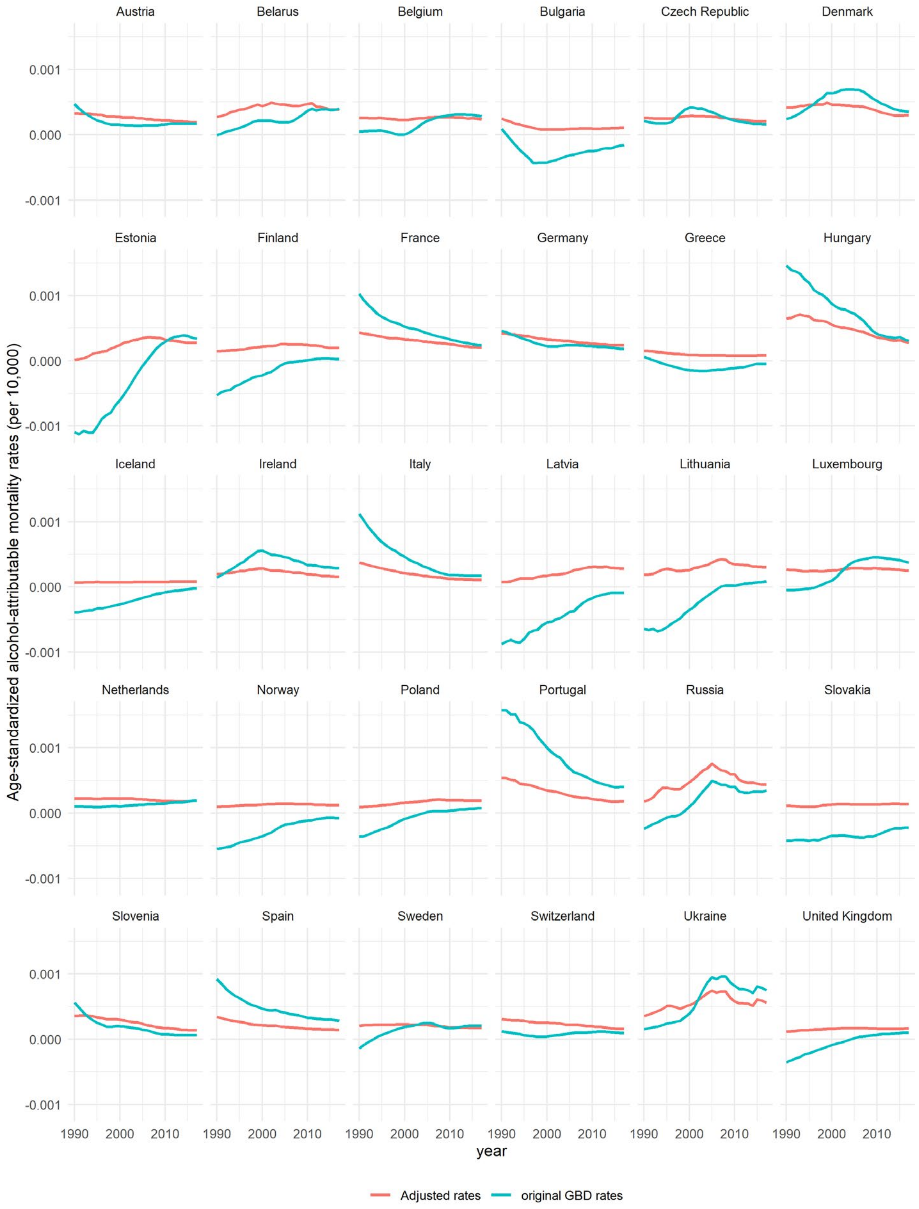

* Direct age standardisation was conducted using as the standard population the country and sex specific population distribution in 2010 (based on HMD data).
