# Supplementary material for: Past and Future Alcohol-Attributable Mortality in Europe
Source: Int J Environ Res Public Health. 2020 Dec 3;17(23):9024. doi: 10.3390/ijerph17239024 (PMC7730378; doi:10.3390/ijerph17239024)
Supplement: Supplementary file 1 [file ijerph-17-09024-s001.zip › Supplementary file 2. Detailed_projection_outcomes_men.pdf]

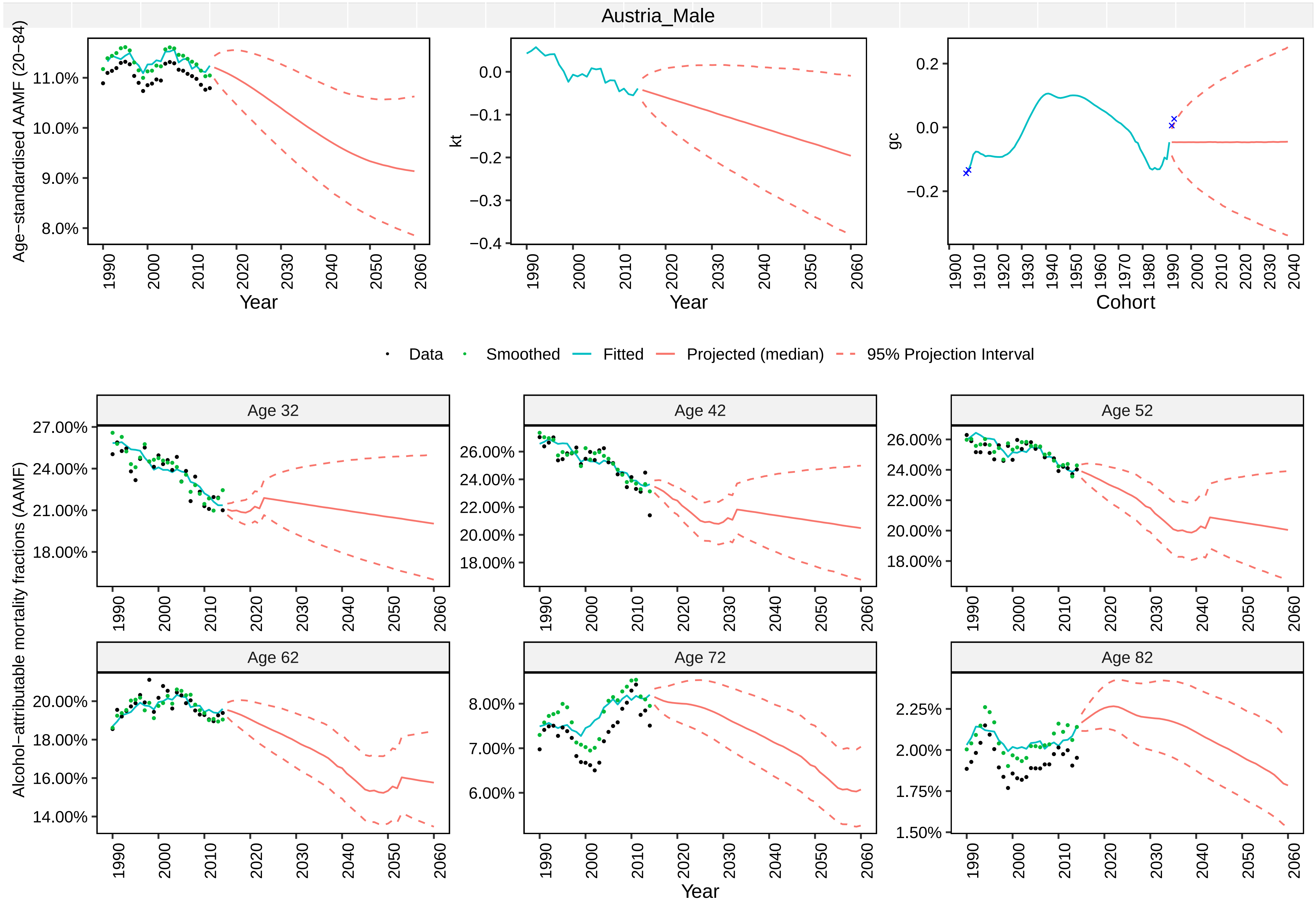
Austria_Male
Age−standardised AAMF (20−84)
11.0%
10.0%
9.0%
8.0%
Year
kt
0.0
−0.1
−0.2
−0.3
−0.4
gc
0.2
0.0
−0.2
Cohort
Data
Smoothed
Fitted
Projected (median)
95% Projection Interval
Age 32
Age 42
Age 52
Age 62
Age 72
Age 82
Alcohol−attributable mortality fractions (AAMF)
Year

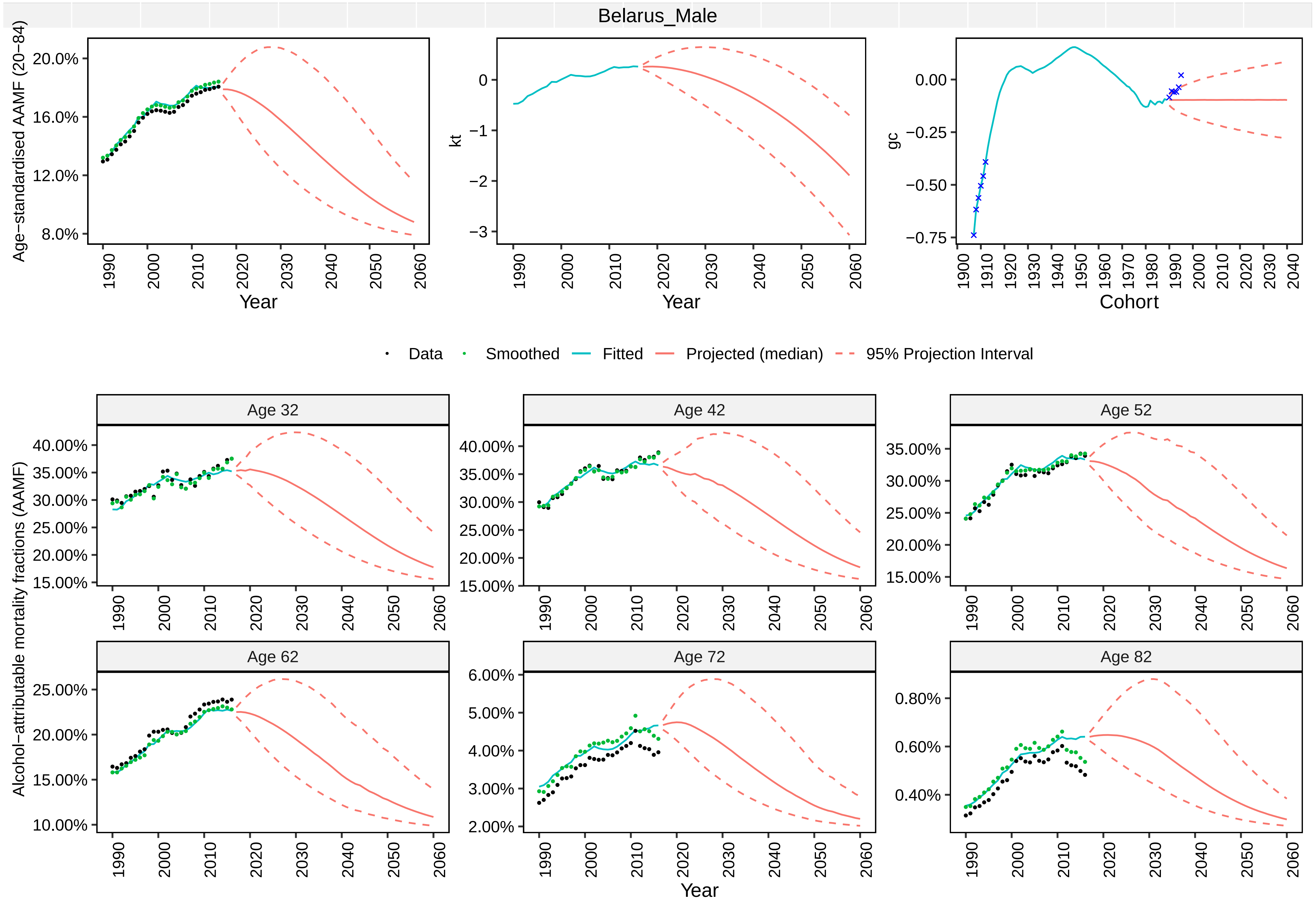

Belarus_Male
Age−standardised AAMF (20−84)
Year
kt
gc
Cohort
Data
Smoothed
Fitted
Projected (median)
95% Projection Interval
Age 32
Age 42
Age 52
Age 62
Age 72
Age 82
Alcohol−attributable mortality fractions (AAMF)

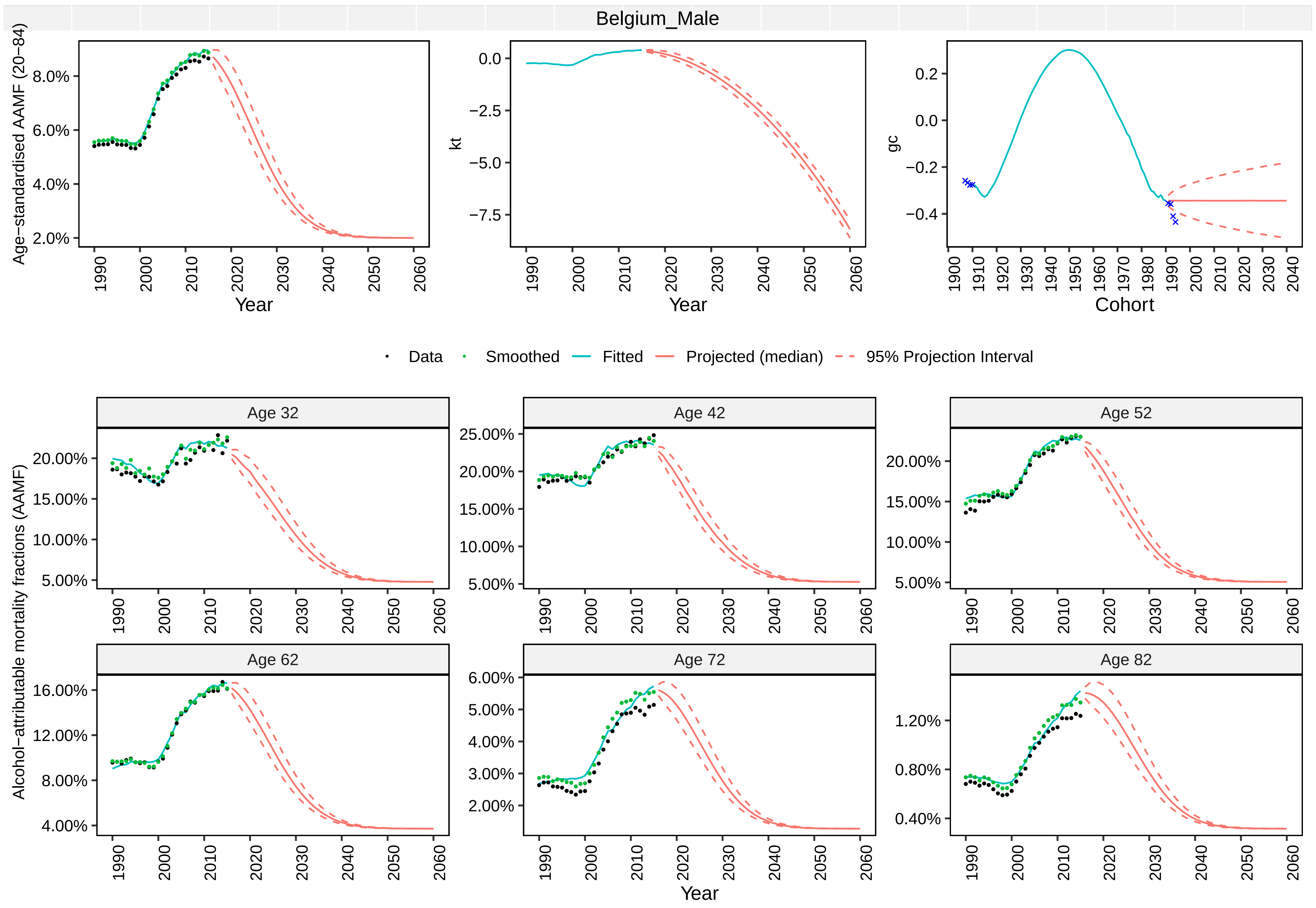
Belgium_Male
Age−standardised AAMF (20−84)
8.0%
6.0%
4.0%
2.0%
Year
kt
0.0
−2.5
−5.0
−7.5
gc
0.2
0.0
−0.2
−0.4
Cohort
Data
Smoothed
Fitted
Projected (median)
95% Projection Interval
Alcohol−attributable mortality fractions (AAMF)
Age 32
Age 42
Age 52
Age 62
Age 72
Age 82
20.00%
15.00%
10.00%
5.00%
25.00%
16.00%
12.00%
8.00%
4.00%
6.00%
5.00%
4.00%
3.00%
2.00%
1.20%
0.80%
0.40%
1990
2000
2010
2020
2030
2040
2050
2060
Year

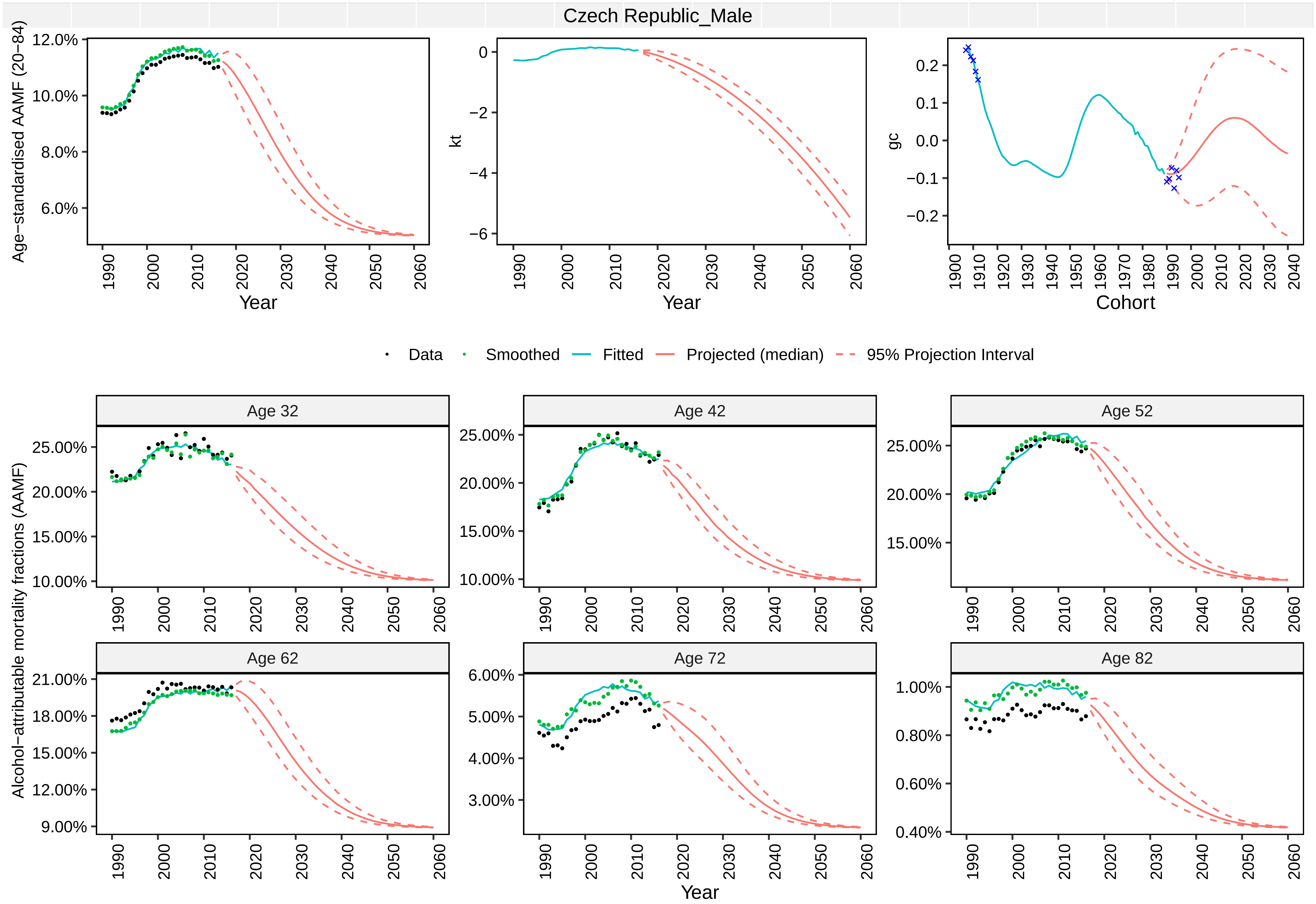
Czech Republic_Male
Age−standardised AAMF (20−84)
Year
kt
Year
gc
Cohort
Data
Smoothed
Fitted
Projected (median)
95% Projection Interval
Age 32
Age 42
Age 52
Age 62
Age 72
Age 82
Alcohol−attributable mortality fractions (AAMF)
Year

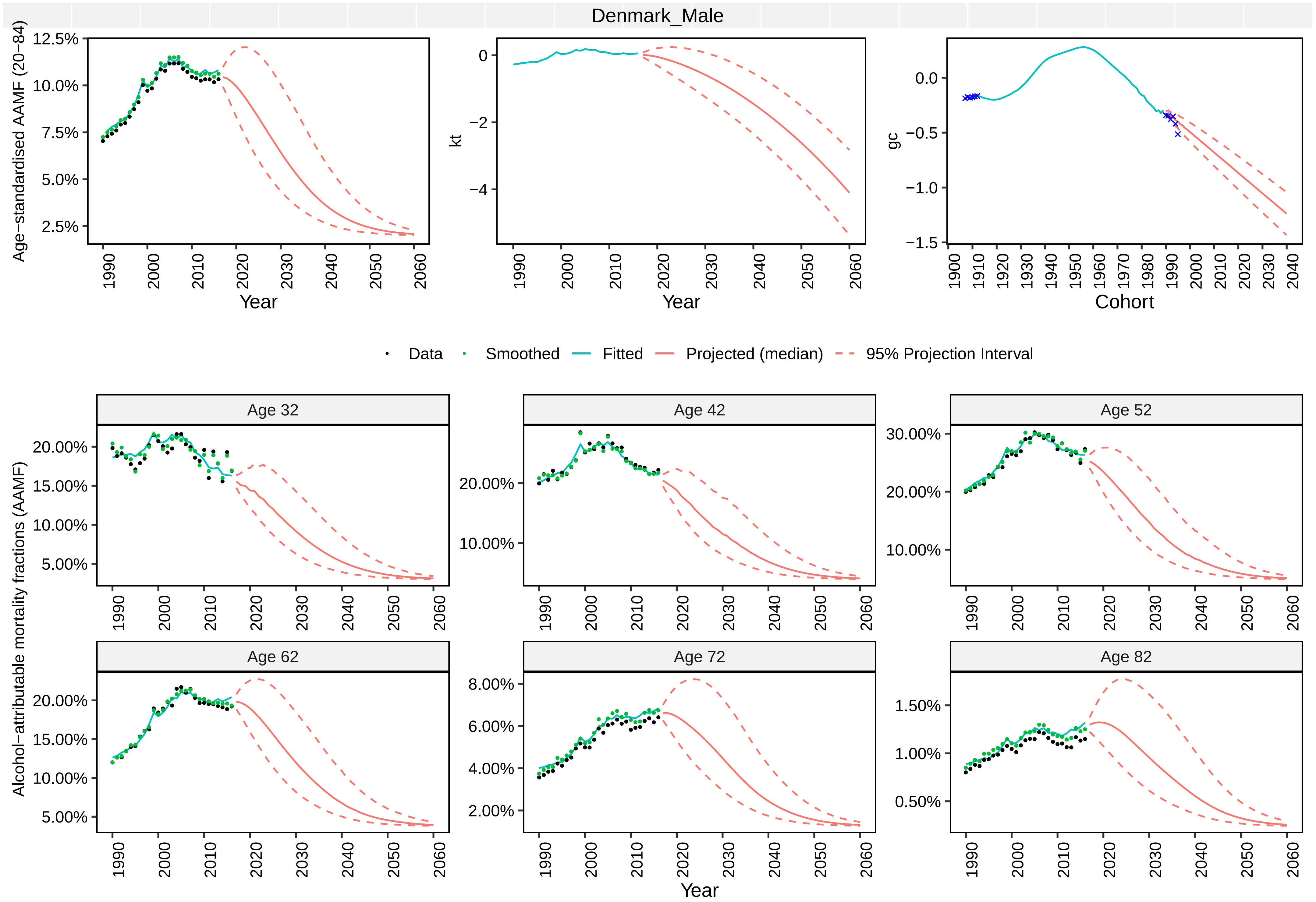
Denmark_Male
Age−standardised AAMF (20−84)
Year
kt
gc
Cohort
Data
Smoothed
Fitted
Projected (median)
95% Projection Interval
Age 32
Age 42
Age 52
Age 62
Age 72
Age 82
Alcohol−attributable mortality fractions (AAMF)
Year

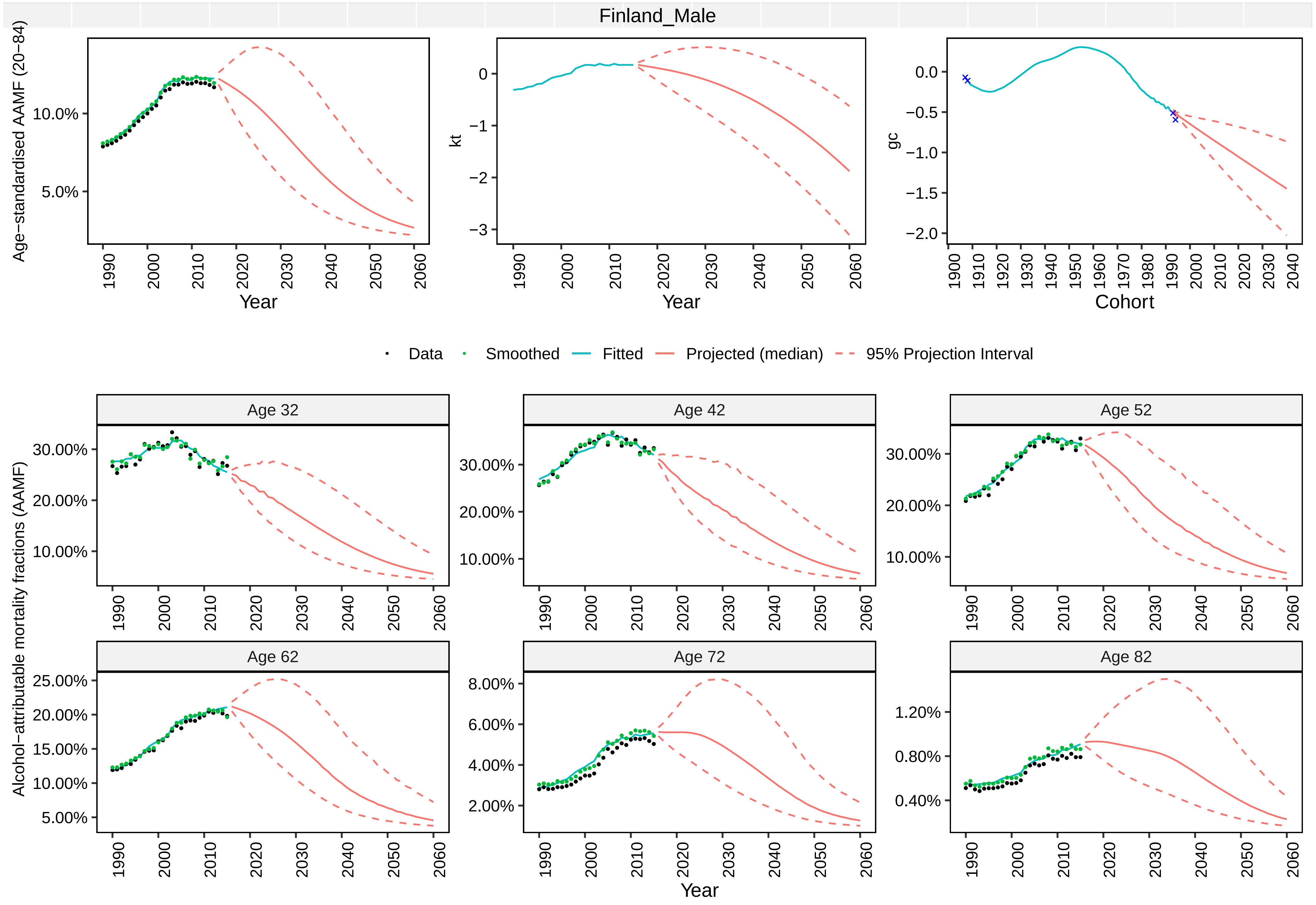
Finland_Male
Age−standardised AAMF (20−84)
10.0%
5.0%
Year
kt
0
−1
−2
−3
Year
gc
0.0
−0.5
−1.0
−1.5
−2.0
Cohort
Data
Smoothed
Fitted
Projected (median)
95% Projection Interval
Alcohol−attributable mortality fractions (AAMF)
Age 32
Age 42
Age 52
Age 62
Age 72
Age 82
30.00%
20.00%
10.00%
25.00%
15.00%
5.00%
8.00%
6.00%
4.00%
2.00%
1.20%
0.80%
0.40%
1990
2000
2010
2020
2030
2040
2050
2060
Year

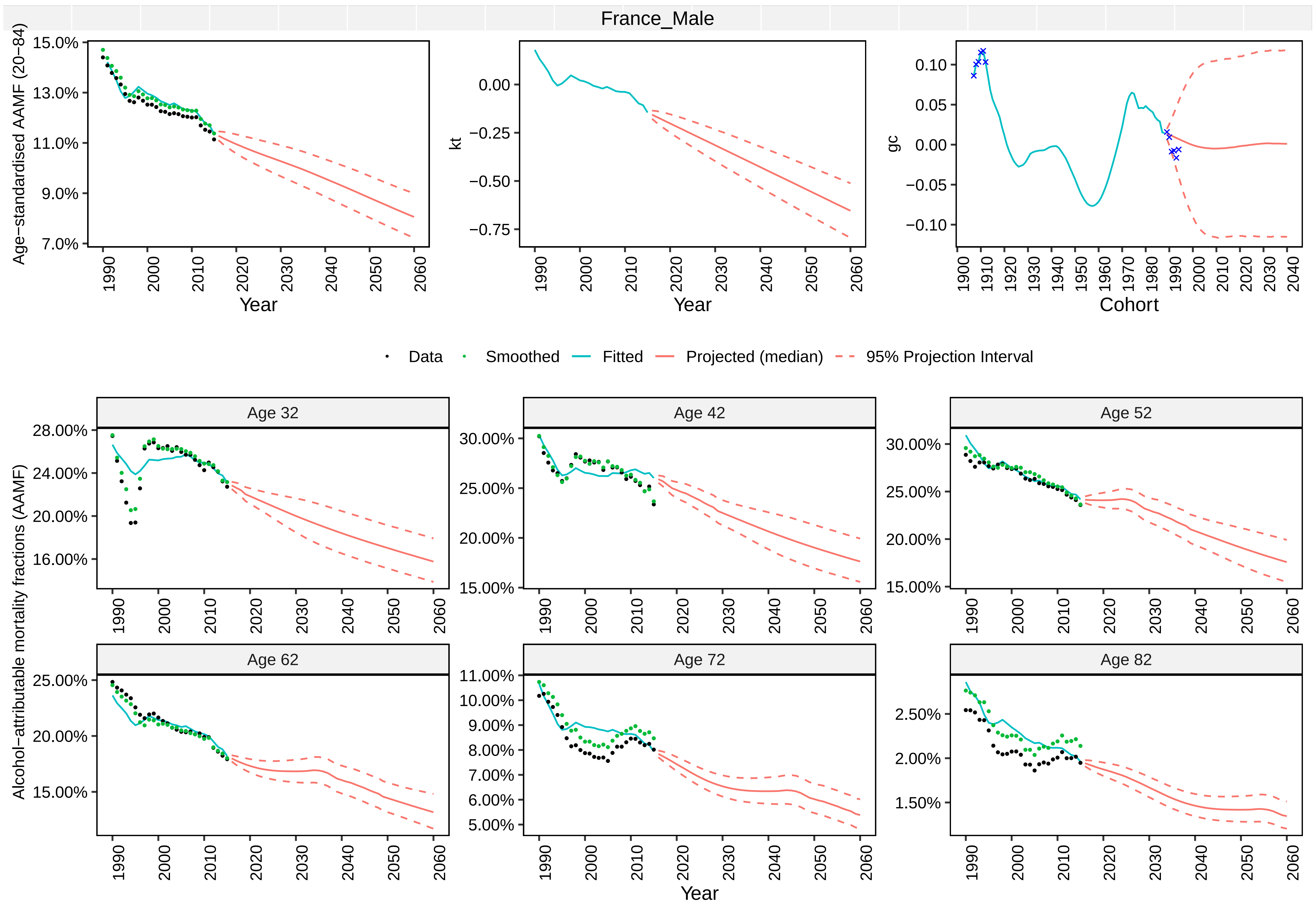
France_Male
Age−standardised AAMF (20−84)
15.0%
13.0%
11.0%
9.0%
7.0%
Year
kt
0.00
−0.25
−0.50
−0.75
gc
0.10
0.05
0.00
−0.05
−0.10
Cohort
Data
Smoothed
Fitted
Projected (median)
95% Projection Interval
Alcohol−attributable mortality fractions (AAMF)
Age 32
Age 42
Age 52
Age 62
Age 72
Age 82
Year

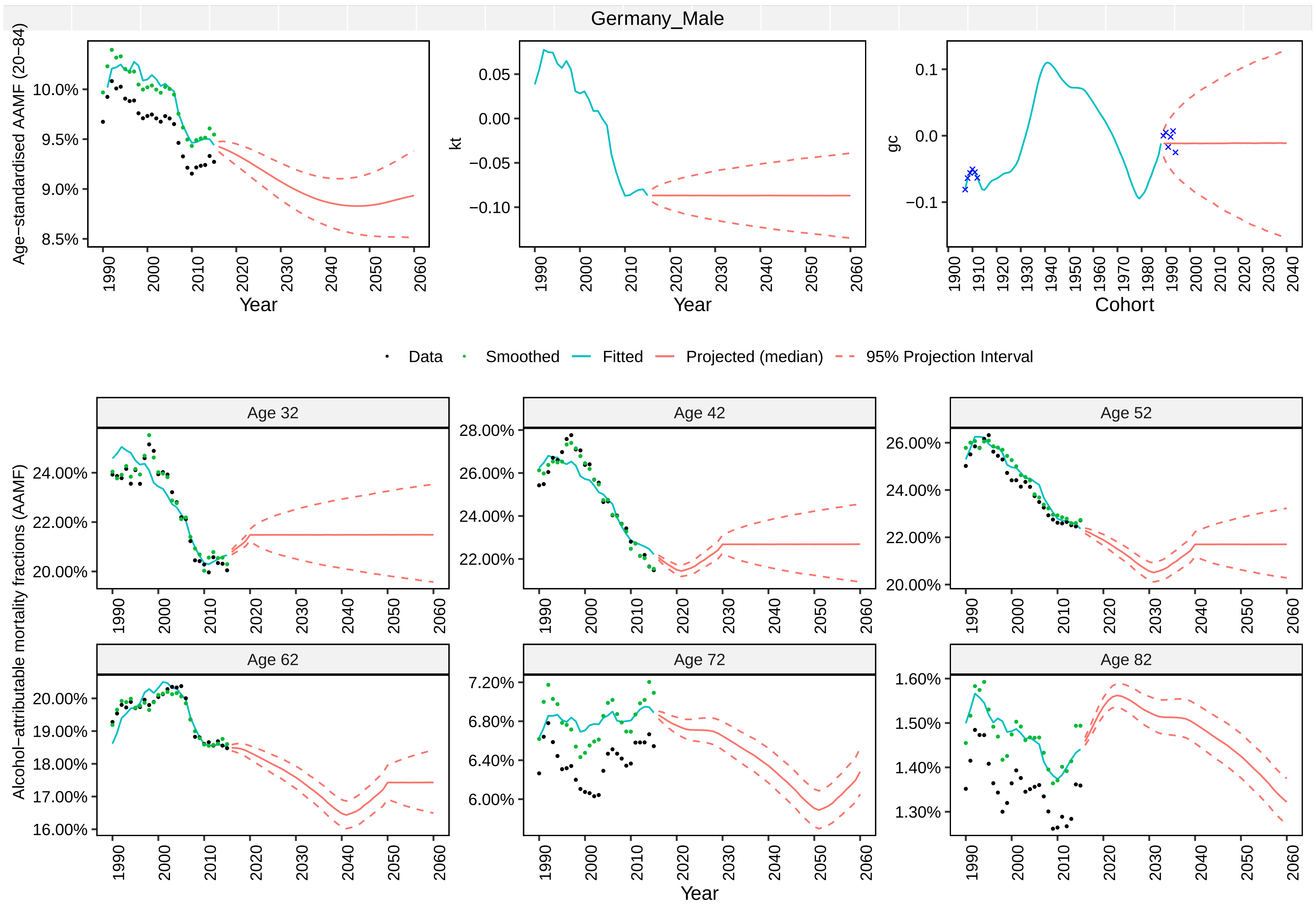
Germany_Male
Age−standardised AAMF (20−84)
kt
gc
Year
Cohort
Data
Smoothed
Fitted
Projected (median)
95% Projection Interval
Age 32
Age 42
Age 52
Age 62
Age 72
Age 82
Alcohol−attributable mortality fractions (AAMF)

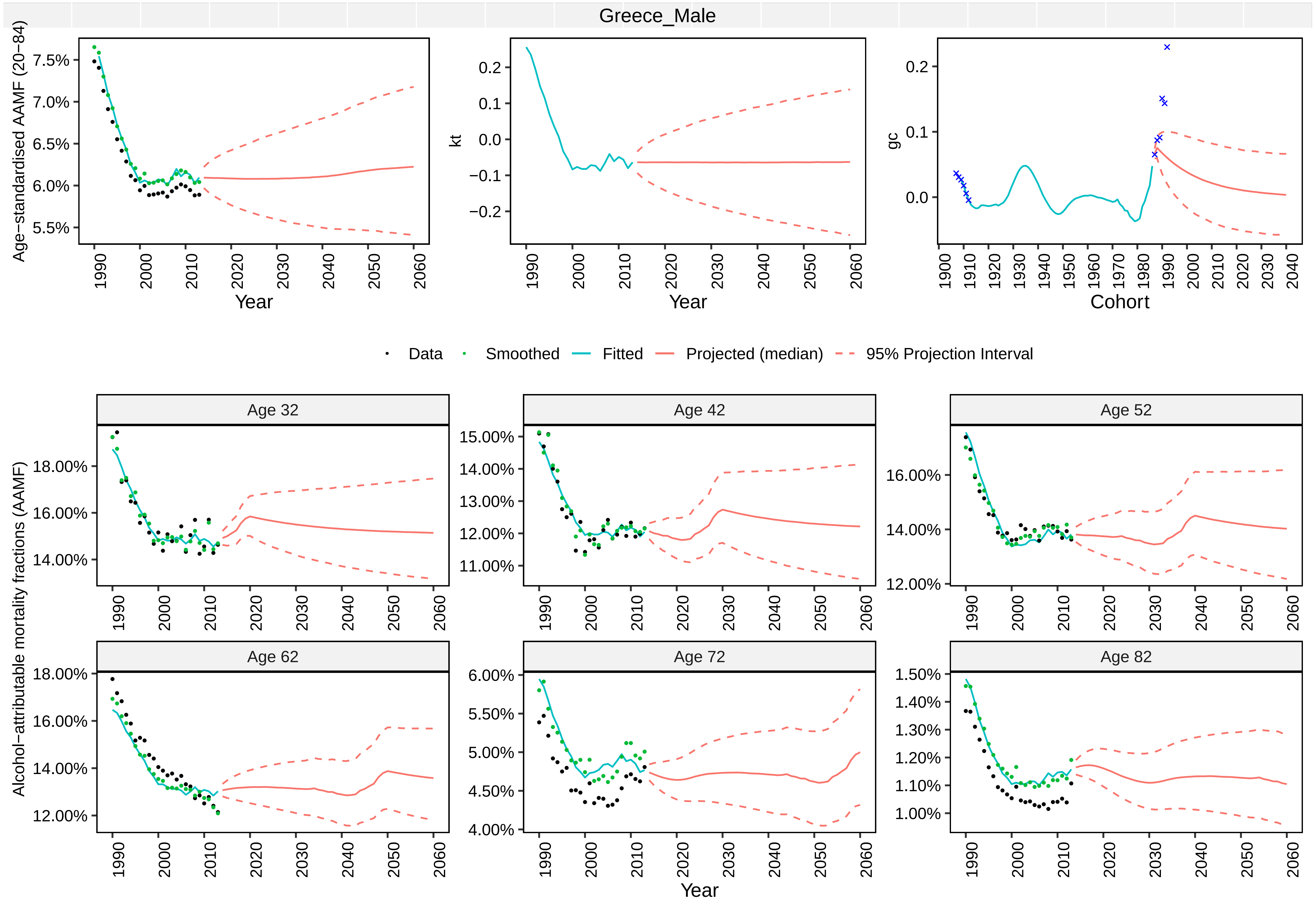
Greece_Male
Age−standardised AAMF (20−84)
7.5%
7.0%
6.5%
6.0%
5.5%
Year
kt
0.2
0.1
0.0
−0.1
−0.2
gc
Cohort
Data
Smoothed
Fitted
Projected (median)
95% Projection Interval
Age 32
Age 42
Age 52
Age 62
Age 72
Age 82
Alcohol−attributable mortality fractions (AAMF)
1990
2000
2010
2020
2030
2040
2050
2060

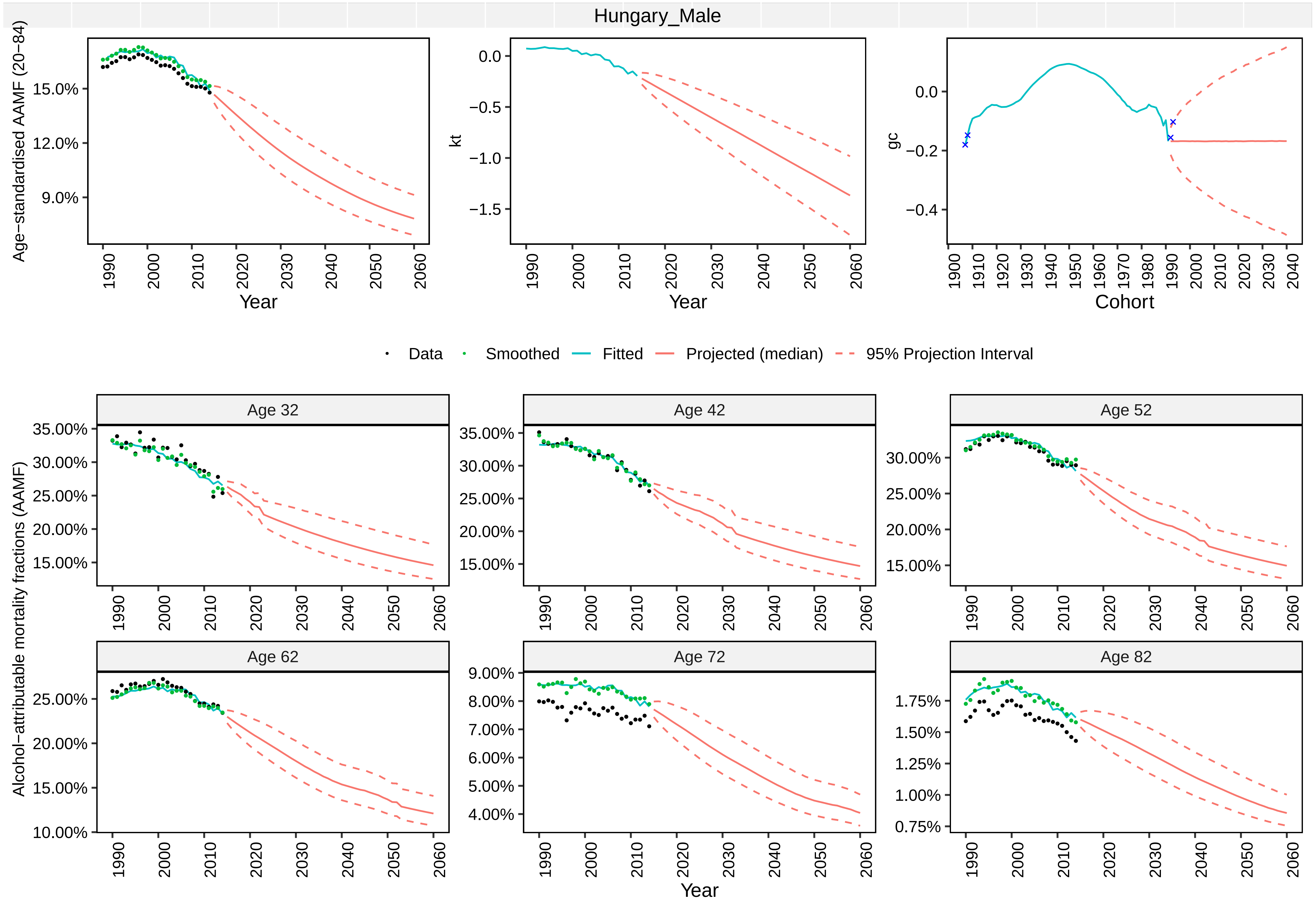

Hungary_Male
Age−standardised AAMF (20−84)
15.0%
12.0%
9.0%
Year
kt
0.0
−0.5
−1.0
−1.5
gc
0.0
−0.2
−0.4
Cohort
1990
2000
2010
2020
2030
2040
2050
2060
1900
1910
1920
1930
1940
1950
1960
1970
1980
1990
2000
2010
2020
2030
2040
Data
Smoothed
Fitted
Projected (median)
95% Projection Interval
Alcohol−attributable mortality fractions (AAMF)
Age 32
Age 42
Age 52
Age 62
Age 72
Age 82
35.00%
30.00%
25.00%
20.00%
15.00%
10.00%
9.00%
8.00%
7.00%
6.00%
5.00%
4.00%
1.75%
1.50%
1.25%
1.00%
0.75%
Year

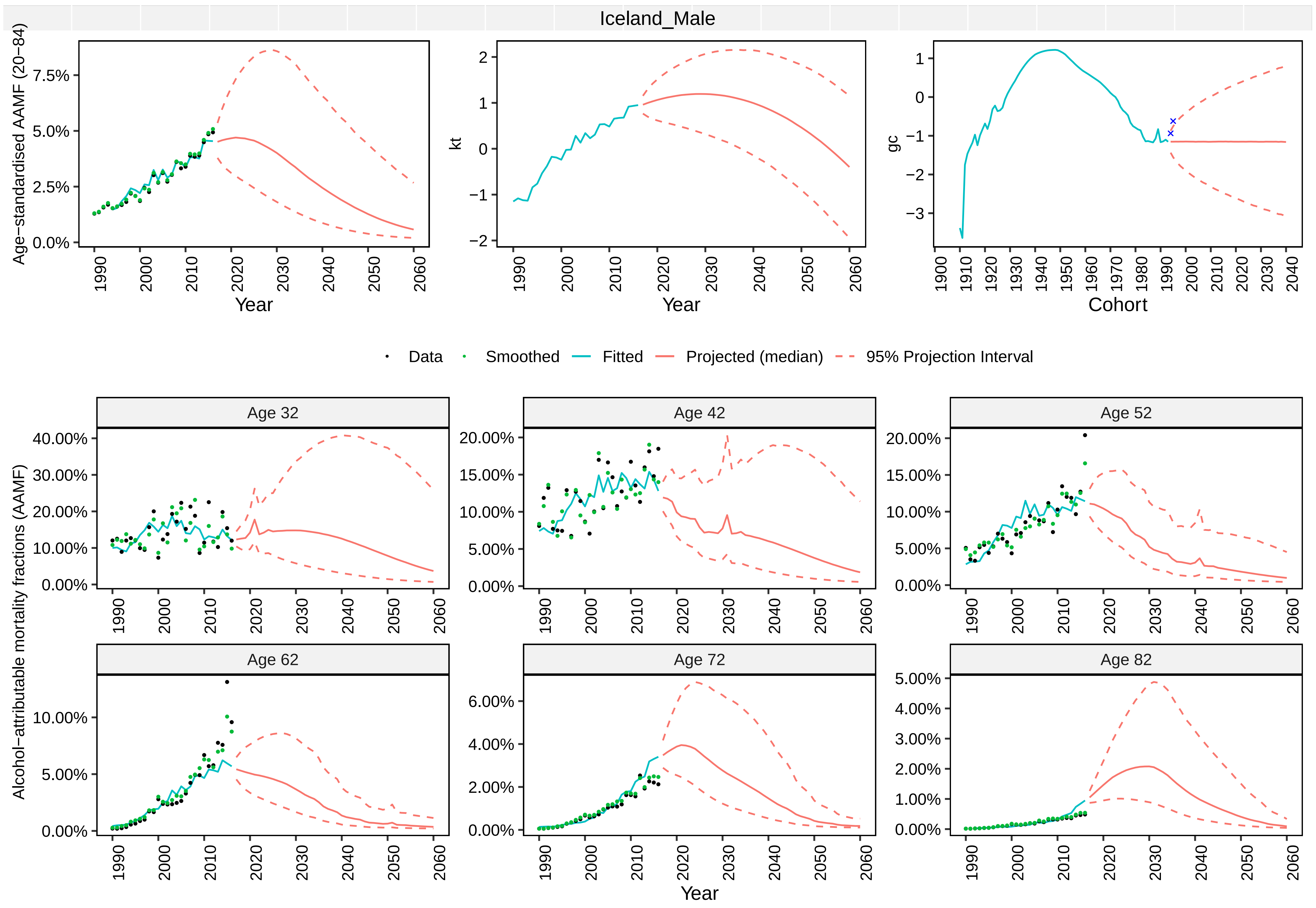

Iceland_Male
Age−standardised AAMF (20−84)
Year
kt
gc
Cohort
Data
Smoothed
Fitted
Projected (median)
95% Projection Interval
Age 32
Age 42
Age 52
Age 62
Age 72
Age 82
Alcohol−attributable mortality fractions (AAMF)

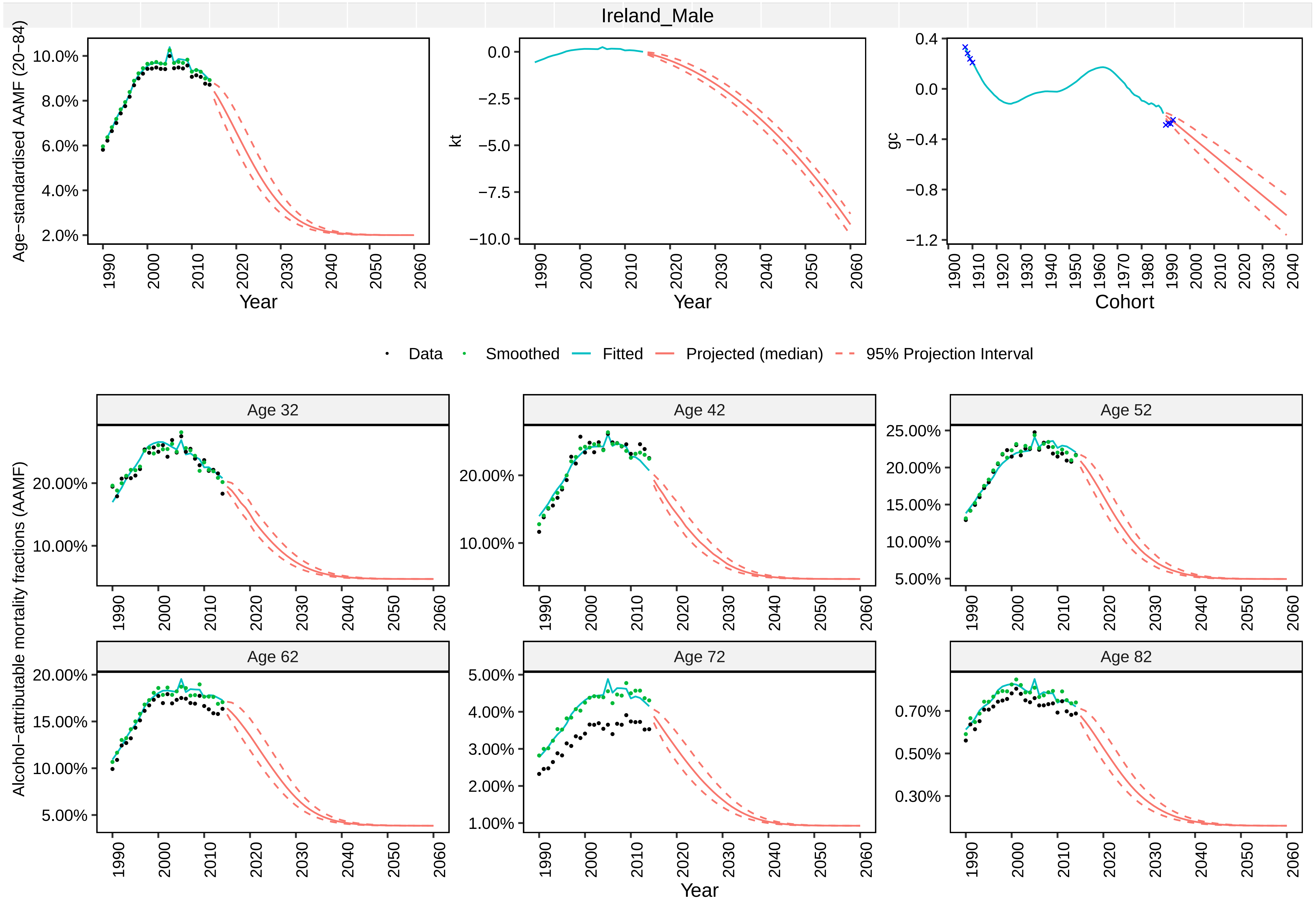

Ireland_Male
Age−standardised AAMF (20−84)
Year
kt
Year
gc
Cohort
Data
Smoothed
Fitted
Projected (median)
95% Projection Interval
Age 32
Age 42
Age 52
Age 62
Age 72
Age 82
Alcohol−attributable mortality fractions (AAMF)
Year

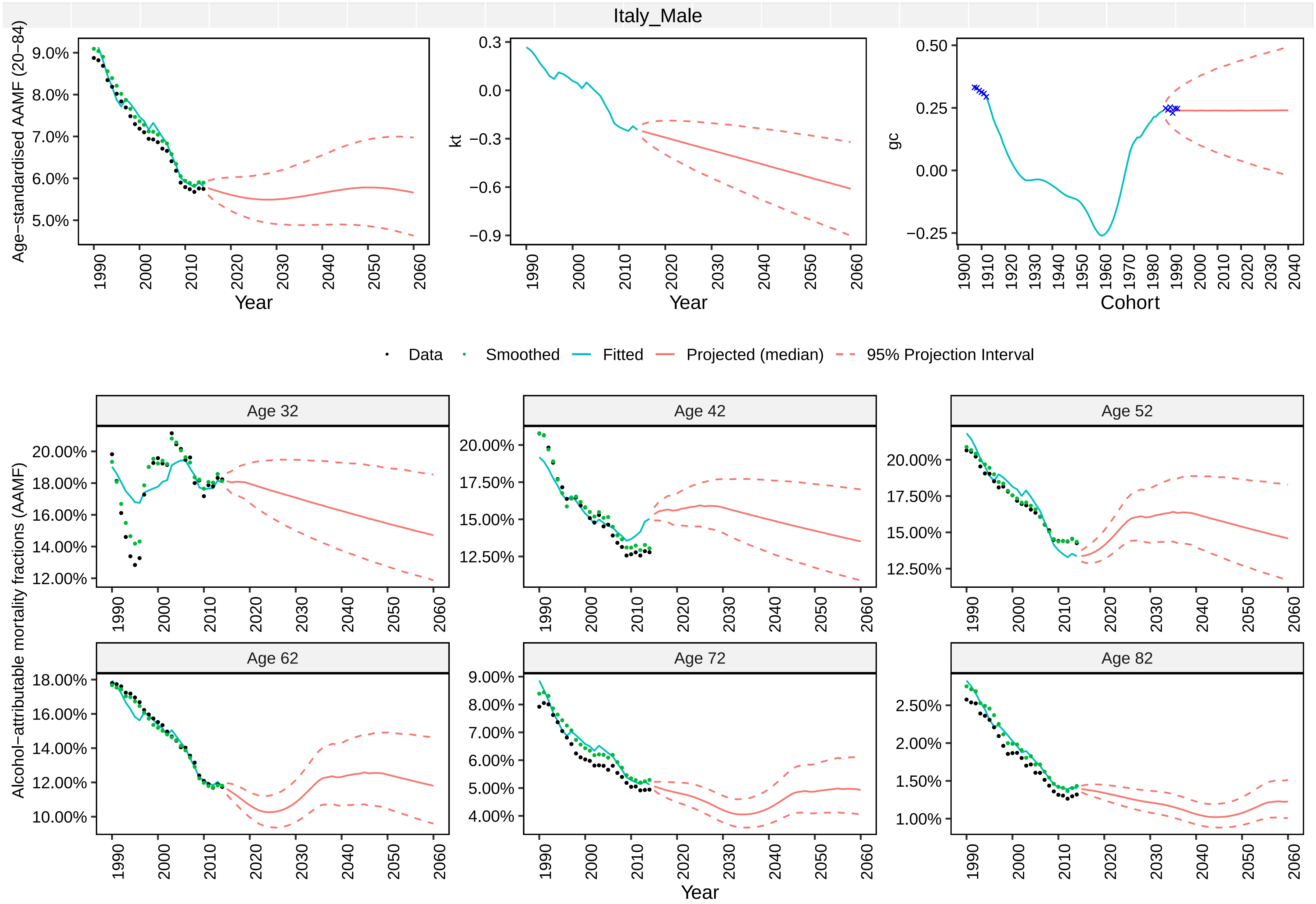
Italy_Male
Age−standardised AAMF (20−84)
kt
gc
Year
Cohort
Data
Smoothed
Fitted
Projected (median)
95% Projection Interval
Age 32
Age 42
Age 52
Age 62
Age 72
Age 82
Alcohol−attributable mortality fractions (AAMF)
Year

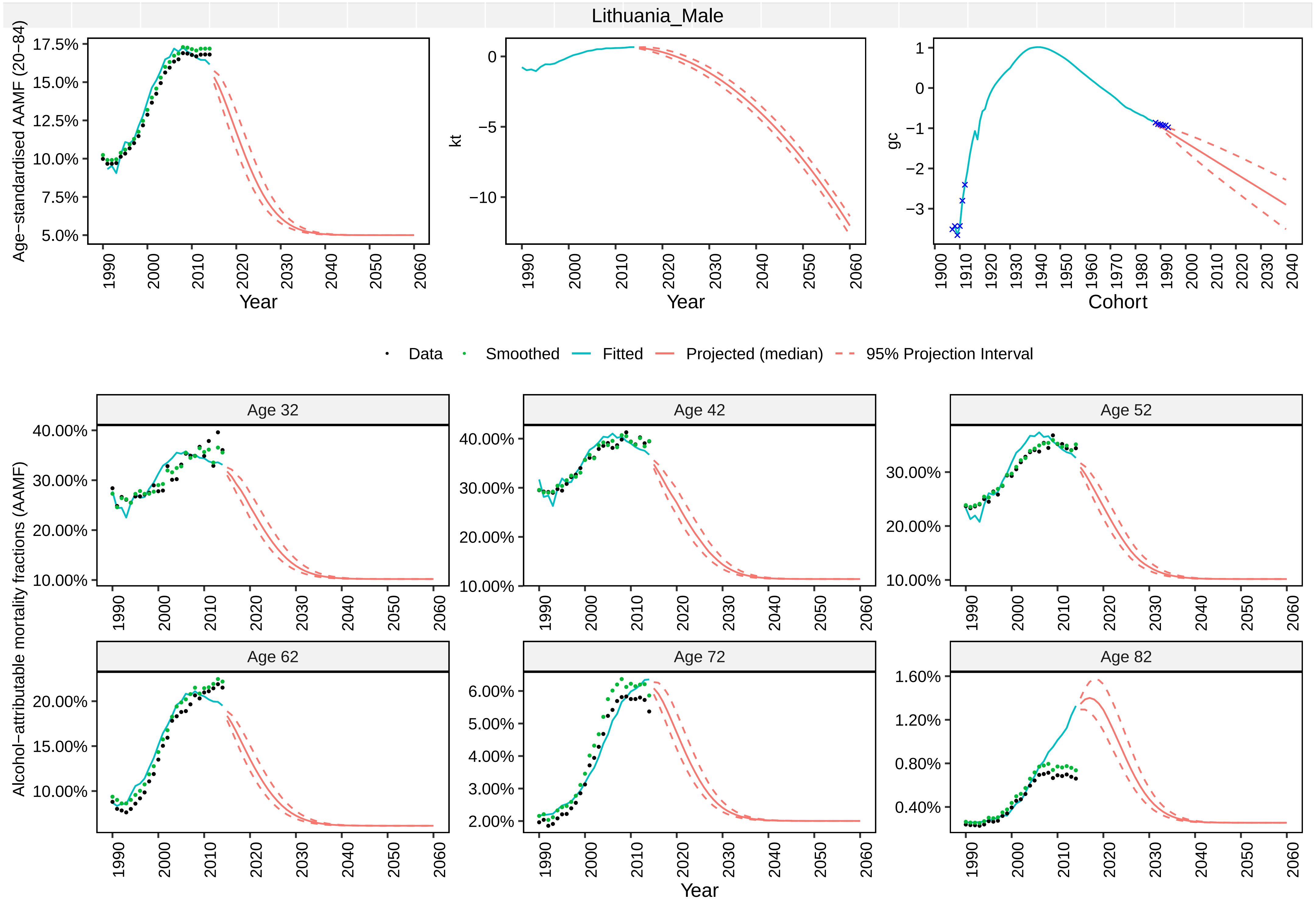

Lithuania_Male
Age−standardised AAMF (20−84)
17.5%
15.0%
12.5%
10.0%
7.5%
5.0%
Year
kt
0
−5
−10
gc
1
0
−1
−2
−3
Cohort
1990
2000
2010
2020
2030
2040
2050
2060
1900
1910
1920
1930
1940
1950
1960
1970
1980
1990
2000
2010
2020
2030
2040
Data
Smoothed
Fitted
Projected (median)
95% Projection Interval
Alcohol−attributable mortality fractions (AAMF)
Age 32
40.00%
30.00%
20.00%
10.00%
Age 42
Age 52
Age 62
20.00%
15.00%
10.00%
Age 72
6.00%
5.00%
4.00%
3.00%
2.00%
Age 82
1.60%
1.20%
0.80%
0.40%
Year

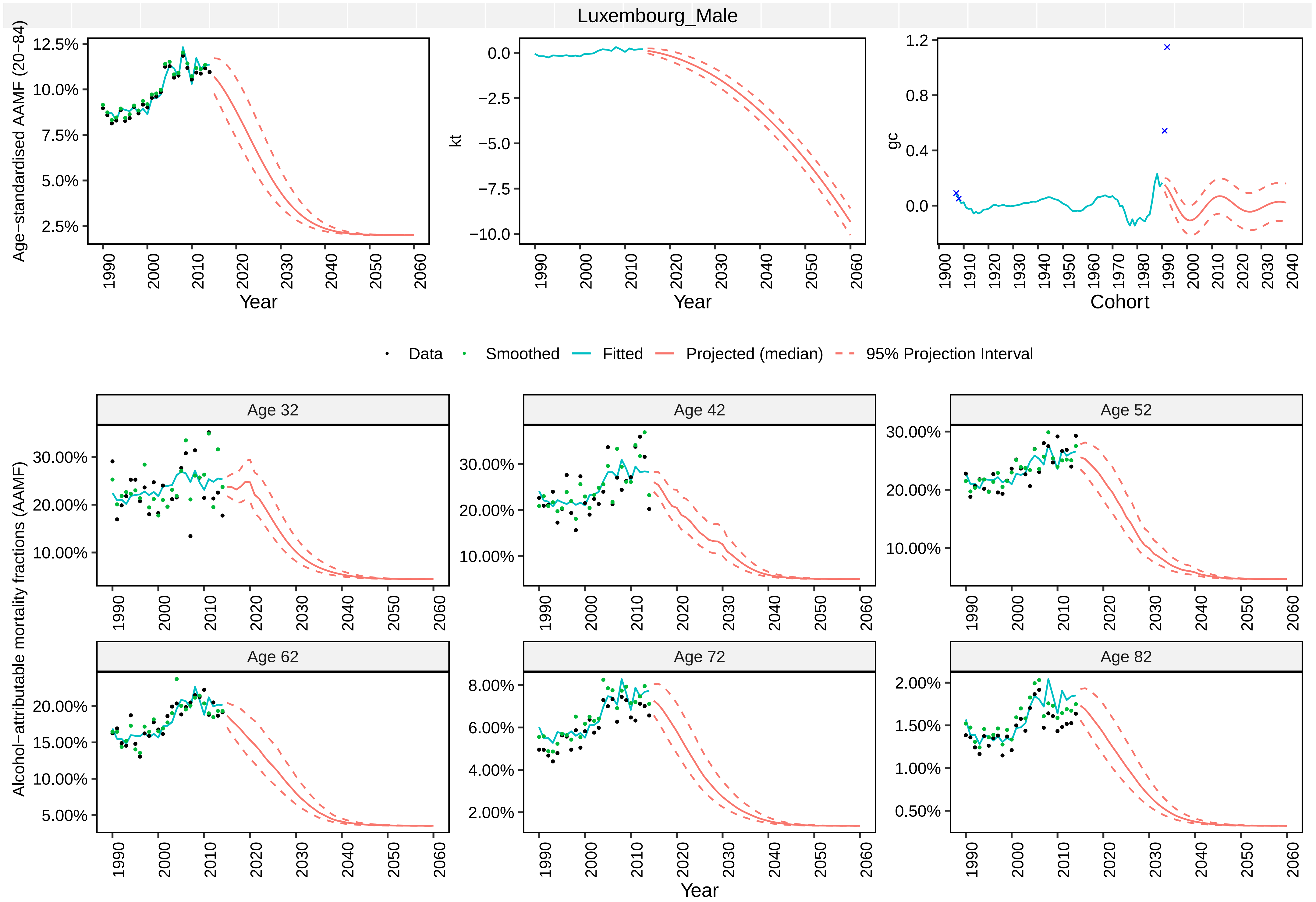

Luxembourg_Male
Age−standardised AAMF (20−84)
12.5%
10.0%
7.5%
5.0%
2.5%
1990
2000
2010
2020
2030
2040
2050
2060
Year
kt
0.0
−2.5
−5.0
−7.5
−10.0
gc
1.2
0.8
0.4
0.0
Cohort
1900
1910
1920
1930
1940
1950
1960
1970
1980
1990
2000
2010
2020
2030
2040
Data
Smoothed
Fitted
Projected (median)
95% Projection Interval
Alcohol−attributable mortality fractions (AAMF)
Age 32
Age 42
Age 52
Age 62
Age 72
Age 82
30.00%
20.00%
10.00%
15.00%
5.00%
8.00%
6.00%
4.00%
2.00%
2.00%
1.50%
1.00%
0.50%

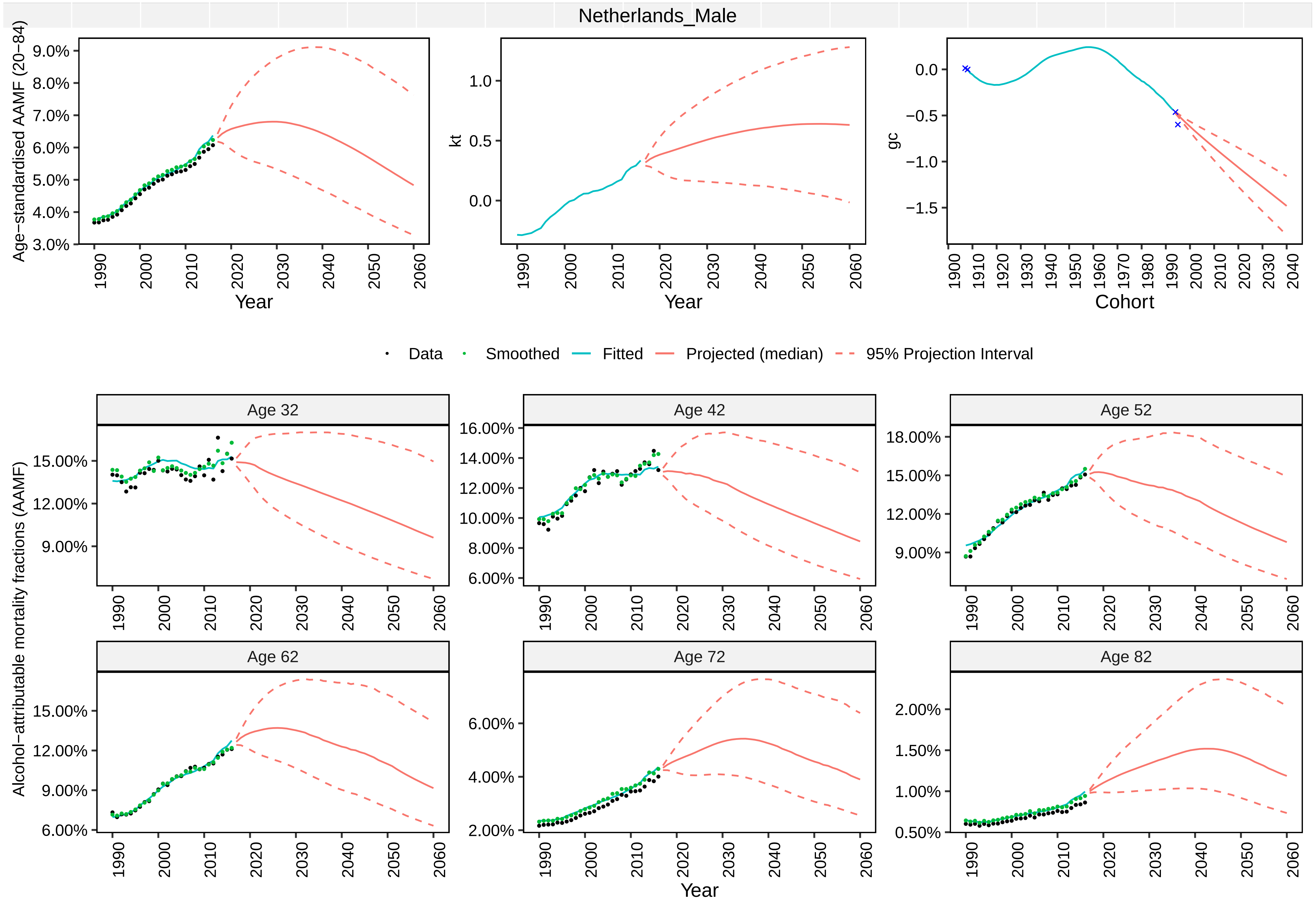
Netherlands_Male
Age−standardised AAMF (20−84)
Year
kt
Year
gc
Cohort
Data
Smoothed
Fitted
Projected (median)
95% Projection Interval
Age 32
Age 42
Age 52
Age 62
Age 72
Age 82
Alcohol−attributable mortality fractions (AAMF)
Year

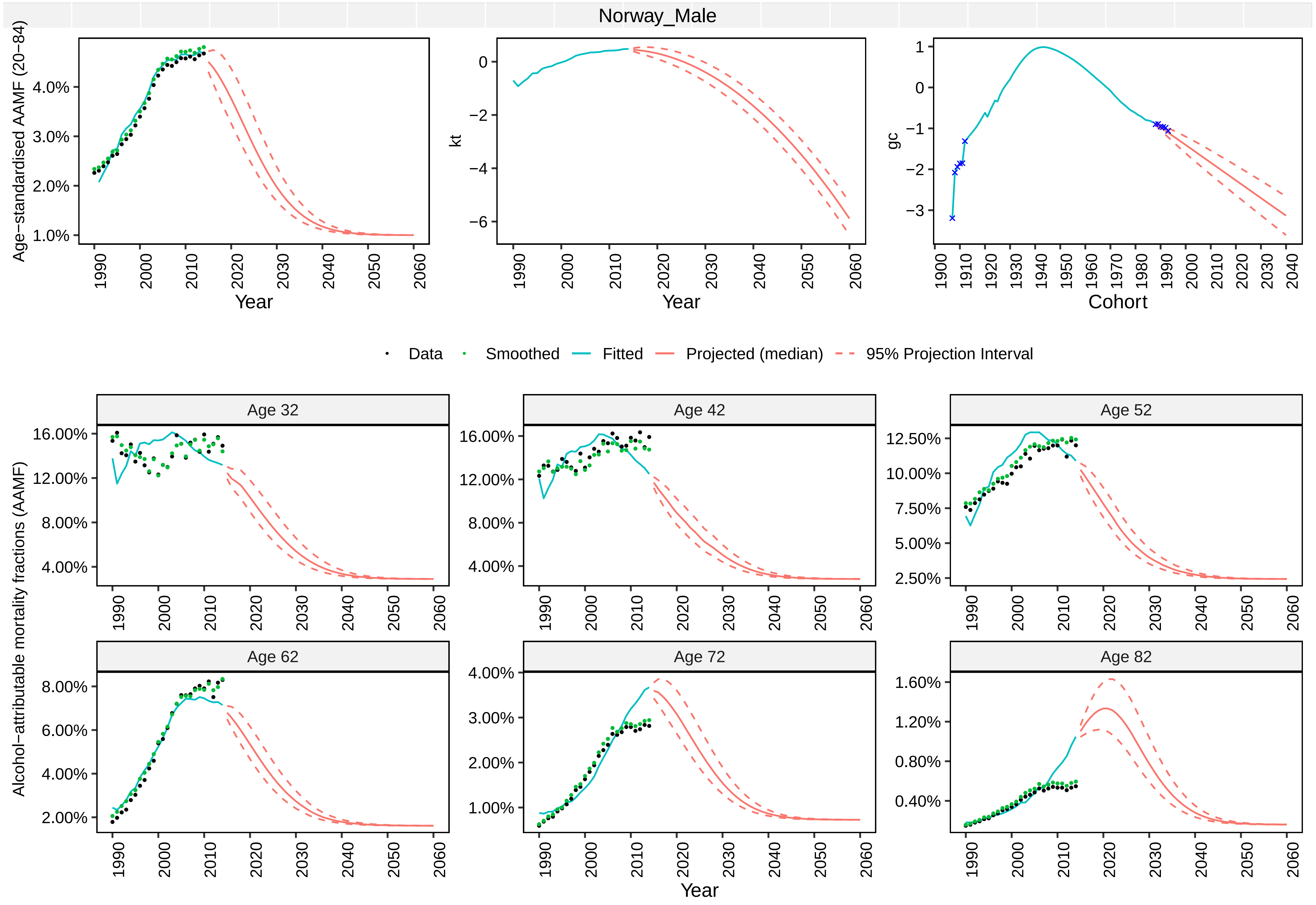

Norway_Male
Age−standardised AAMF (20−84)
Year
kt
Year
gc
Cohort
Data
Smoothed
Fitted
Projected (median)
95% Projection Interval
Alcohol−attributable mortality fractions (AAMF)
Age 32
Age 42
Age 52
Age 62
Age 72
Age 82
Year

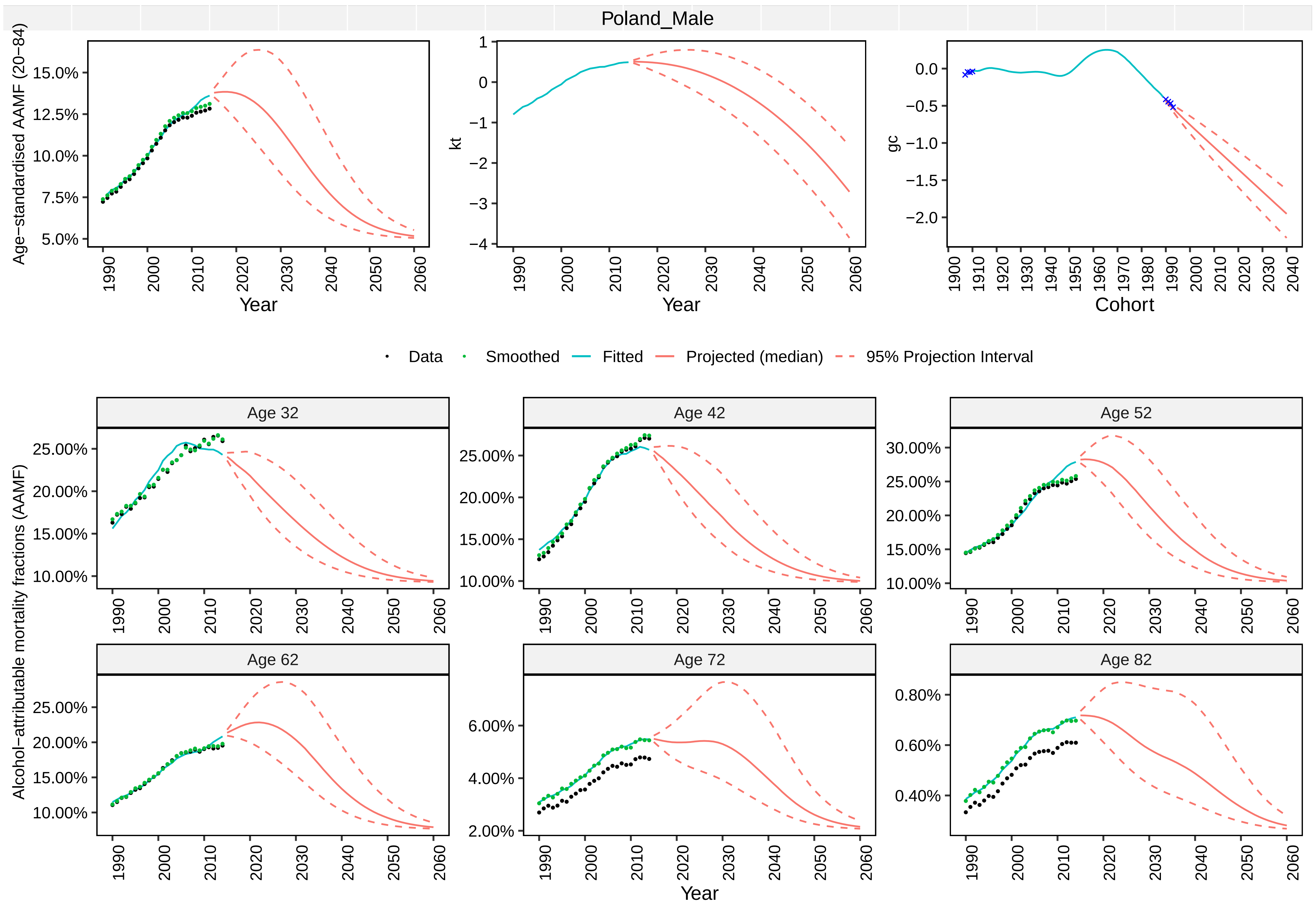
Poland_Male
Age−standardised AAMF (20−84)
Year
kt
gc
Cohort
Data
Smoothed
Fitted
Projected (median)
95% Projection Interval
Age 32
Age 42
Age 52
Age 62
Age 72
Age 82
Alcohol−attributable mortality fractions (AAMF)
Year

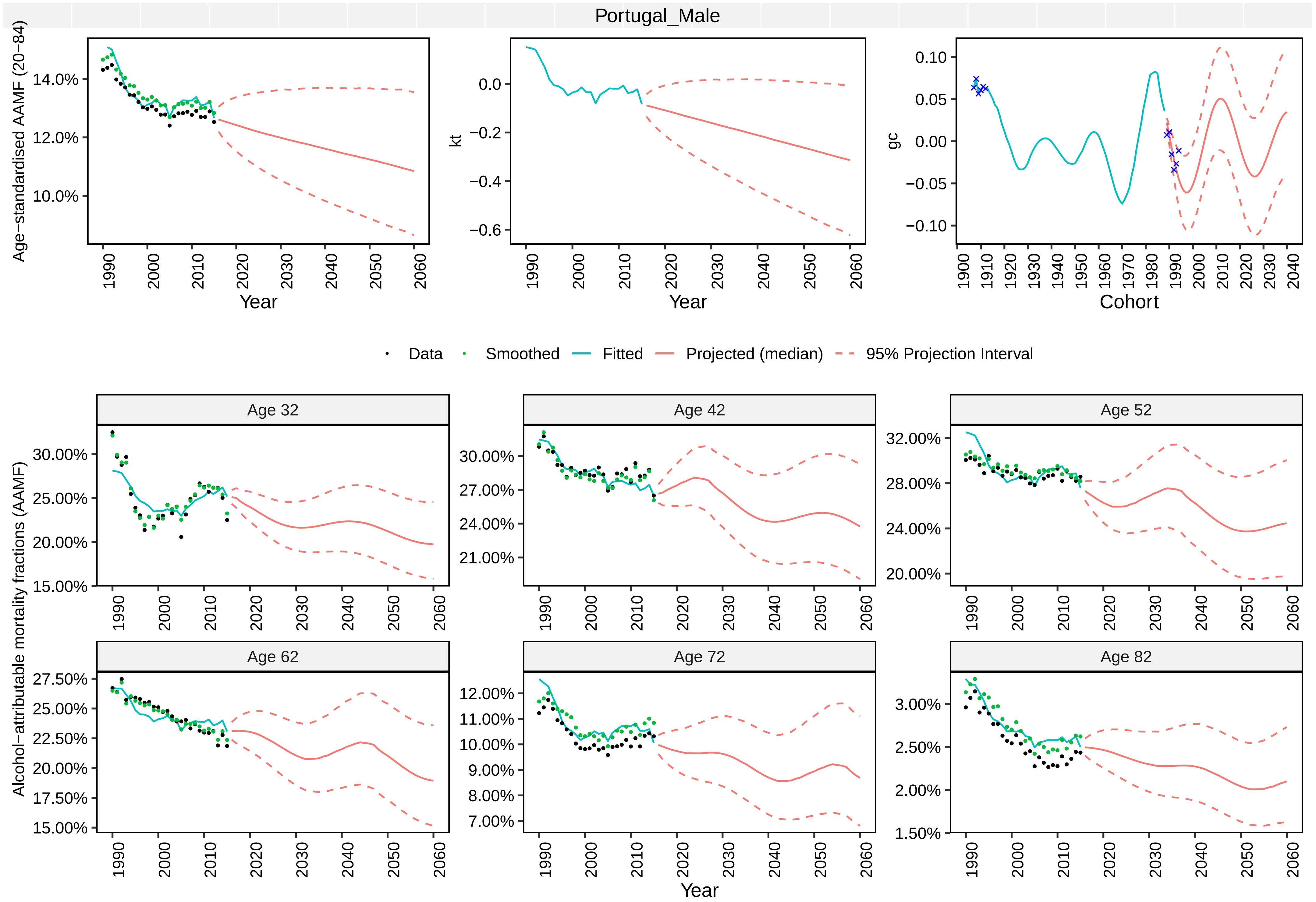

Portugal_Male
Age−standardised AAMF (20−84)
14.0%
12.0%
10.0%
Year
kt
0.0
−0.2
−0.4
−0.6
gc
0.10
0.05
0.00
−0.05
−0.10
Cohort
Data
Smoothed
Fitted
Projected (median)
95% Projection Interval
Age 32
Age 42
Age 52
Age 62
Age 72
Age 82
Alcohol−attributable mortality fractions (AAMF)
Year

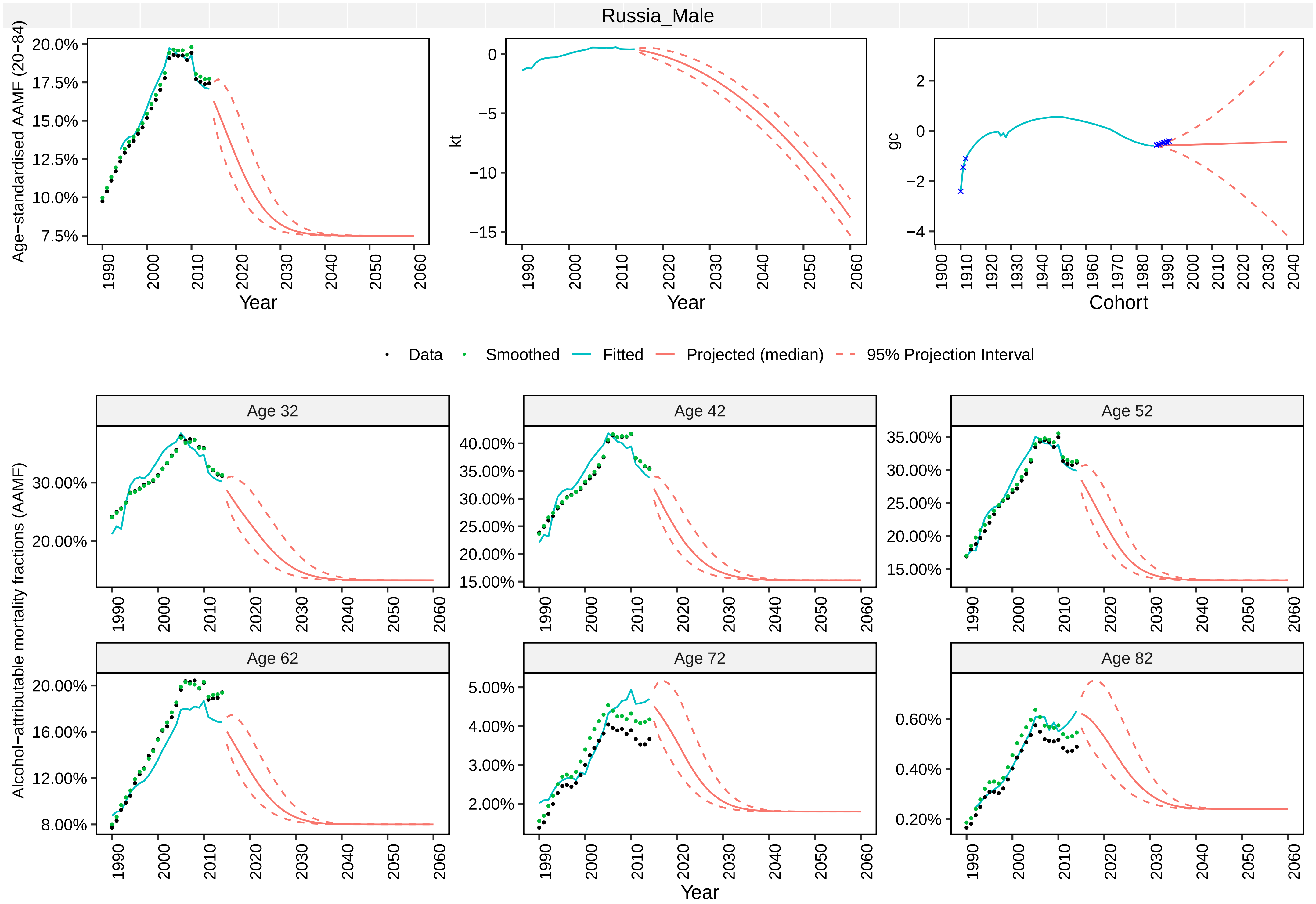

Russia_Male
Age−standardised AAMF (20−84)
Year
kt
gc
Cohort
Data
Smoothed
Fitted
Projected (median)
95% Projection Interval
Age 32
Age 42
Age 52
Age 62
Age 72
Age 82
Alcohol−attributable mortality fractions (AAMF)
Year

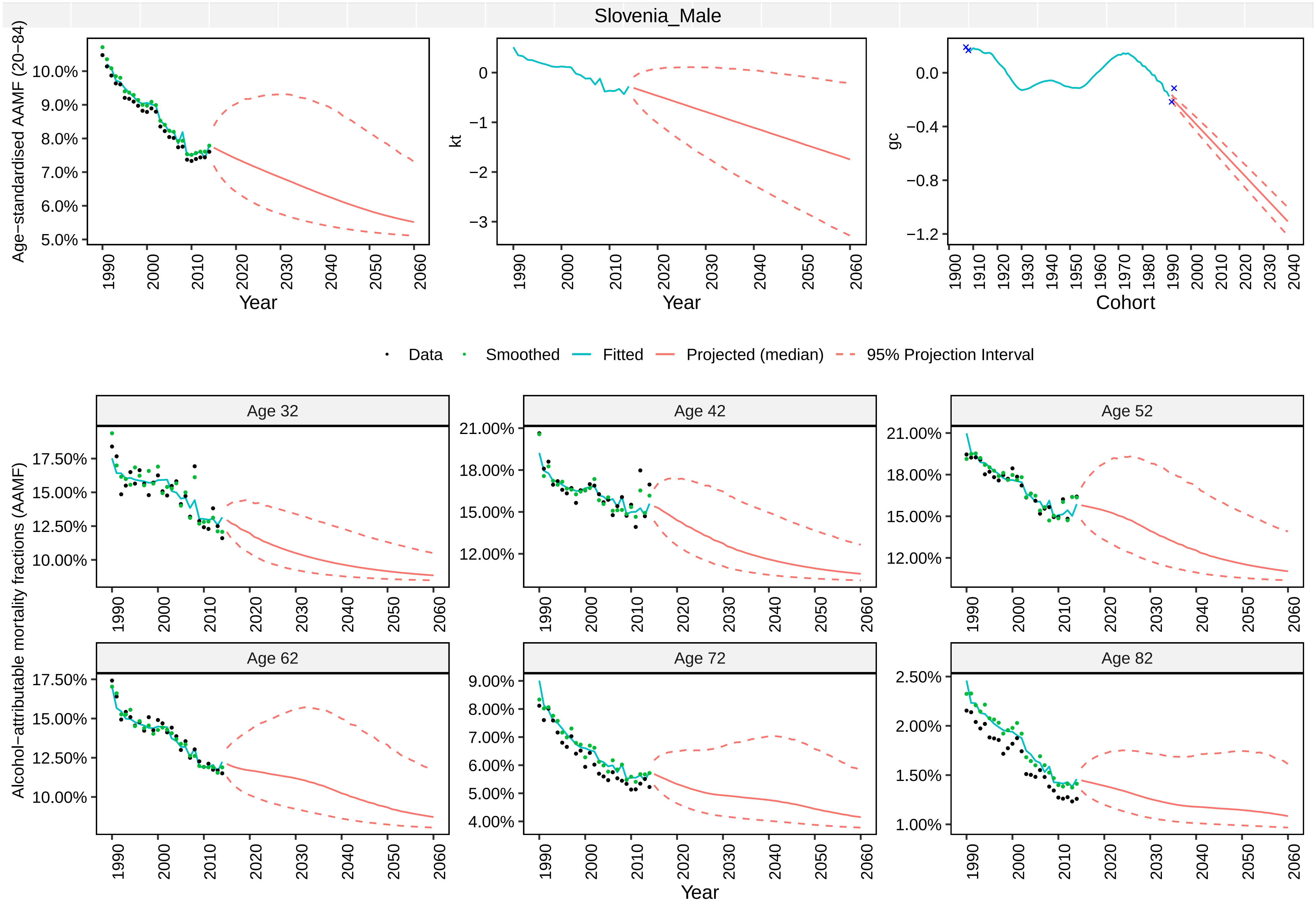

Slovenia_Male
Age−standardised AAMF (20−84)
Year
kt
gc
Cohort
Data
Smoothed
Fitted
Projected (median)
95% Projection Interval
Age 32
Age 42
Age 52
Age 62
Age 72
Age 82
Alcohol−attributable mortality fractions (AAMF)

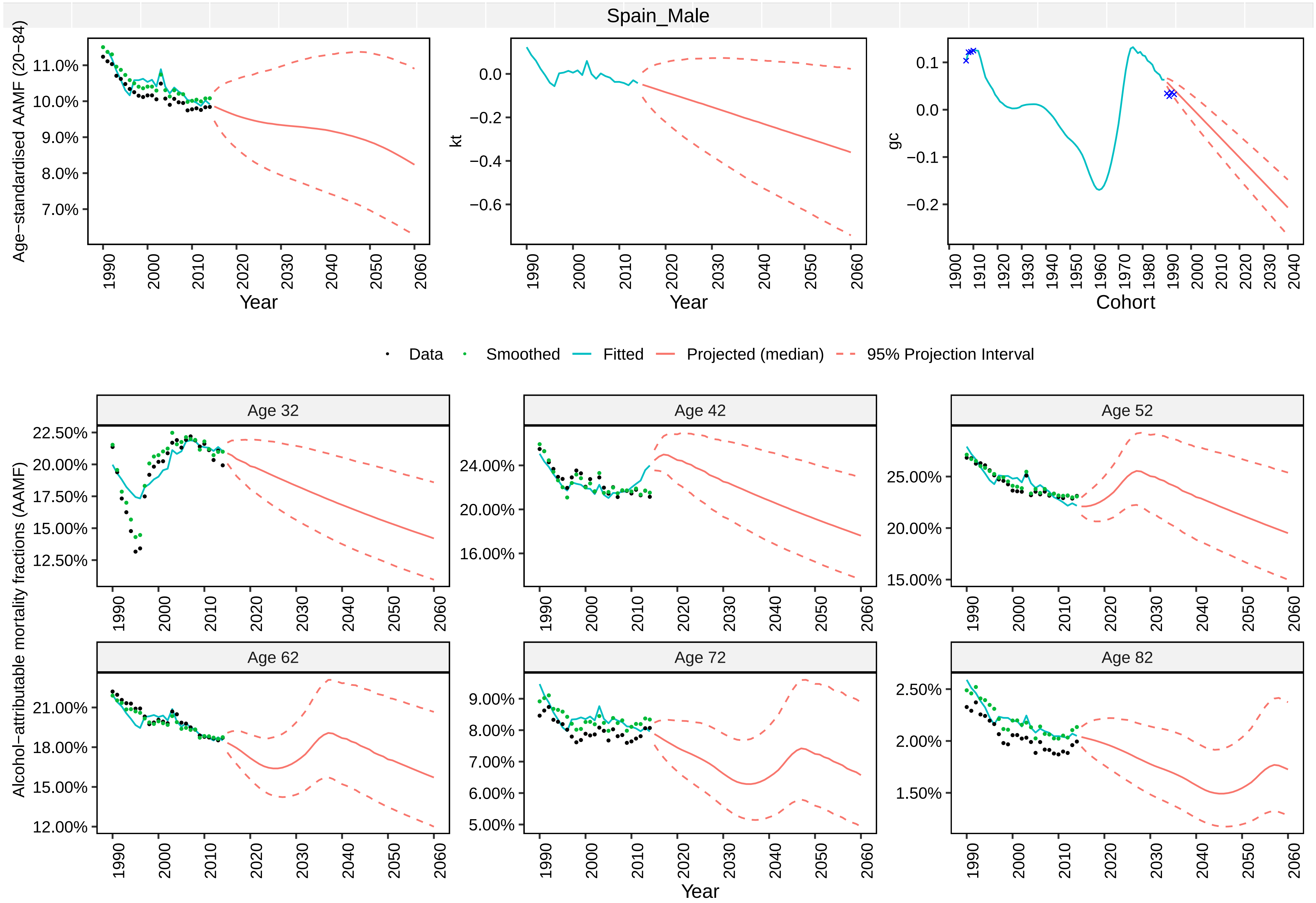

Spain_Male
Age−standardised AAMF (20−84)
11.0%
10.0%
9.0%
8.0%
7.0%
Year
kt
0.0
−0.2
−0.4
−0.6
gc
0.1
0.0
−0.1
−0.2
Cohort
Data
Smoothed
Fitted
Projected (median)
95% Projection Interval
Age 32
Age 42
Age 52
Age 62
Age 72
Age 82
Alcohol−attributable mortality fractions (AAMF)
Year

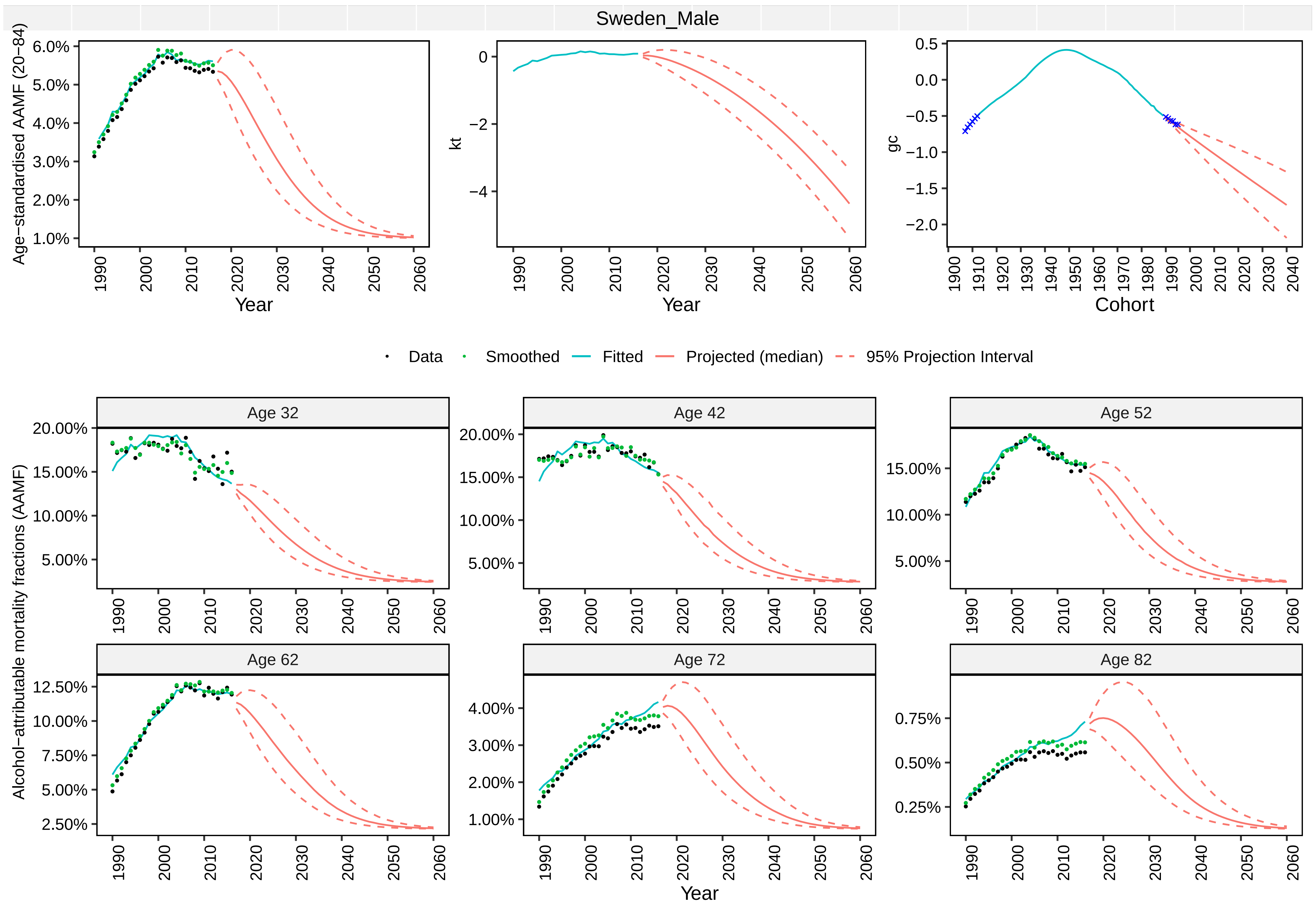

Sweden_Male
Age−standardised AAMF (20−84)
6.0%
5.0%
4.0%
3.0%
2.0%
1.0%
Year
kt
Year
gc
Cohort
Data
Smoothed
Fitted
Projected (median)
95% Projection Interval
Age 32
Age 42
Age 52
Age 62
Age 72
Age 82
Alcohol−attributable mortality fractions (AAMF)
Year

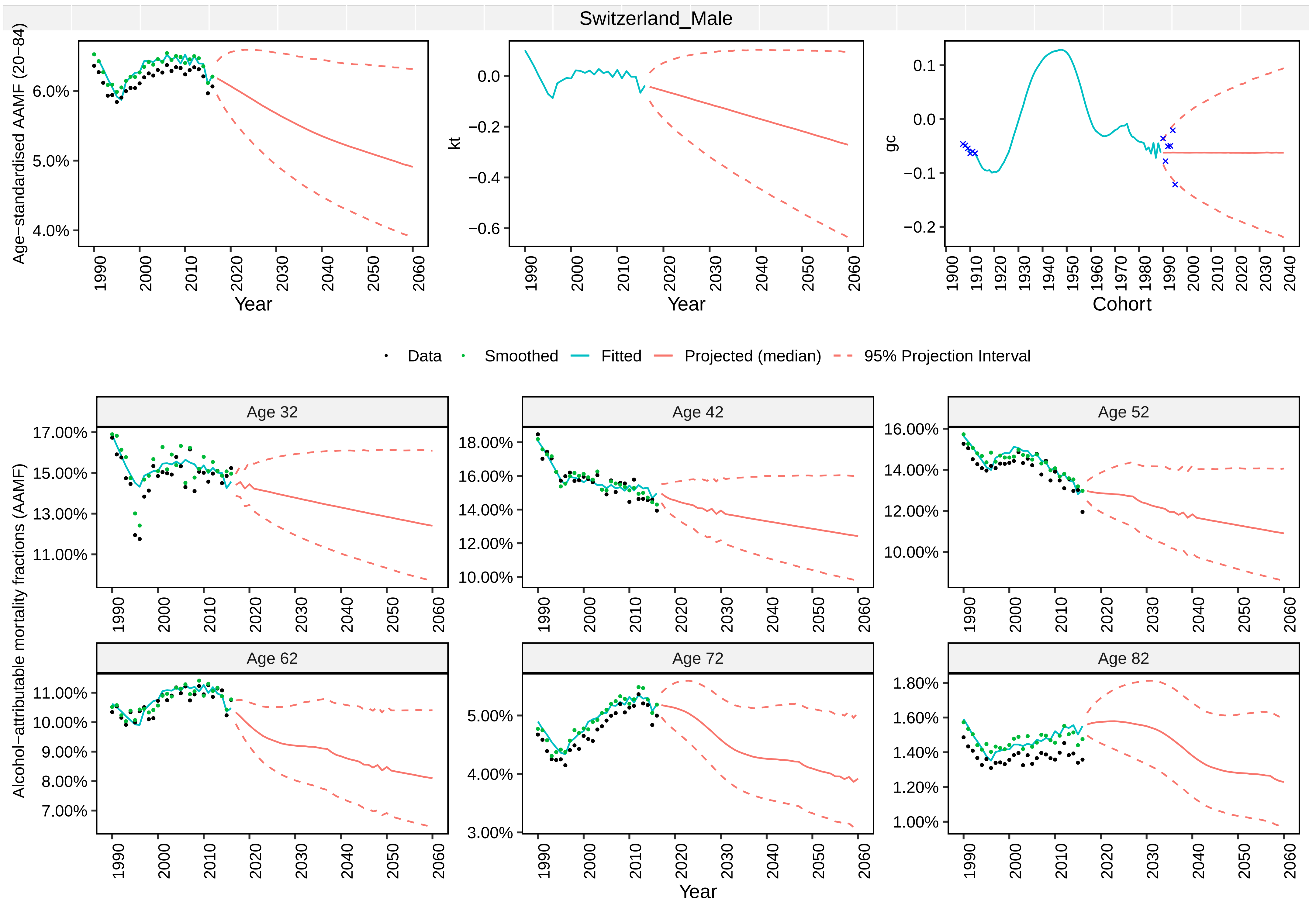

Switzerland_Male
Age−standardised AAMF (20−84)
6.0%
5.0%
4.0%
kt
0.0
−0.2
−0.4
−0.6
gc
0.1
0.0
−0.1
−0.2
Year
Cohort
Data
Smoothed
Fitted
Projected (median)
95% Projection Interval
Age 32
Age 42
Age 52
Age 62
Age 72
Age 82
Alcohol−attributable mortality fractions (AAMF)

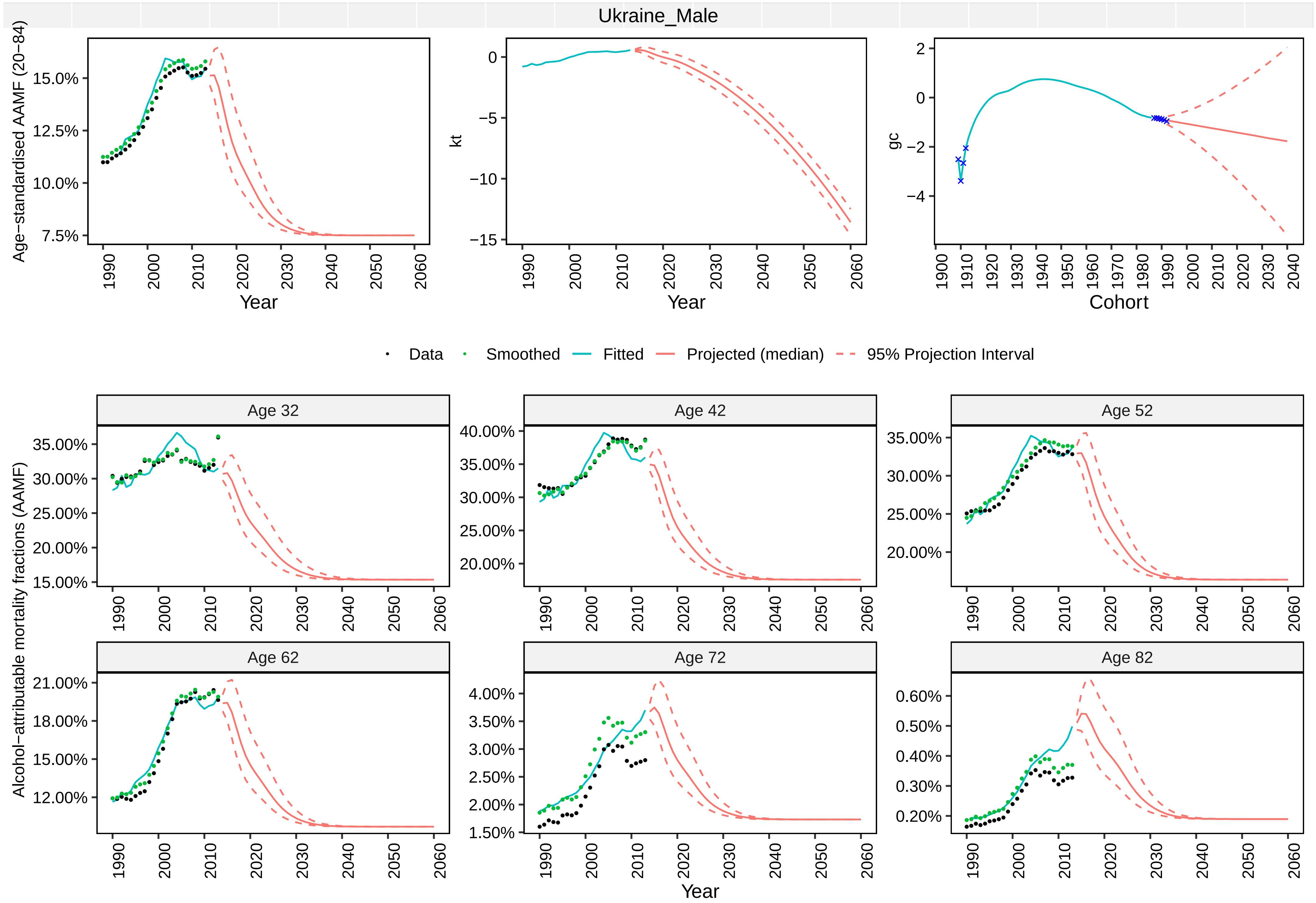

Ukraine_Male
Age−standardised AAMF (20−84)
15.0%
12.5%
10.0%
7.5%
Year
kt
0
−5
−10
−15
gc
2
0
−2
−4
Cohort
1990
2000
2010
2020
2030
2040
2050
2060
1900
1910
1920
1930
1940
1950
1960
1970
1980
Data
Smoothed
Fitted
Projected (median)
95% Projection Interval
Age 32
Age 42
Age 52
Age 62
Age 72
Age 82
Alcohol−attributable mortality fractions (AAMF)
35.00%
30.00%
25.00%
20.00%
15.00%
40.00%
21.00%
18.00%
15.00%
12.00%
4.00%
3.50%
3.00%
2.50%
2.00%
1.50%
0.60%
0.50%
0.40%
0.30%
0.20%

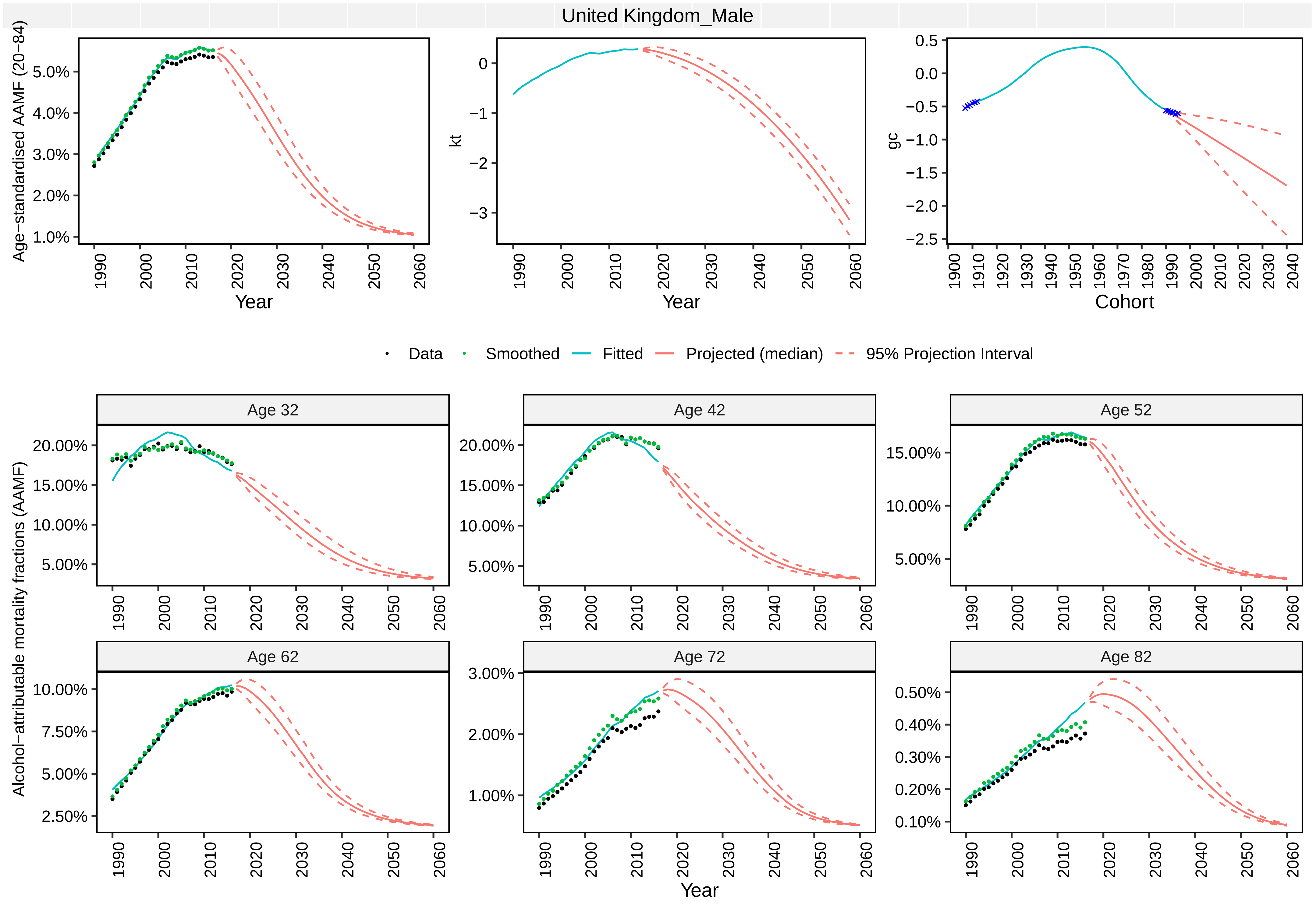

United Kingdom_Male
Age−standardised AAMF (20−84)
Year
kt
gc
Cohort
Data
Smoothed
Fitted
Projected (median)
95% Projection Interval
Age 32
Age 42
Age 52
Age 62
Age 72
Age 82
Alcohol−attributable mortality fractions (AAMF)
Year
