# Supplementary material for: Past and Future Alcohol-Attributable Mortality in Europe
Source: Int J Environ Res Public Health. 2020 Dec 3;17(23):9024. doi: 10.3390/ijerph17239024 (PMC7730378; doi:10.3390/ijerph17239024)
Supplement: Supplementary file 1 [file ijerph-17-09024-s001.zip › Supplementary file 3. Detailed_projection_outcomes_women.pdf]

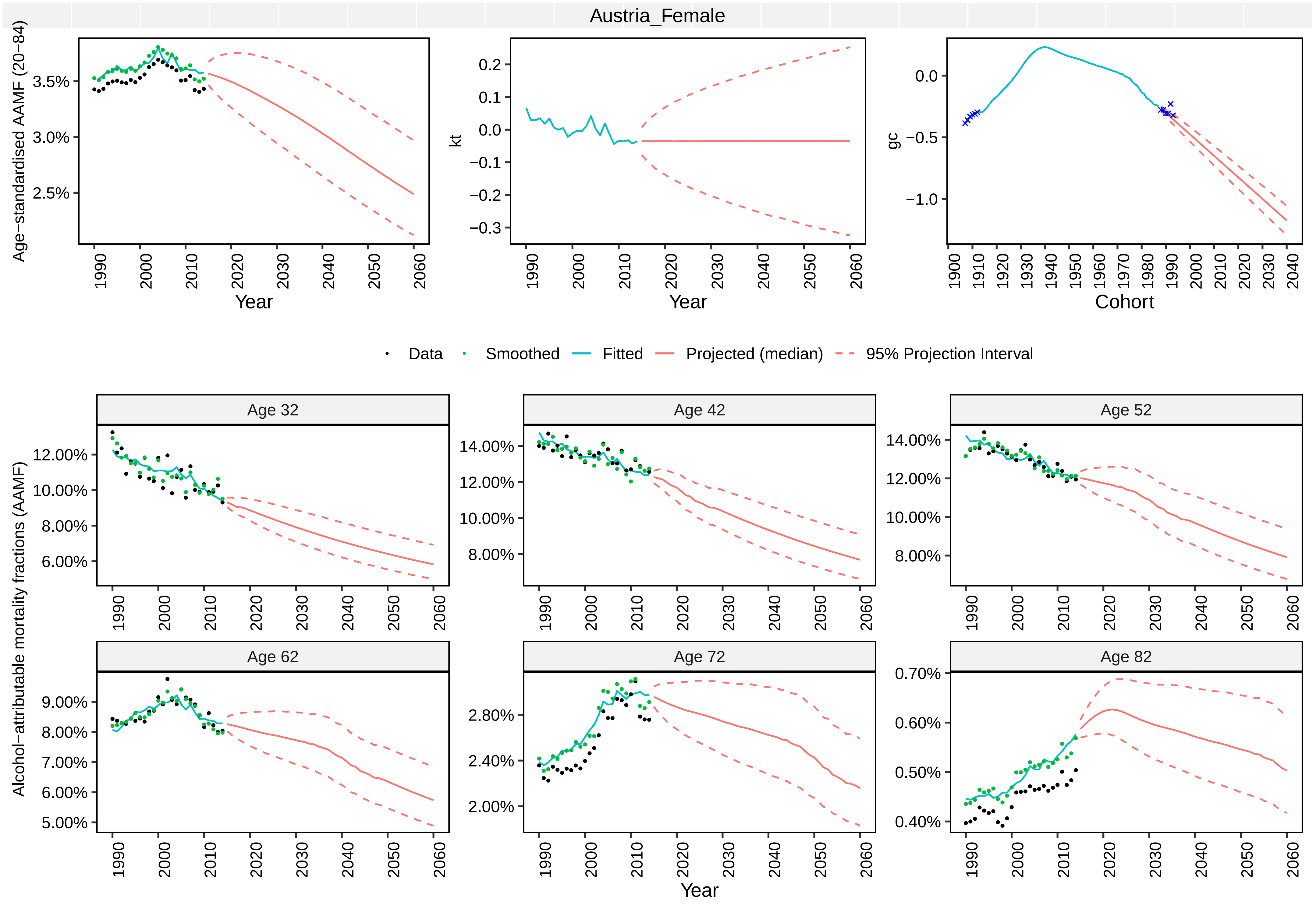
Austria_Female
Age−standardised AAMF (20−84)
3.5%
3.0%
2.5%
Year
kt
Year
gc
Cohort
Data
Smoothed
Fitted
Projected (median)
95% Projection Interval
Age 32
Age 42
Age 52
Age 62
Age 72
Age 82
Alcohol−attributable mortality fractions (AAMF)
Year

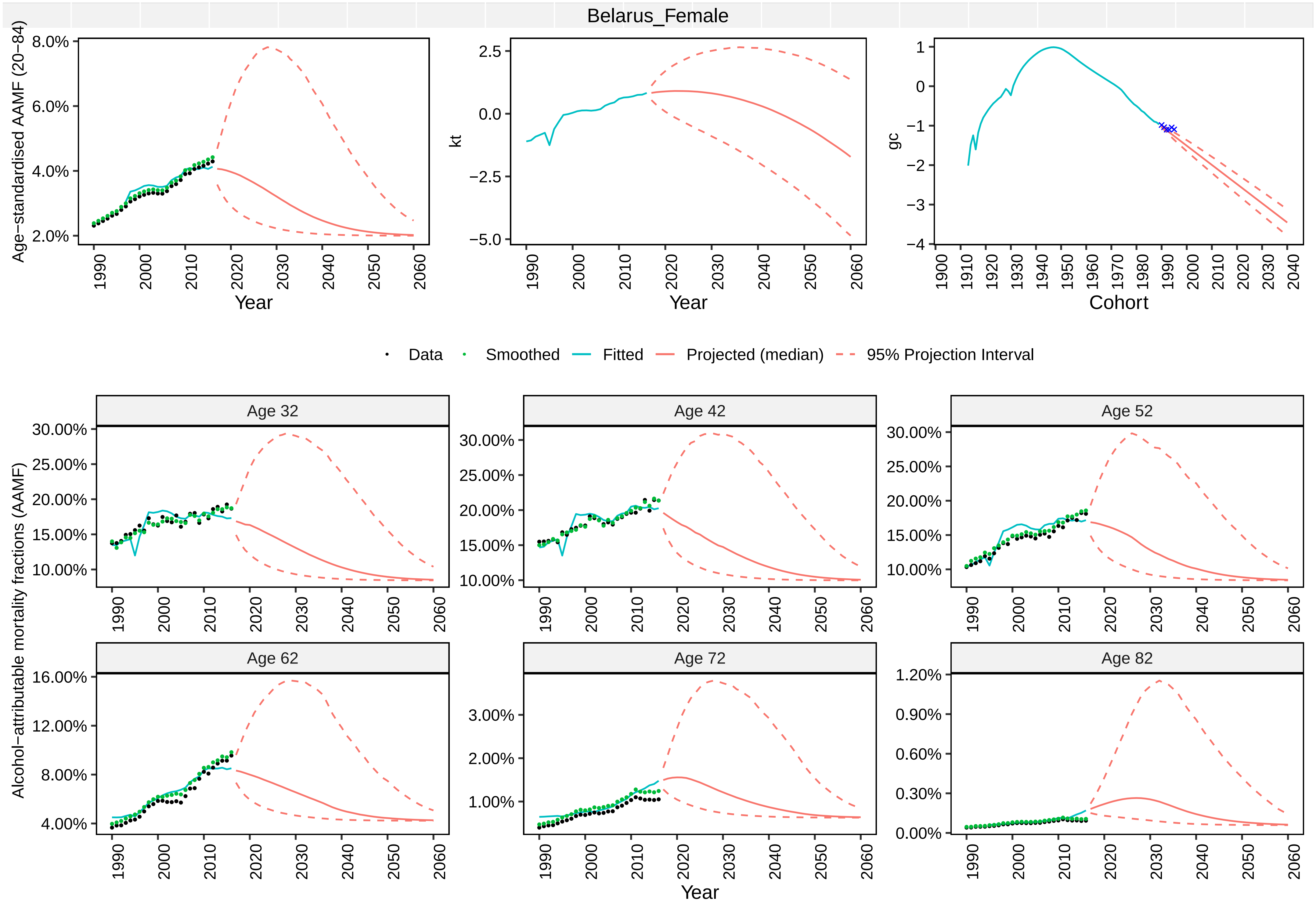
Belarus_Female
Age−standardised AAMF (20−84)
Year
kt
gc
Cohort
Data
Smoothed
Fitted
Projected (median)
95% Projection Interval
Age 32
Age 42
Age 52
Age 62
Age 72
Age 82
Alcohol−attributable mortality fractions (AAMF)

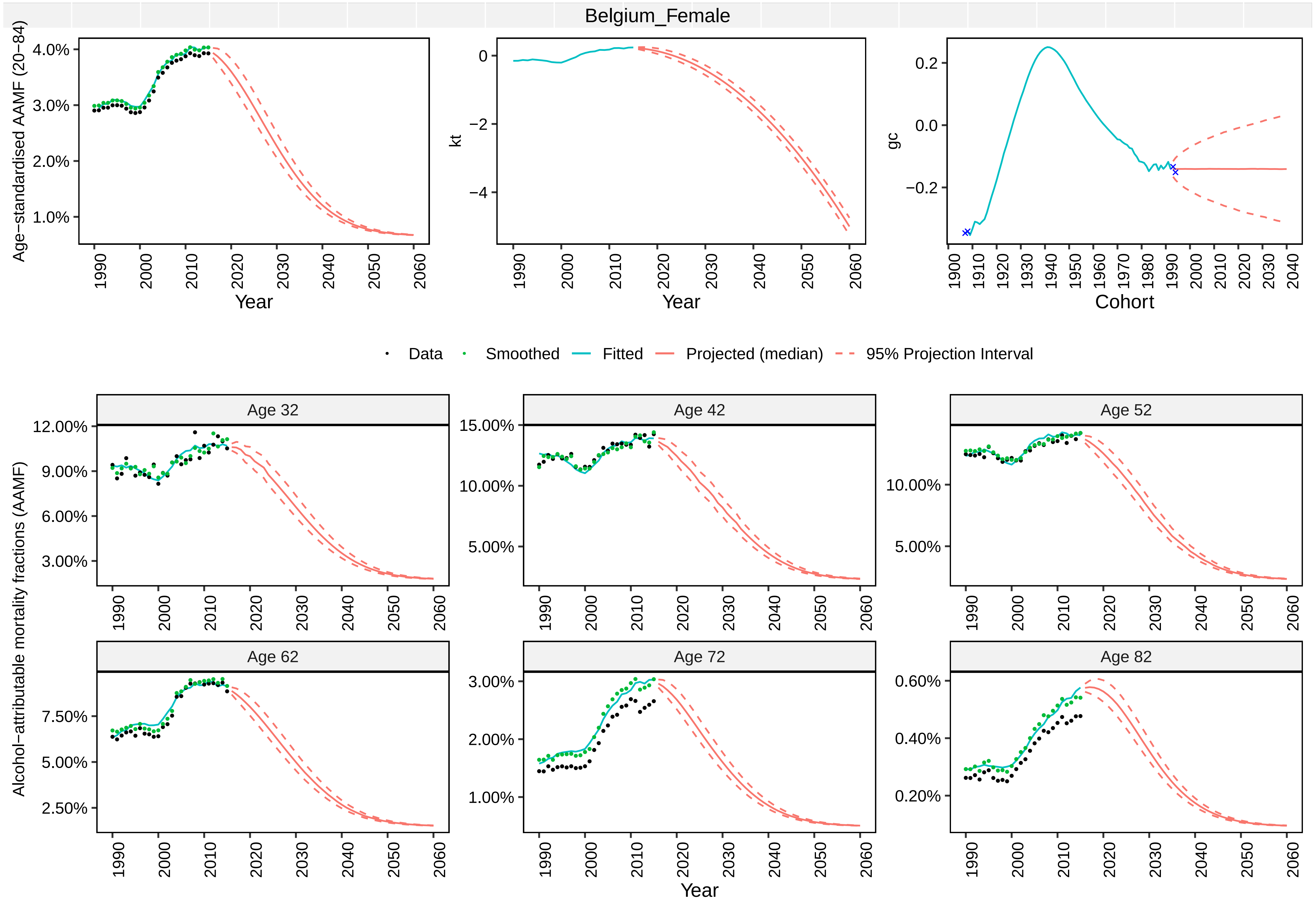

Belgium_Female
Age−standardised AAMF (20−84)
4.0%
3.0%
2.0%
1.0%
Year
kt
0
−2
−4
gc
0.2
0.0
−0.2
Cohort
1900
1910
1920
1930
1940
1950
1960
1970
1980
1990
2000
2010
2020
2030
2040
2050
2060
Data
Smoothed
Fitted
Projected (median)
95% Projection Interval
Alcohol−attributable mortality fractions (AAMF)
Age 32
Age 42
Age 52
Age 62
Age 72
Age 82
12.00%
9.00%
6.00%
3.00%
15.00%
10.00%
5.00%
7.50%
5.00%
2.50%
3.00%
2.00%
1.00%
0.60%
0.40%
0.20%

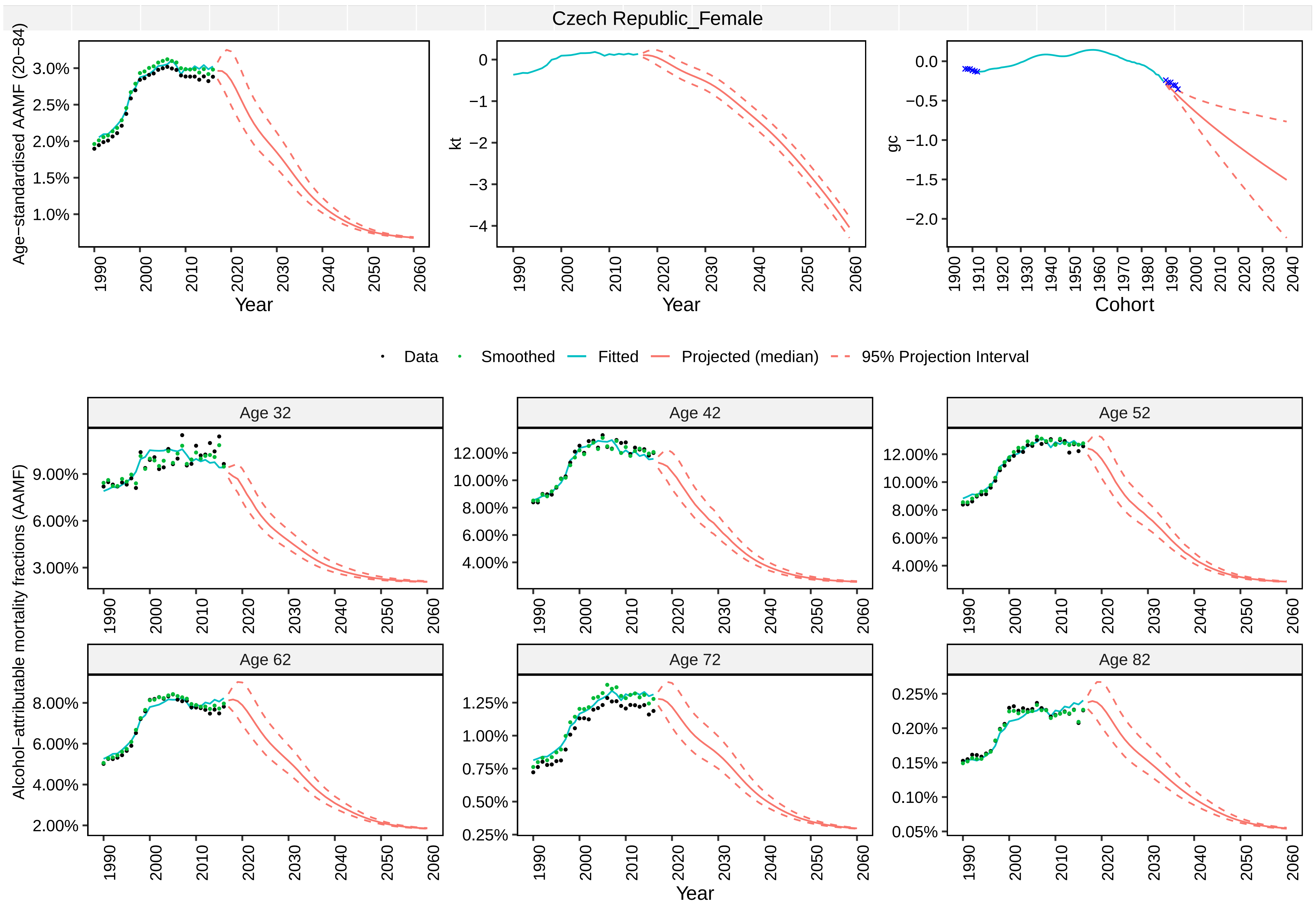

Czech Republic_Female
Age−standardised AAMF (20−84)
Year
kt
gc
Cohort
Data
Smoothed
Fitted
Projected (median)
95% Projection Interval
Age 32
Age 42
Age 52
Age 62
Age 72
Age 82
Alcohol−attributable mortality fractions (AAMF)

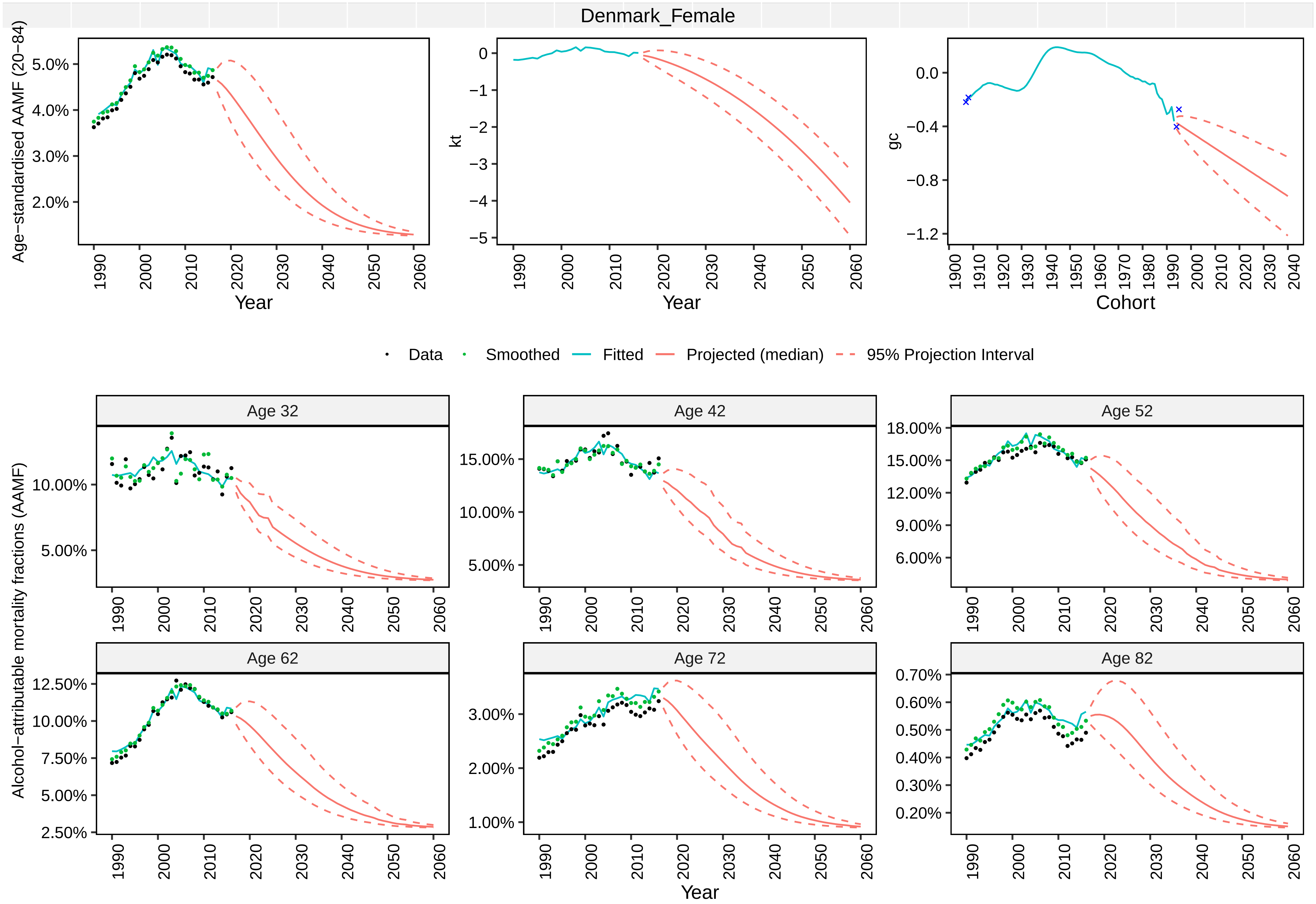
Denmark_Female
Age−standardised AAMF (20−84)
kt
gc
Year
Year
Cohort
Data
Smoothed
Fitted
Projected (median)
95% Projection Interval
Age 32
Age 42
Age 52
Age 62
Age 72
Age 82
Alcohol−attributable mortality fractions (AAMF)
Year

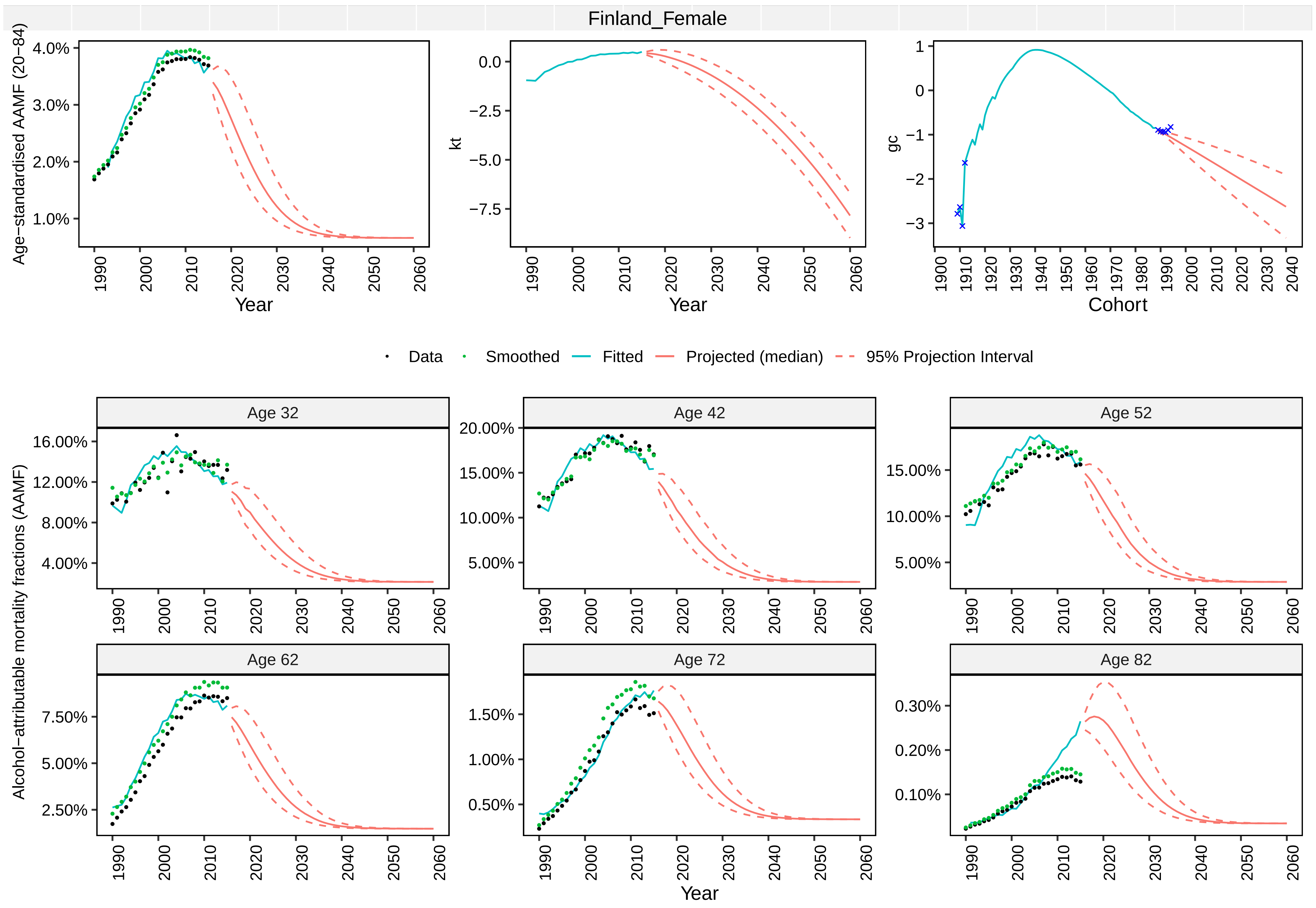

Finland_Female
Age−standardised AAMF (20−84)
4.0%
3.0%
2.0%
1.0%
Year
kt
0.0
−2.5
−5.0
−7.5
gc
Cohort
Data
Smoothed
Fitted
Projected (median)
95% Projection Interval
Age 32
Age 42
Age 52
Age 62
Age 72
Age 82
Alcohol−attributable mortality fractions (AAMF)
Year

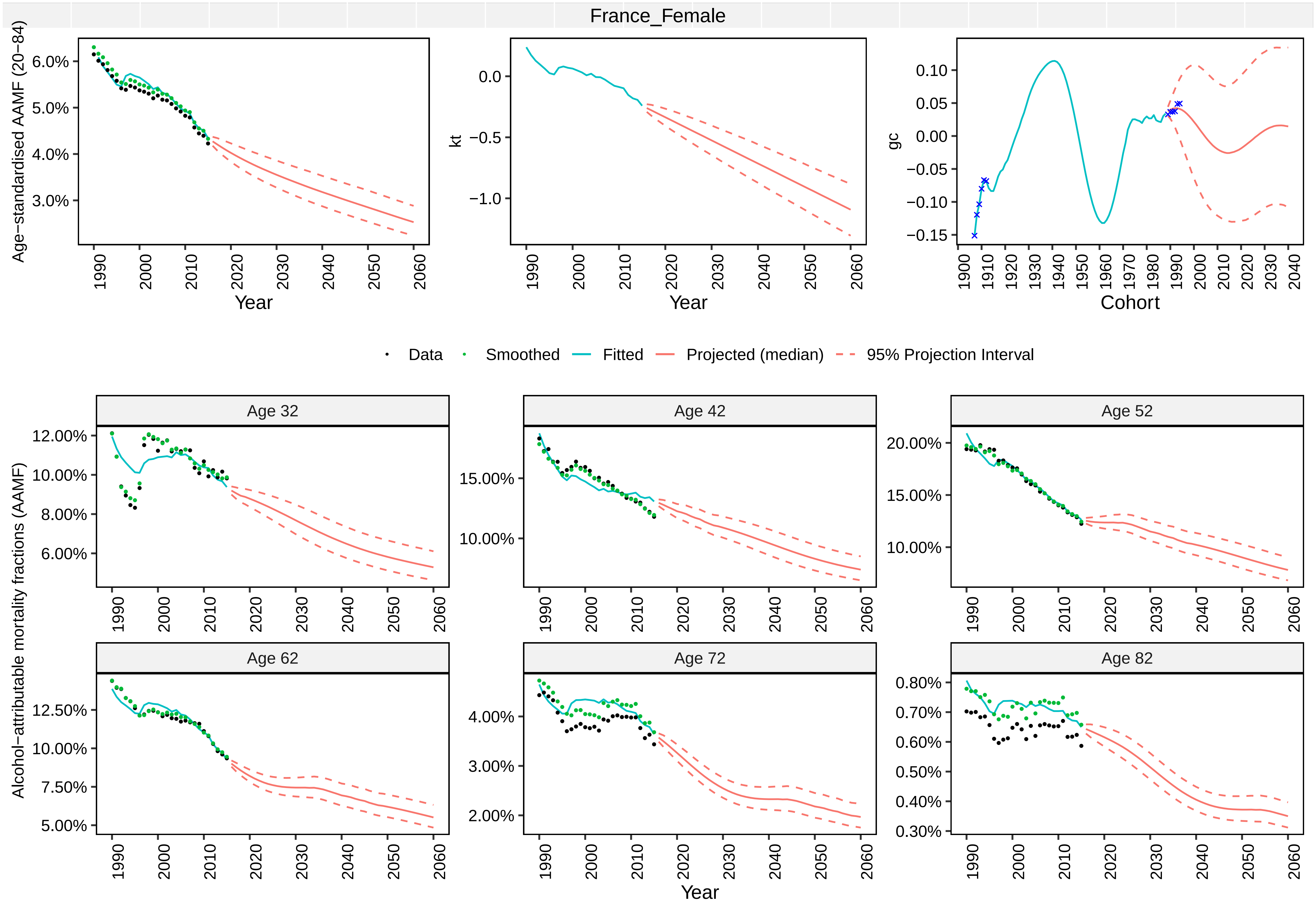
France_Female
Age−standardised AAMF (20−84)
6.0%
5.0%
4.0%
3.0%
Year
kt
0.0
−0.5
−1.0
gc
0.10
0.05
0.00
−0.05
−0.10
−0.15
Cohort
Data
Smoothed
Fitted
Projected (median)
95% Projection Interval
Alcohol−attributable mortality fractions (AAMF)
Age 32
Age 42
Age 52
Age 62
Age 72
Age 82
Year

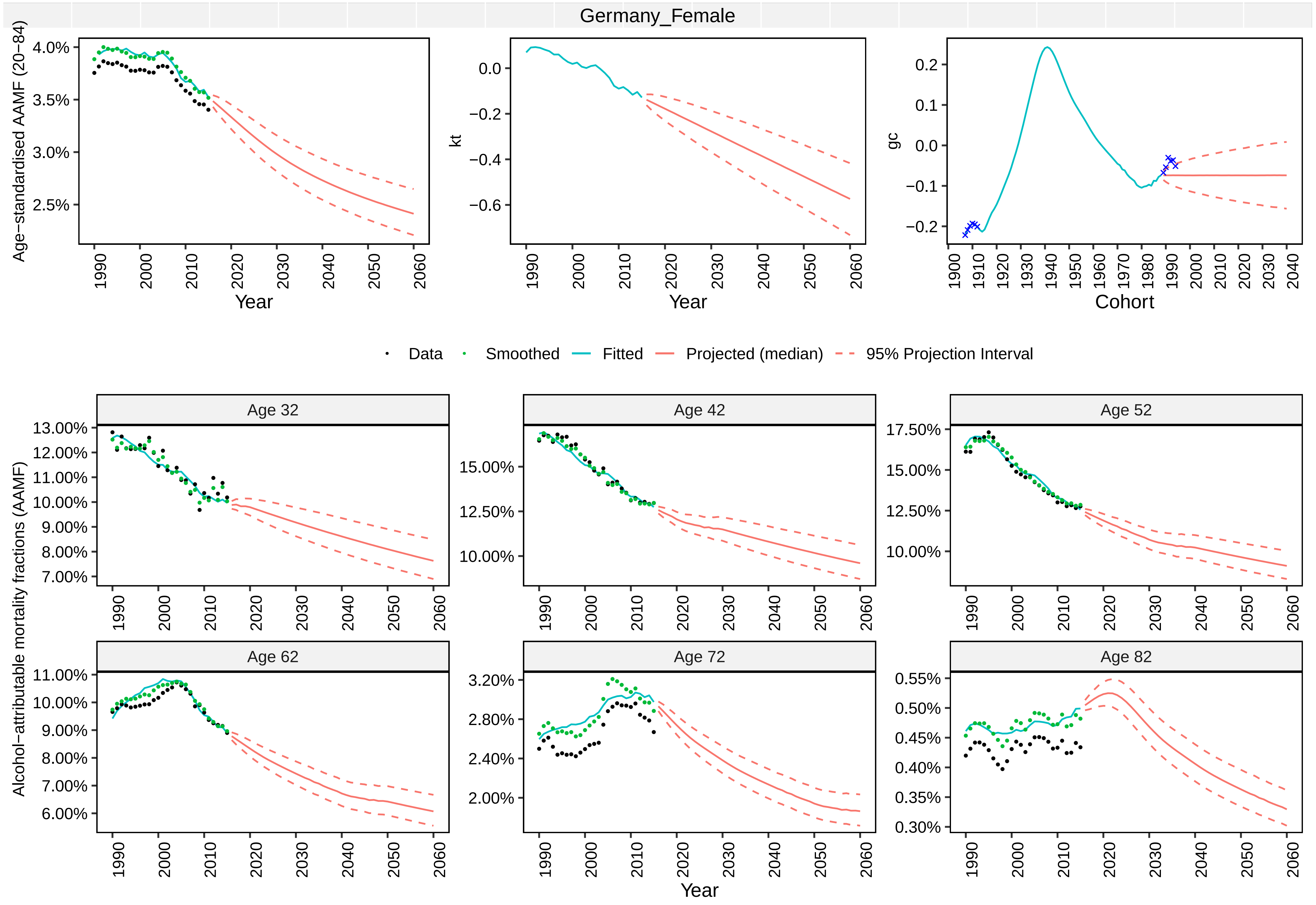

Germany_Female
Age−standardised AAMF (20−84)
4.0%
3.5%
3.0%
2.5%
Year
kt
0.0
−0.2
−0.4
−0.6
gc
0.2
0.1
0.0
−0.1
−0.2
Cohort
Data
Smoothed
Fitted
Projected (median)
95% Projection Interval
Alcohol−attributable mortality fractions (AAMF)
Age 32
Age 42
Age 52
Age 62
Age 72
Age 82
13.00%
12.00%
11.00%
10.00%
9.00%
8.00%
7.00%
15.00%
12.50%
10.00%
17.50%
6.00%
3.20%
2.80%
2.40%
2.00%
0.55%
0.50%
0.45%
0.40%
0.35%
0.30%
1990
2000
2010
2020
2030
2040
2050
2060
Year

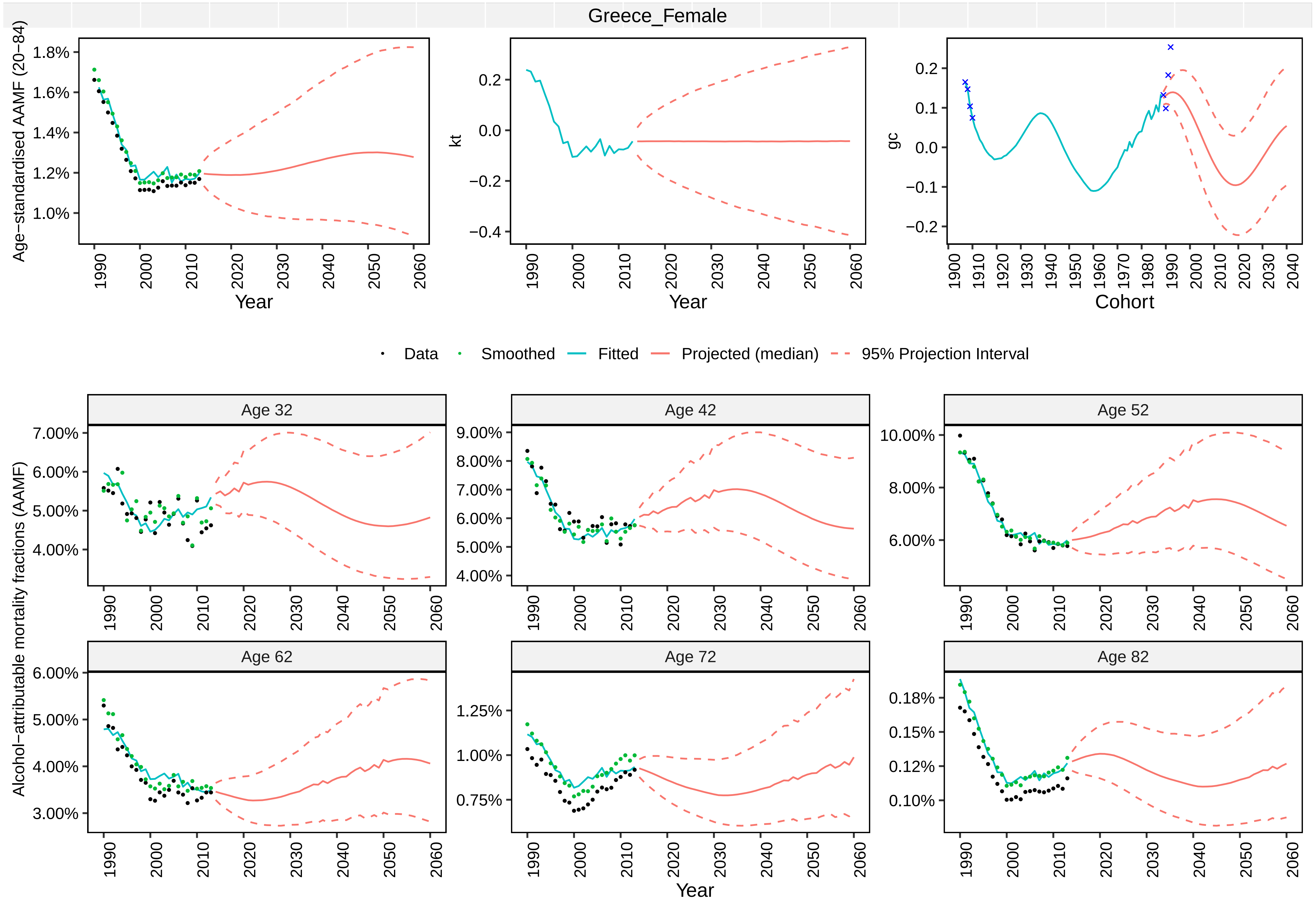
Greece_Female
Age−standardised AAMF (20−84)
1.8%
1.6%
1.4%
1.2%
1.0%
Year
kt
0.2
0.0
−0.2
−0.4
Year
gc
0.2
0.1
0.0
−0.1
−0.2
Cohort
Data
Smoothed
Fitted
Projected (median)
95% Projection Interval
Age 32
Age 42
Age 52
Age 62
Age 72
Age 82
Alcohol−attributable mortality fractions (AAMF)
Year

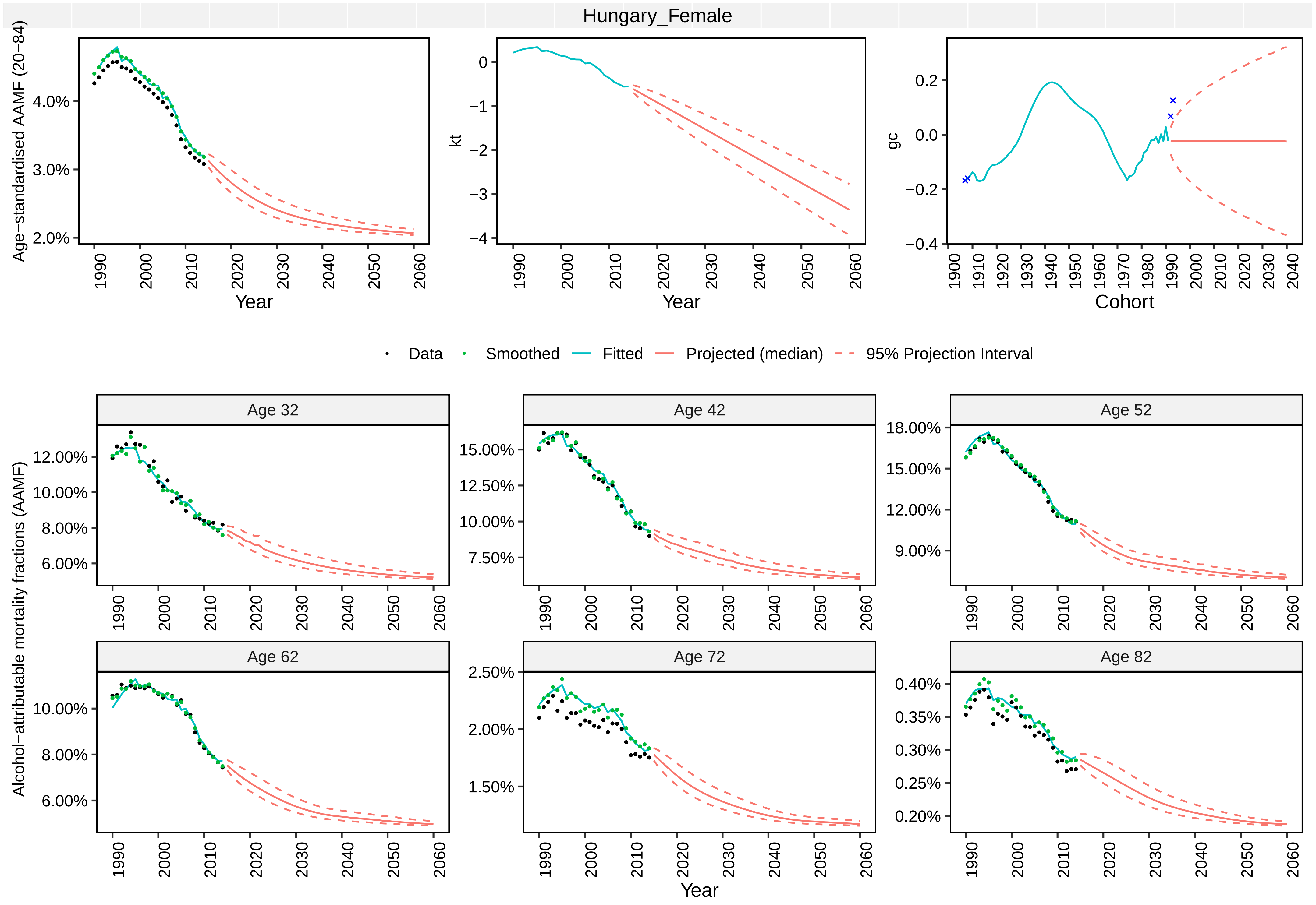
Hungary_Female
Age−standardised AAMF (20−84)
kt
gc
Year
Cohort
Data
Smoothed
Fitted
Projected (median)
95% Projection Interval
Age 32
Age 42
Age 52
Age 62
Age 72
Age 82
Alcohol−attributable mortality fractions (AAMF)
Year

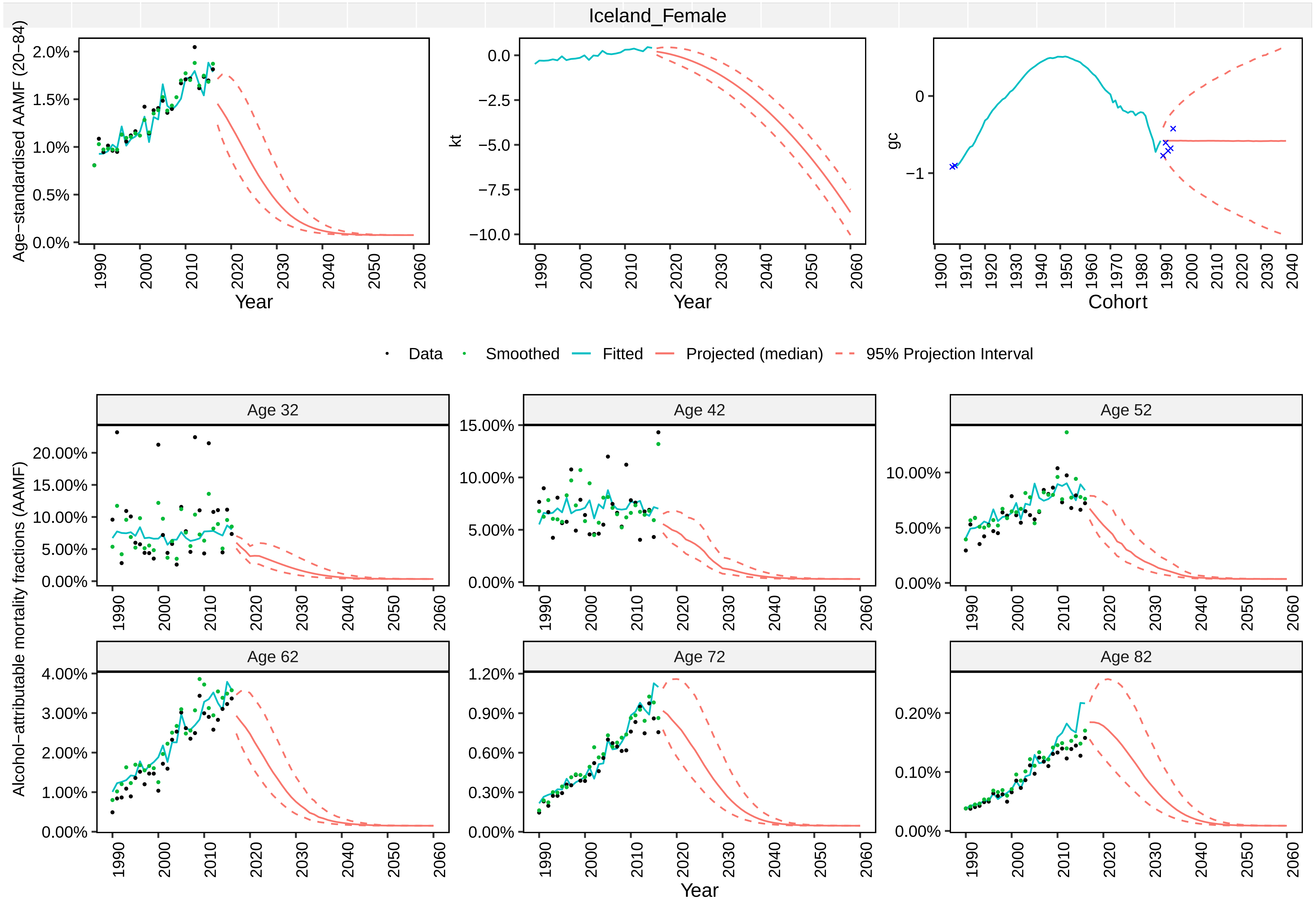
Iceland_Female
Age−standardised AAMF (20−84)
Year
kt
gc
Cohort
Data
Smoothed
Fitted
Projected (median)
95% Projection Interval
Age 32
Age 42
Age 52
Age 62
Age 72
Age 82
Alcohol−attributable mortality fractions (AAMF)

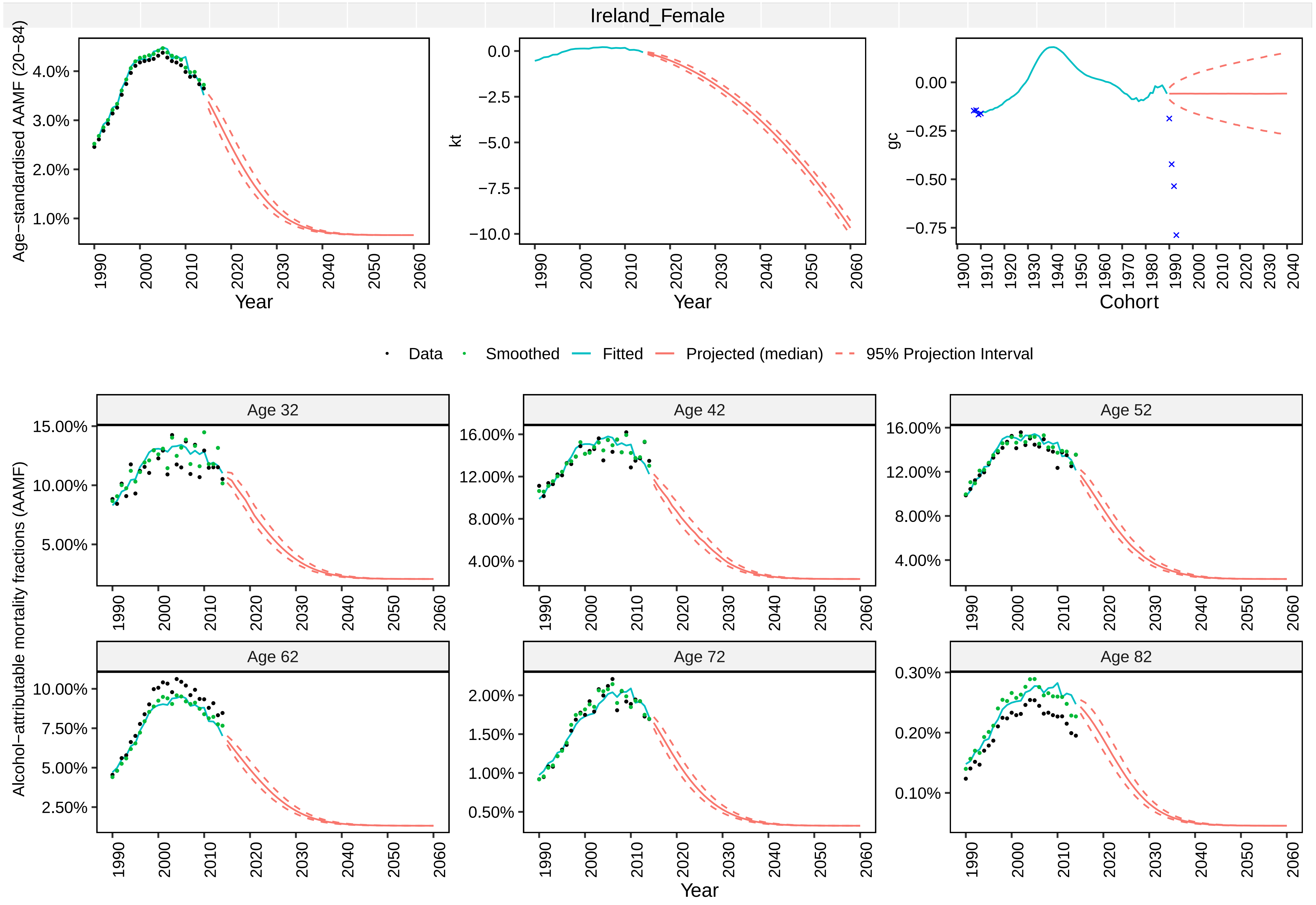

Ireland_Female
Age−standardised AAMF (20−84)
Year
kt
Year
gc
Cohort
Data
Smoothed
Fitted
Projected (median)
95% Projection Interval
Age 32
Age 42
Age 52
Age 62
Age 72
Age 82
Alcohol−attributable mortality fractions (AAMF)
Year

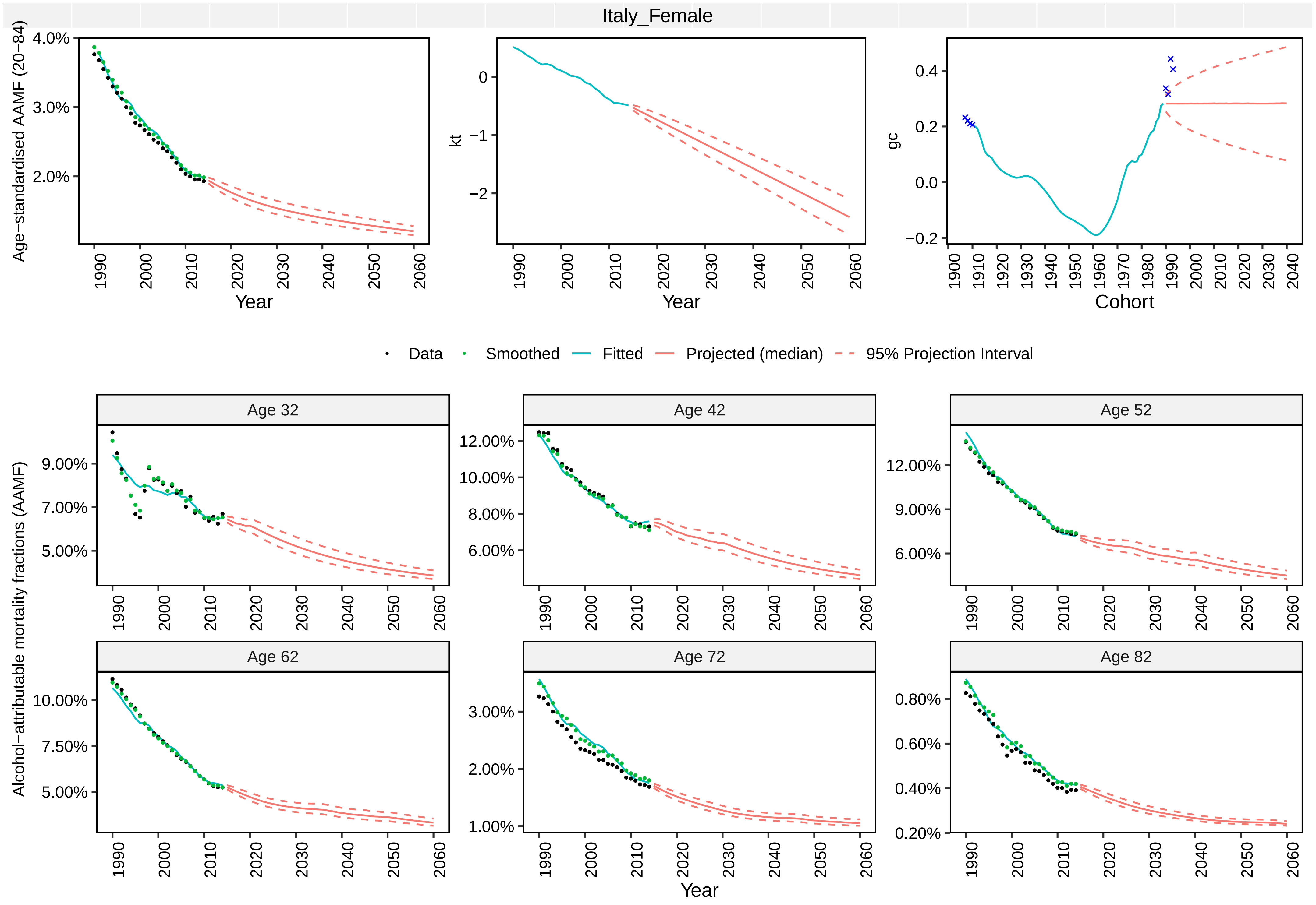
Italy_Female
Age−standardised AAMF (20−84)
4.0%
3.0%
2.0%
Year
kt
Year
gc
Cohort
Data
Smoothed
Fitted
Projected (median)
95% Projection Interval
Age 32
Age 42
Age 52
Age 62
Age 72
Age 82
Alcohol−attributable mortality fractions (AAMF)
Year

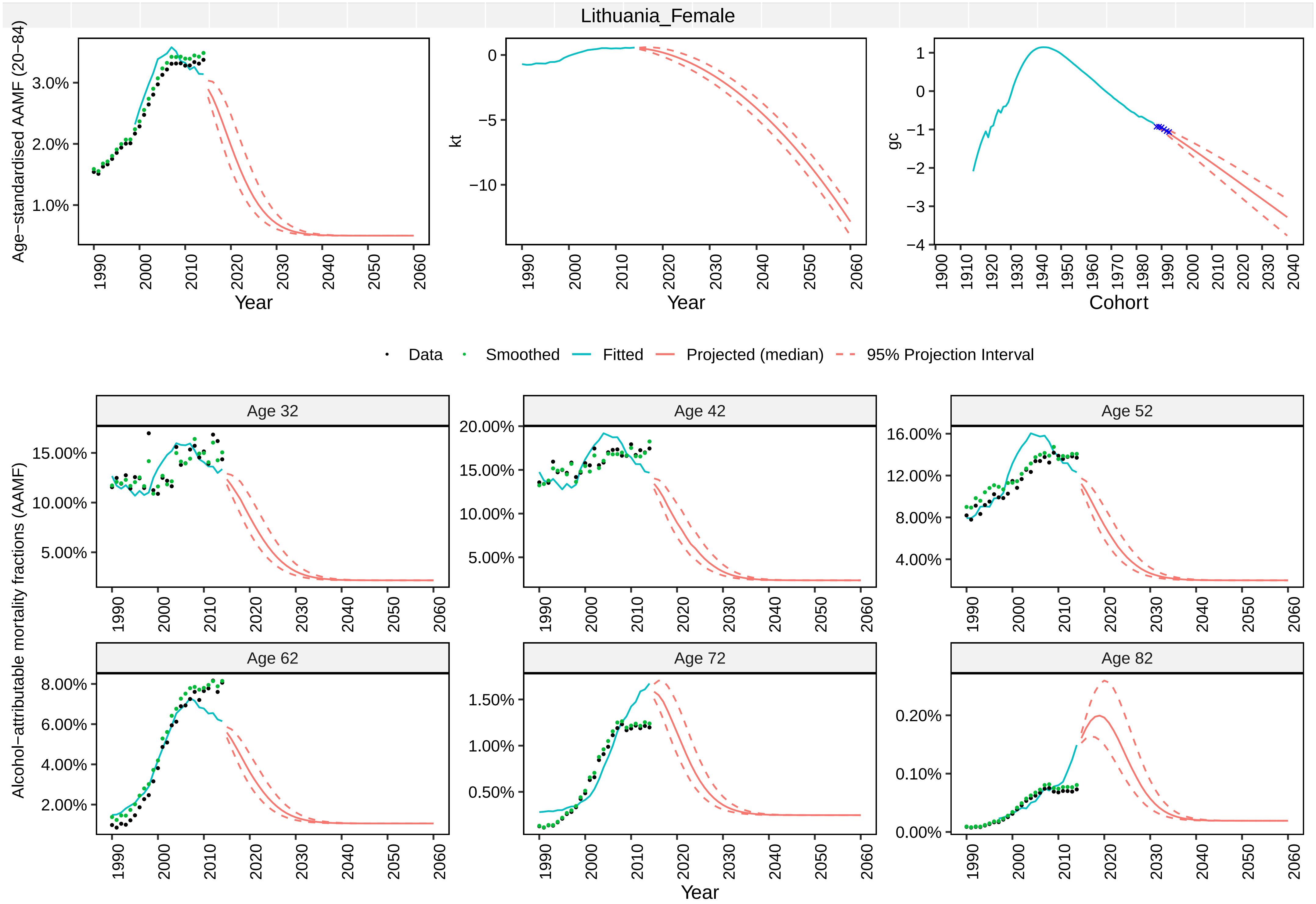

Lithuania_Female
Age−standardised AAMF (20−84)
3.0%
2.0%
1.0%
Year
kt
0
−5
−10
gc
1
0
−1
−2
−3
−4
Cohort
Data
Smoothed
Fitted
Projected (median)
95% Projection Interval
Age 32
Age 42
Age 52
Age 62
Age 72
Age 82
Alcohol−attributable mortality fractions (AAMF)
15.00%
10.00%
5.00%
20.00%
16.00%
12.00%
8.00%
4.00%
8.00%
6.00%
4.00%
2.00%
1.50%
1.00%
0.50%
0.20%
0.10%
0.00%
1990
2000
2010
2020
2030
2040
2050
2060
Year

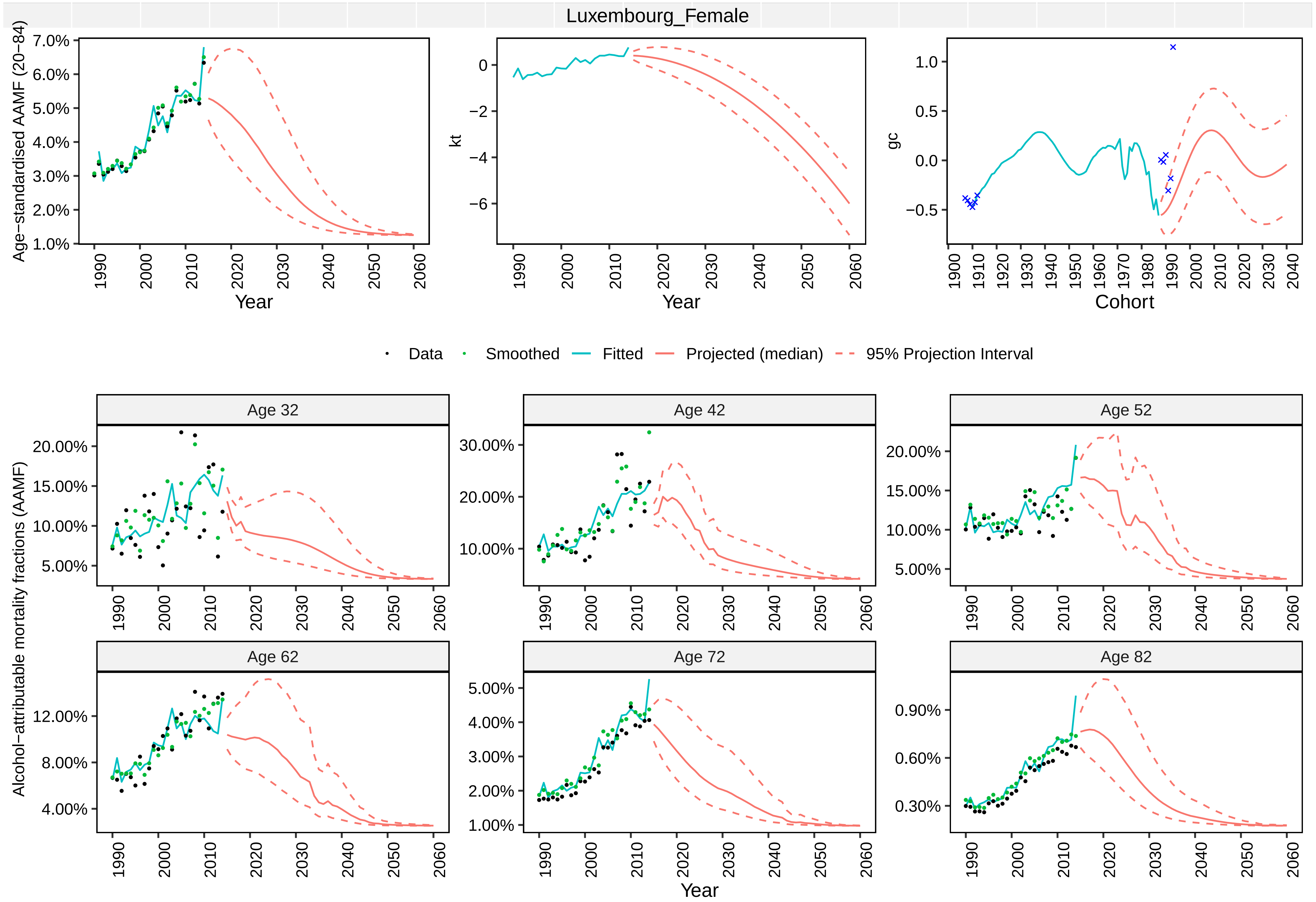
Luxembourg_Female
Age−standardised AAMF (20−84)
Year
kt
gc
Cohort
Data
Smoothed
Fitted
Projected (median)
95% Projection Interval
Age 32
Age 42
Age 52
Age 62
Age 72
Age 82
Alcohol−attributable mortality fractions (AAMF)

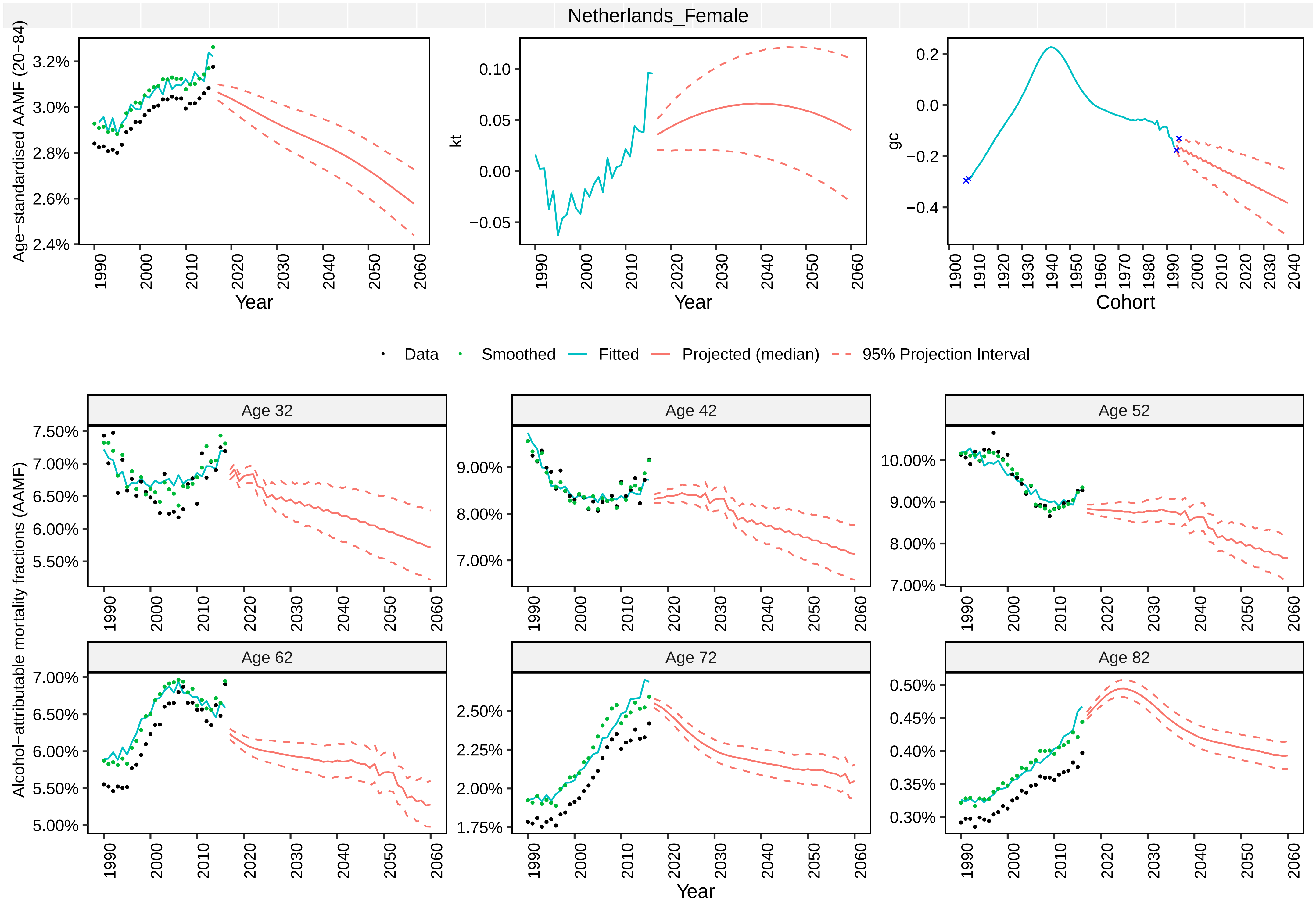

Netherlands_Female
Age−standardised AAMF (20−84)
3.2%
3.0%
2.8%
2.6%
2.4%
Year
kt
0.10
0.05
0.00
−0.05
gc
0.2
0.0
−0.2
−0.4
Cohort
Data
Smoothed
Fitted
Projected (median)
95% Projection Interval
Age 32
Age 42
Age 52
Age 62
Age 72
Age 82
Alcohol−attributable mortality fractions (AAMF)
Year

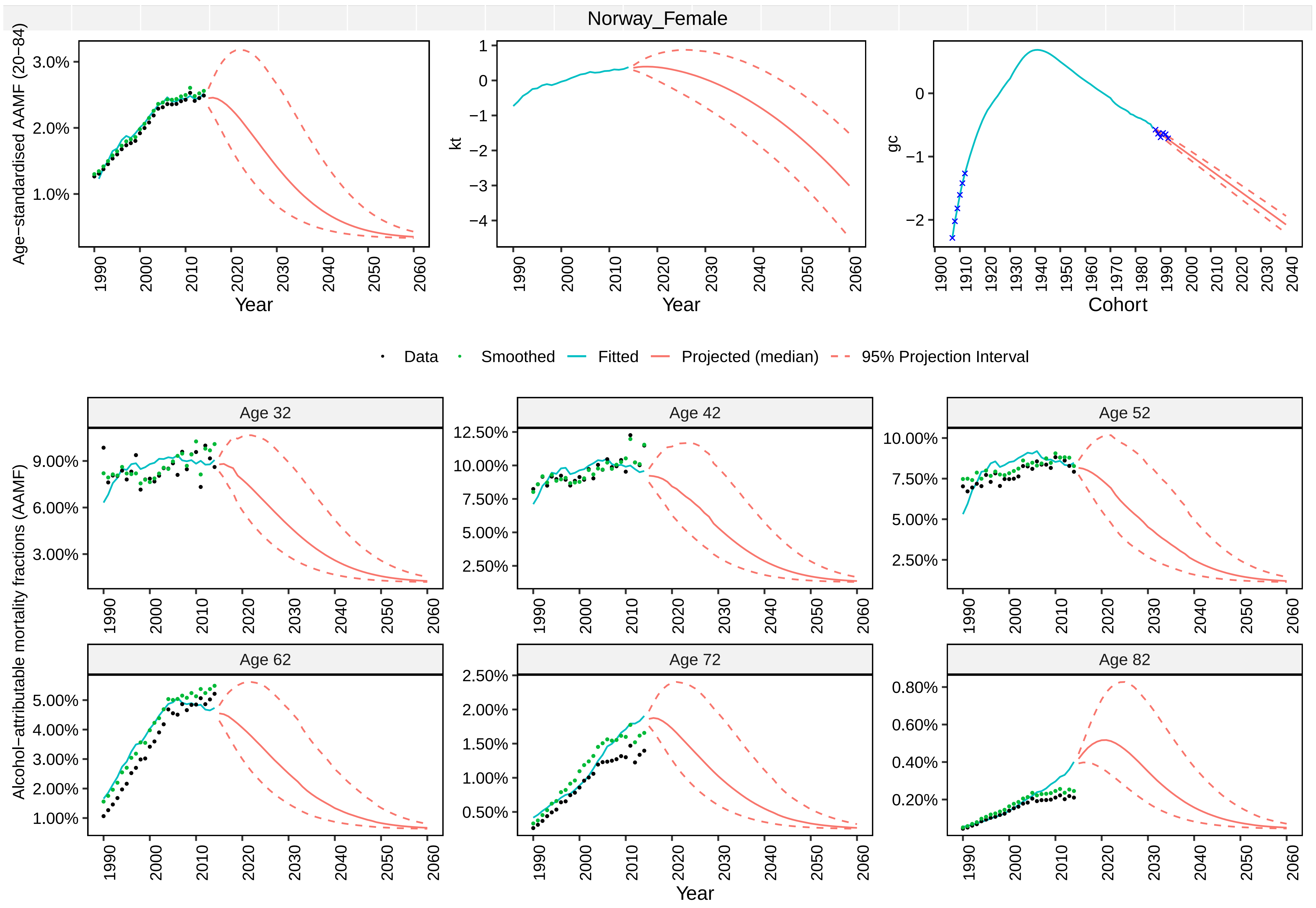
Norway_Female
Age−standardised AAMF (20−84)
3.0%
2.0%
1.0%
Year
kt
gc
Cohort
Data
Smoothed
Fitted
Projected (median)
95% Projection Interval
Age 32
Age 42
Age 52
Age 62
Age 72
Age 82
Alcohol−attributable mortality fractions (AAMF)
Year

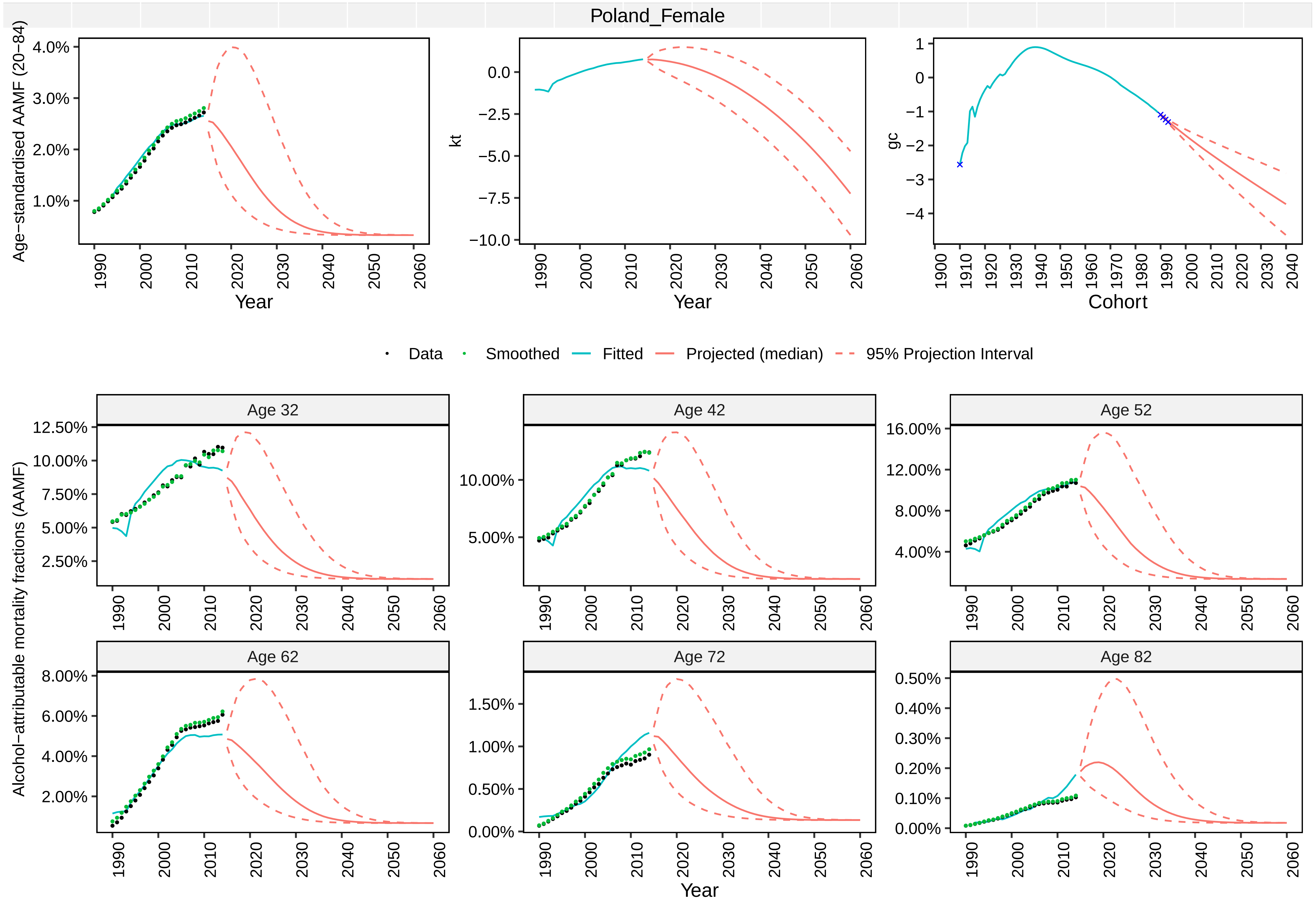

Poland_Female
Age−standardised AAMF (20−84)
4.0%
3.0%
2.0%
1.0%
Year
kt
0.0
−2.5
−5.0
−7.5
−10.0
gc
Cohort
Data
Smoothed
Fitted
Projected (median)
95% Projection Interval
Age 32
Age 42
Age 52
Age 62
Age 72
Age 82
Alcohol−attributable mortality fractions (AAMF)
Year

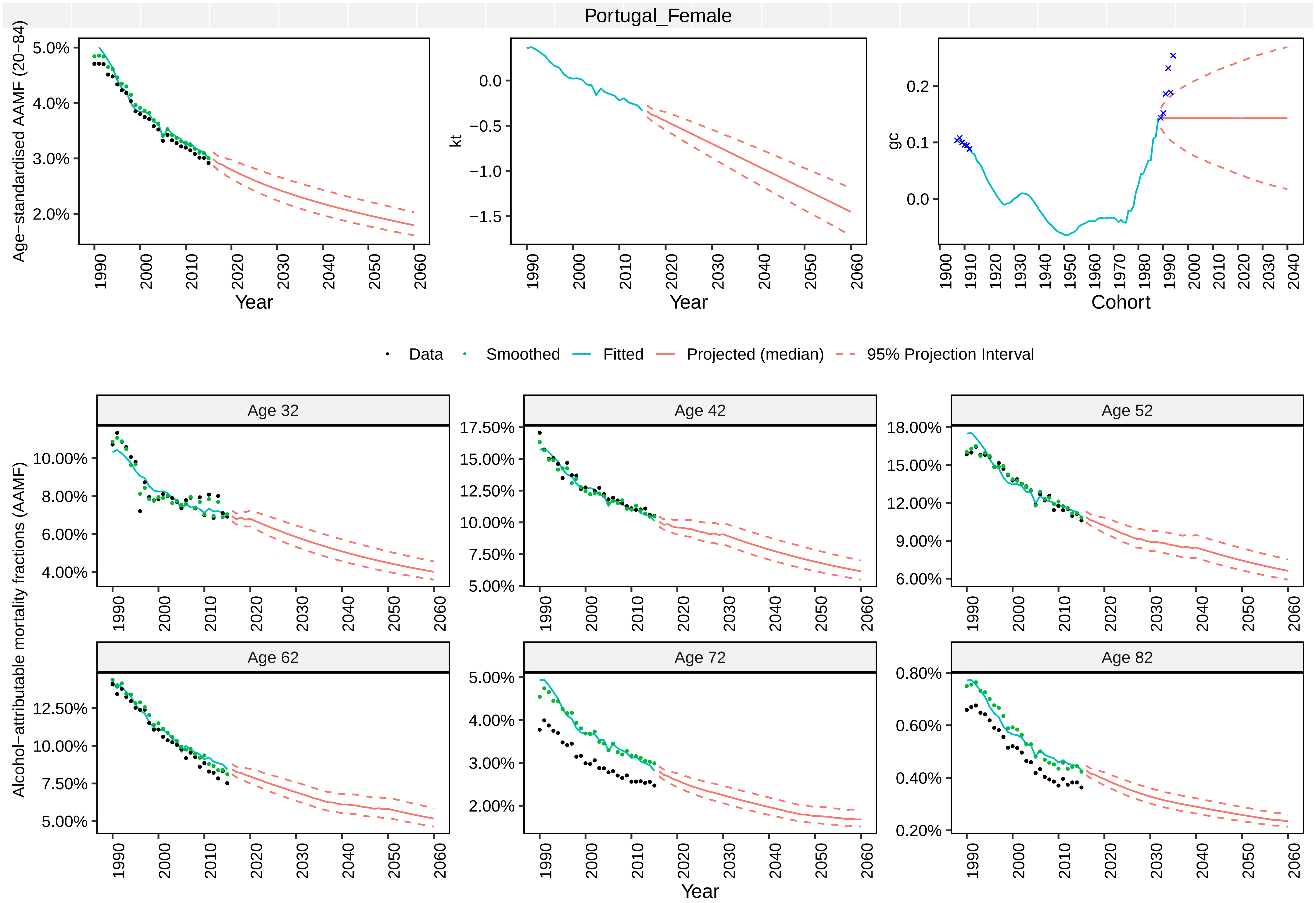

Portugal_Female
Age−standardised AAMF (20−84)
5.0%
4.0%
3.0%
2.0%
Year
kt
0.0
−0.5
−1.0
−1.5
gc
0.2
0.1
0.0
Cohort
1900
1910
1920
1930
1940
1950
1960
1970
1980
1990
2000
2010
2020
2030
2040
2050
2060
Data
Smoothed
Fitted
Projected (median)
95% Projection Interval
Alcohol−attributable mortality fractions (AAMF)
Age 32
10.00%
8.00%
6.00%
4.00%
Age 42
17.50%
15.00%
12.50%
10.00%
7.50%
5.00%
Age 52
18.00%
15.00%
12.00%
9.00%
6.00%
Age 62
12.50%
10.00%
7.50%
5.00%
Age 72
5.00%
4.00%
3.00%
2.00%
Age 82
0.80%
0.60%
0.40%
0.20%
Year

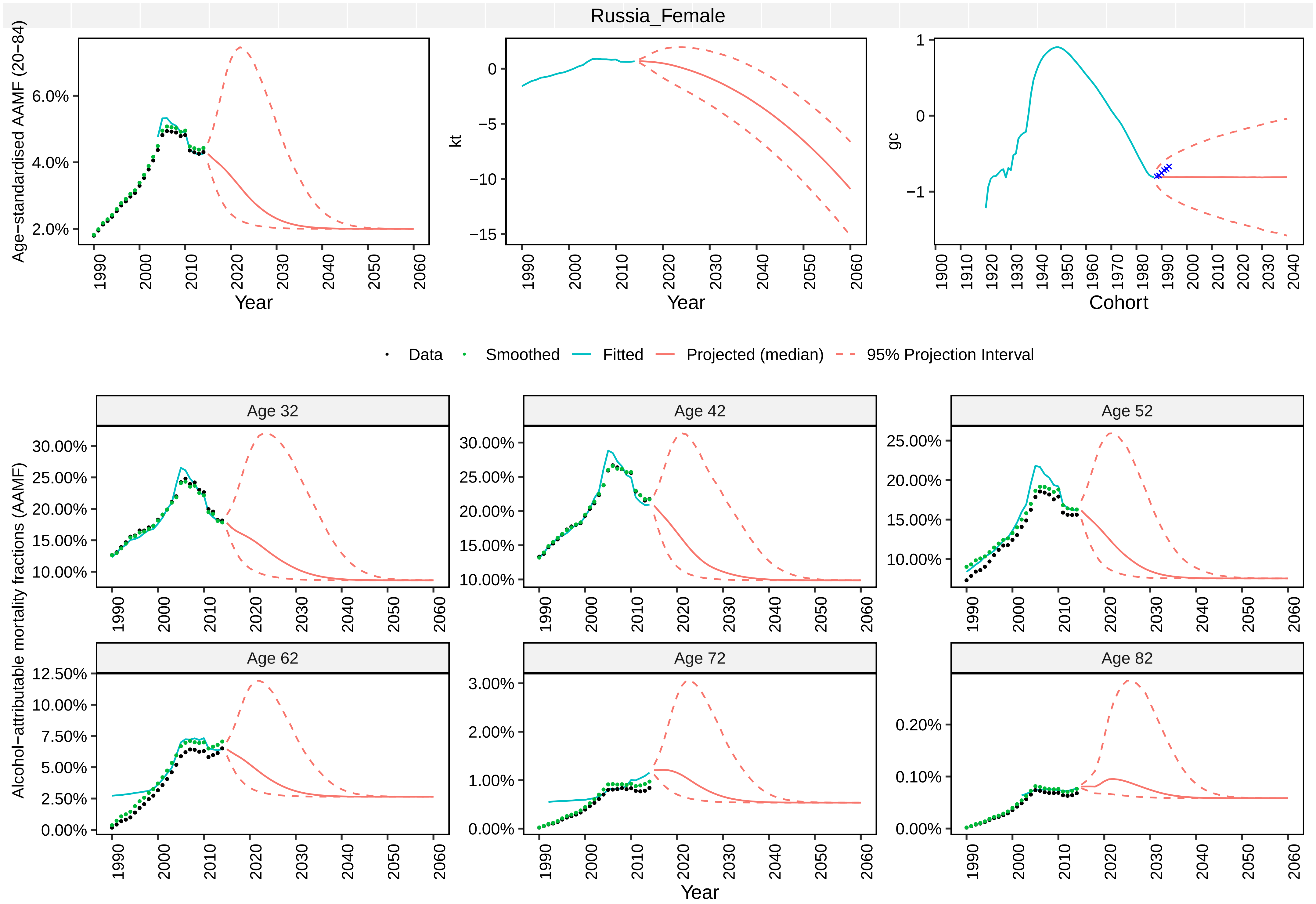

Russia_Female
Age−standardised AAMF (20−84)
6.0%
4.0%
2.0%
Year
kt
0
−5
−10
−15
Year
gc
1
0
−1
Cohort
Data
Smoothed
Fitted
Projected (median)
95% Projection Interval
Alcohol−attributable mortality fractions (AAMF)
Age 32
Age 42
Age 52
Age 62
Age 72
Age 82
Year

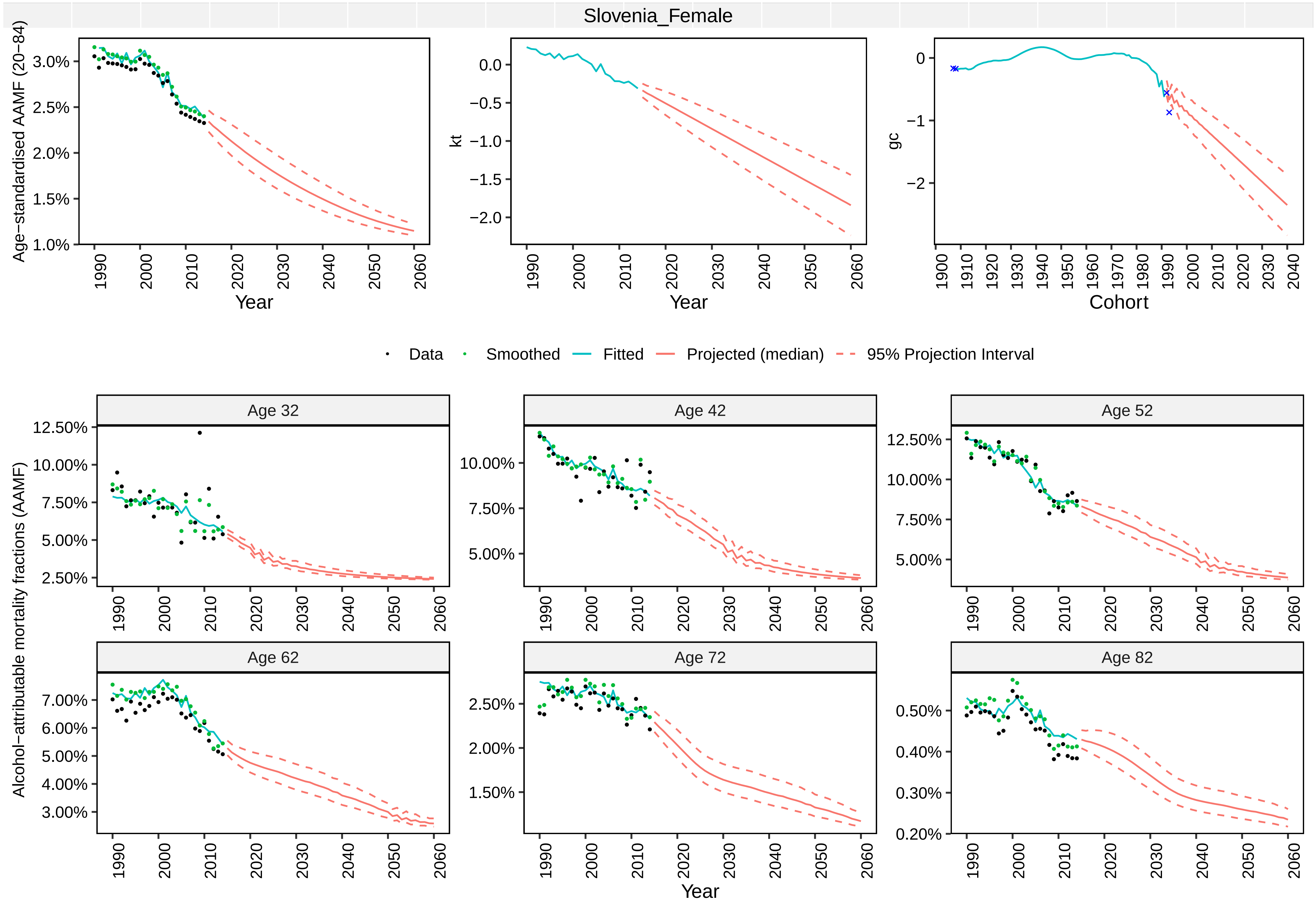
Slovenia_Female
Age−standardised AAMF (20−84)
3.0%
2.5%
2.0%
1.5%
1.0%
Year
kt
0.0
−0.5
−1.0
−1.5
−2.0
gc
0
−1
−2
Cohort
Data
Smoothed
Fitted
Projected (median)
95% Projection Interval
Alcohol−attributable mortality fractions (AAMF)
Age 32
Age 42
Age 52
Age 62
Age 72
Age 82
12.50%
10.00%
7.50%
5.00%
2.50%
7.00%
6.00%
5.00%
4.00%
3.00%
2.50%
2.00%
1.50%
0.50%
0.40%
0.30%
0.20%
1990
2000
2010
2020
2030
2040
2050
2060
Year

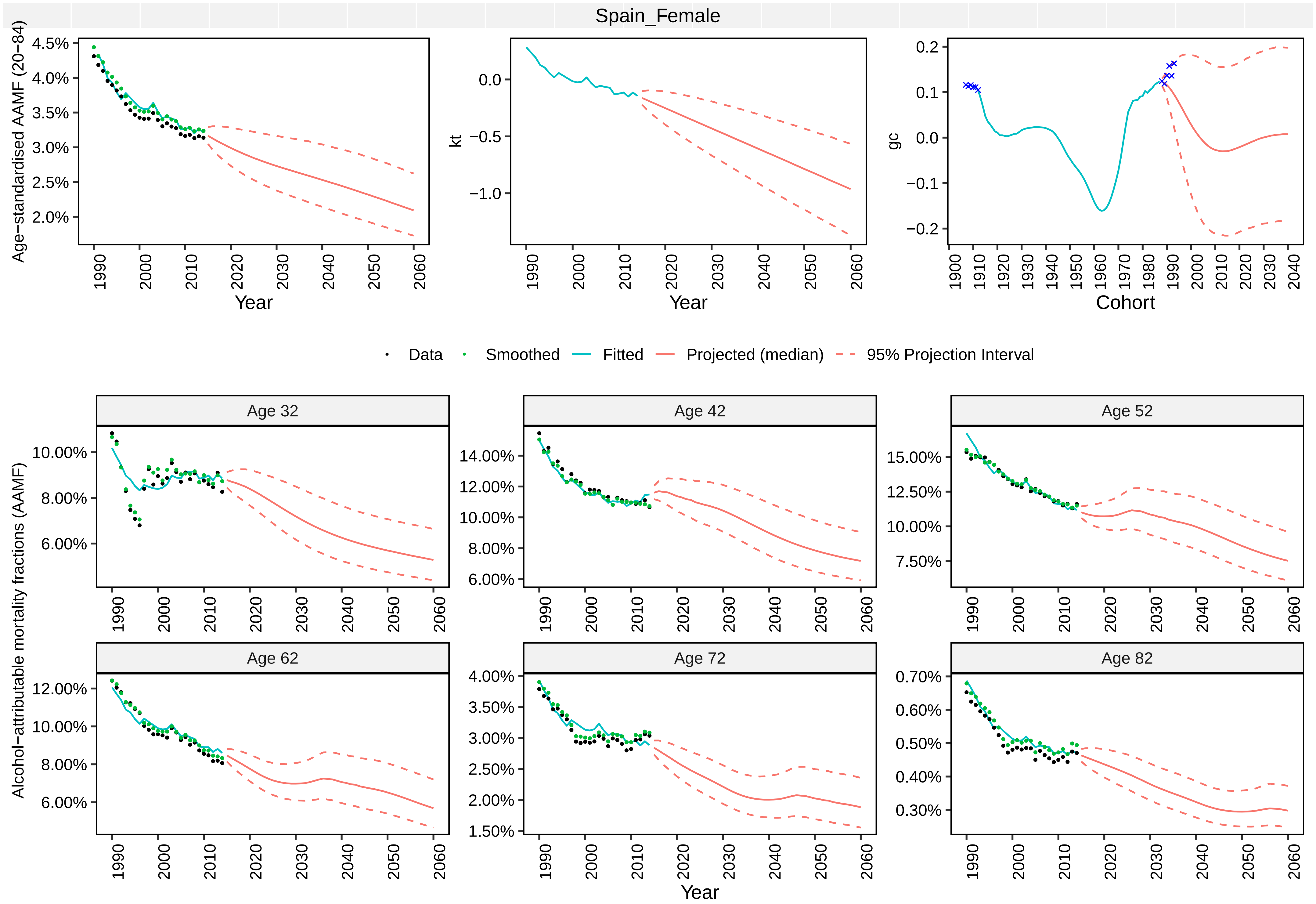
Spain_Female
Age−standardised AAMF (20−84)
4.5%
4.0%
3.5%
3.0%
2.5%
2.0%
Year
kt
0.0
−0.5
−1.0
gc
0.2
0.1
0.0
−0.1
−0.2
Cohort
1990
2000
2010
2020
2030
2040
2050
2060
1900
1910
1920
1930
1940
1950
1960
1970
1980
Data
Smoothed
Fitted
Projected (median)
95% Projection Interval
Age 32
Age 42
Age 52
Age 62
Age 72
Age 82
Alcohol−attributable mortality fractions (AAMF)
10.00%
8.00%
6.00%
14.00%
12.00%
15.00%
12.50%
7.50%
4.00%
3.50%
3.00%
2.50%
2.00%
1.50%
0.70%
0.60%
0.50%
0.40%
0.30%
Year

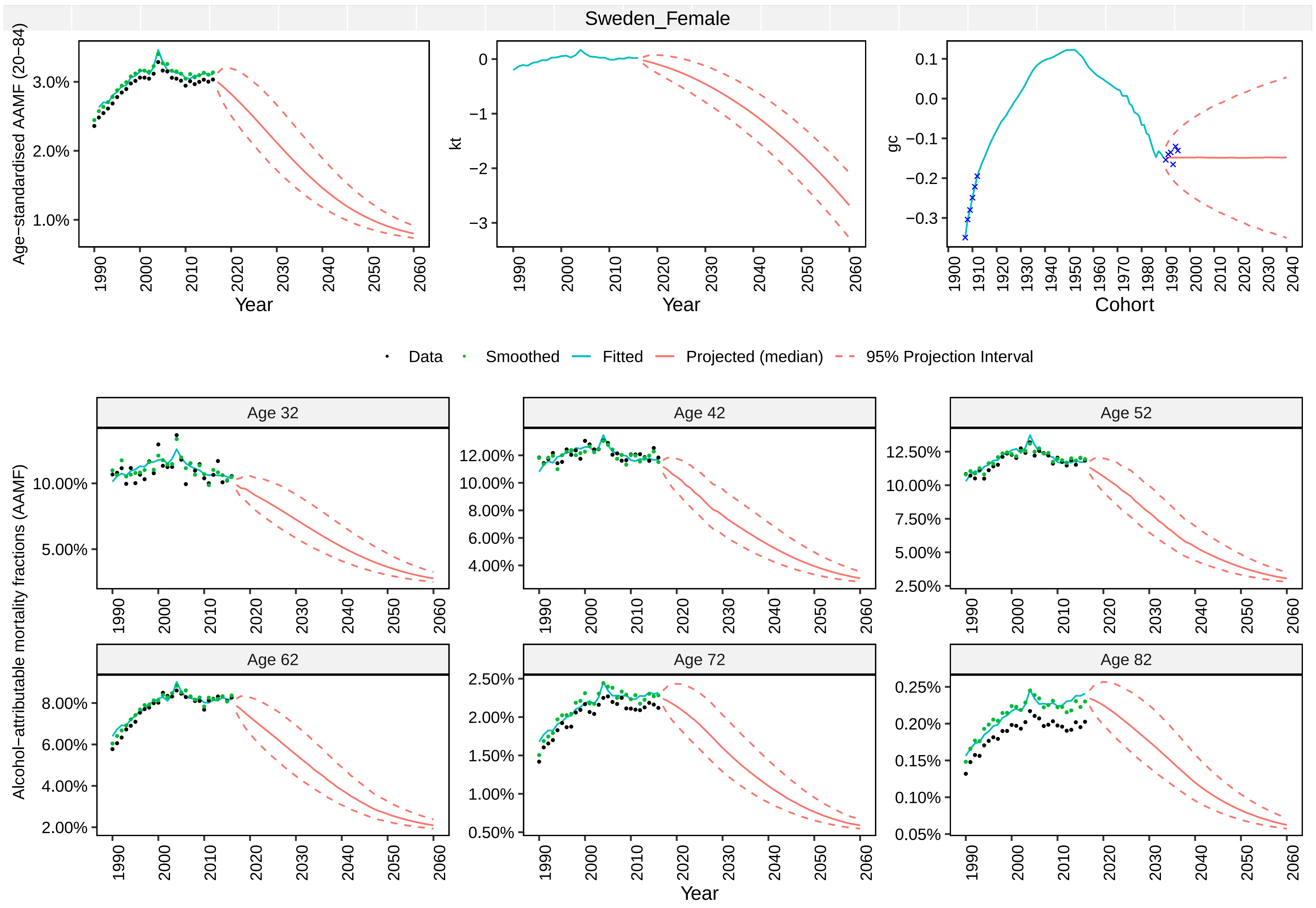
Sweden_Female
Age−standardised AAMF (20−84)
Year
kt
gc
Cohort
Data
Smoothed
Fitted
Projected (median)
95% Projection Interval
Age 32
Age 42
Age 52
Age 62
Age 72
Age 82
Alcohol−attributable mortality fractions (AAMF)
Year

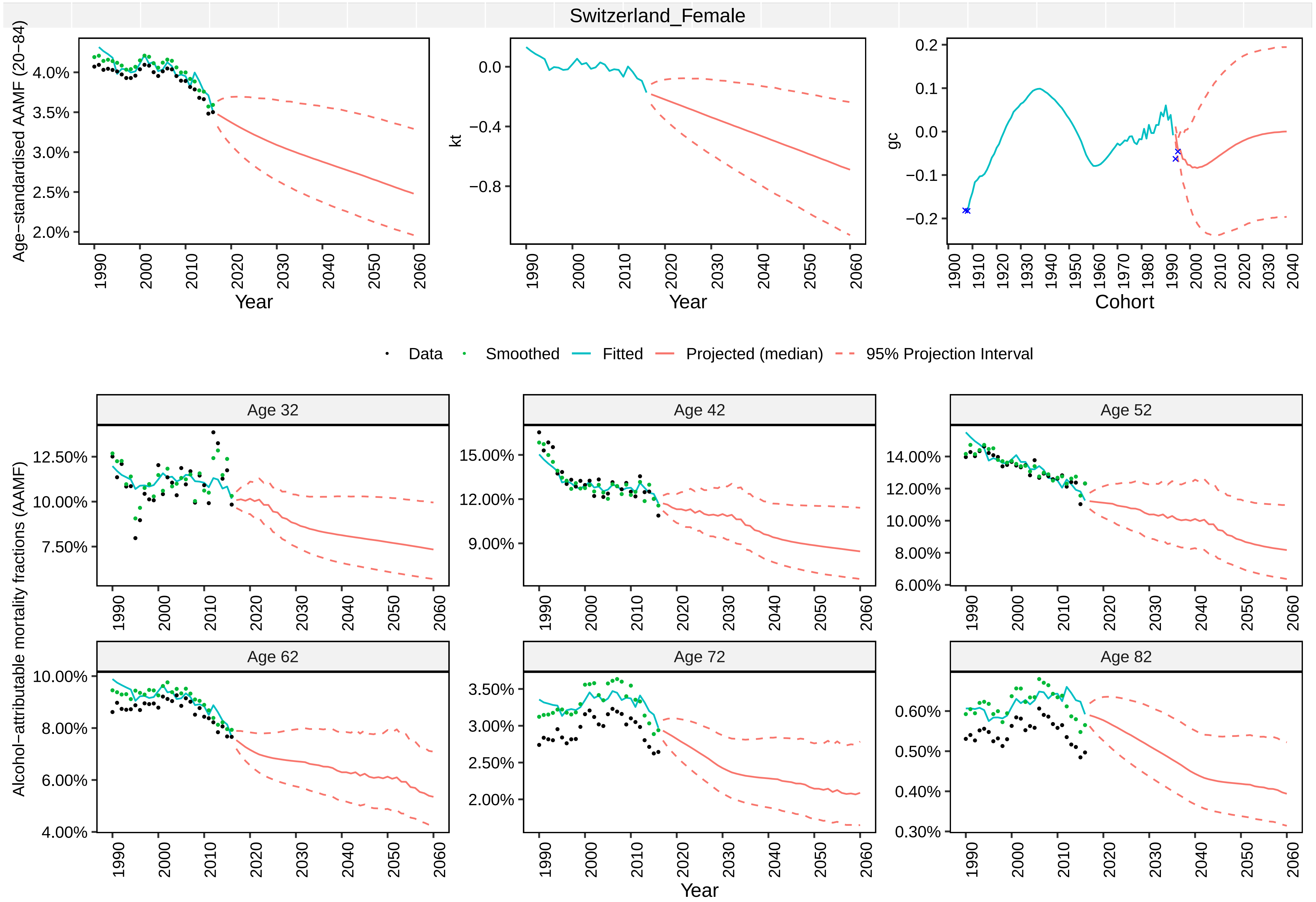
Switzerland_Female
Age−standardised AAMF (20−84)
4.0%
3.5%
3.0%
2.5%
2.0%
1990
2000
2010
2020
2030
2040
2050
2060
Year
kt
0.0
−0.4
−0.8
gc
0.2
0.1
0.0
−0.1
−0.2
1900
1910
1920
1930
1940
1950
1960
1970
1980
1990
2000
2010
2020
2030
2040
Cohort
Data
Smoothed
Fitted
Projected (median)
95% Projection Interval
Age 32
12.50%
10.00%
7.50%
Age 42
15.00%
12.00%
9.00%
Age 52
14.00%
12.00%
10.00%
8.00%
6.00%
Age 62
10.00%
8.00%
6.00%
4.00%
Age 72
3.50%
3.00%
2.50%
2.00%
Age 82
0.60%
0.50%
0.40%
0.30%
Alcohol−attributable mortality fractions (AAMF)
Year

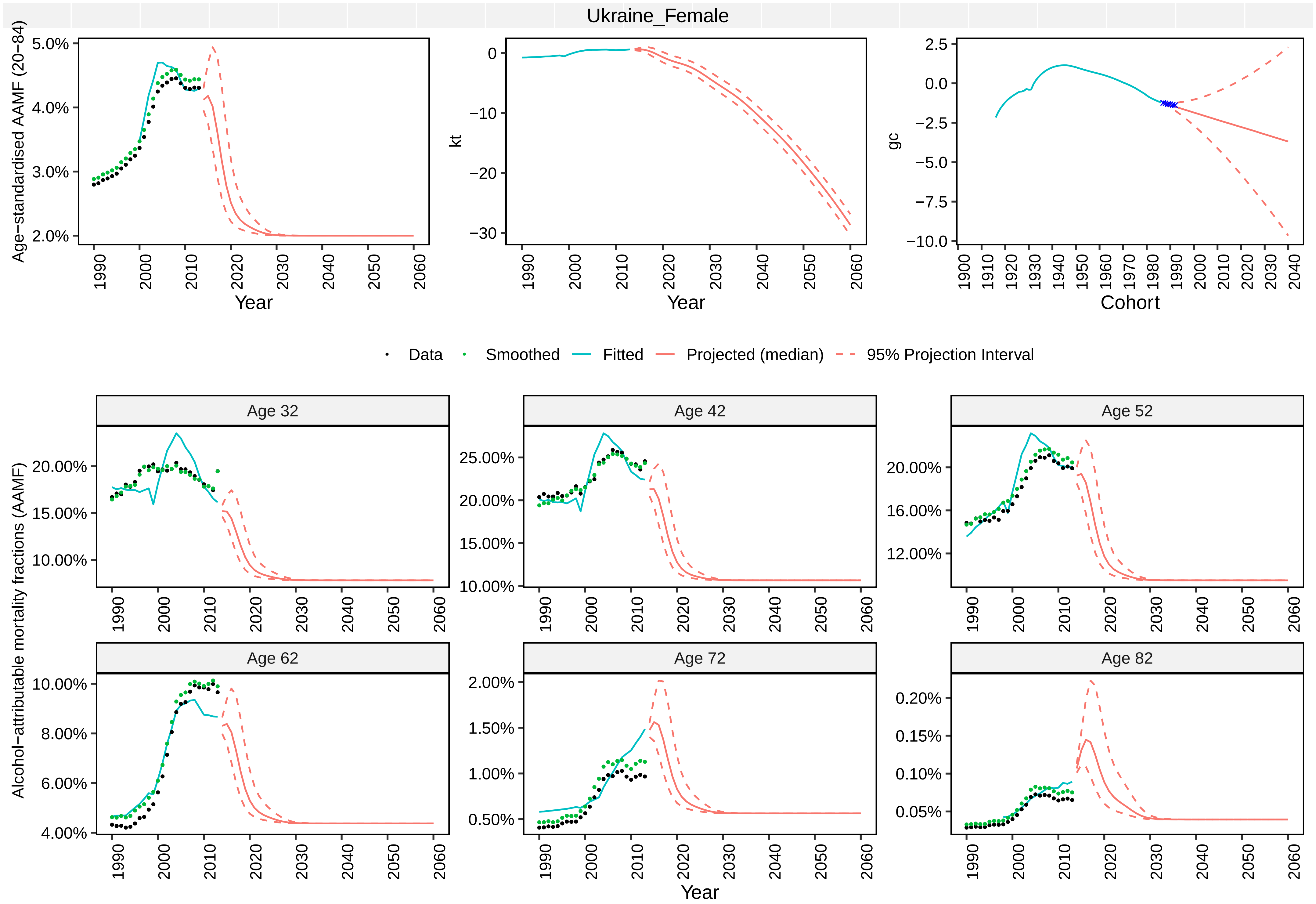

Ukraine_Female
Age−standardised AAMF (20−84)
5.0%
4.0%
3.0%
2.0%
Year
kt
Year
gc
Cohort
Data
Smoothed
Fitted
Projected (median)
95% Projection Interval
Age 32
Age 42
Age 52
Age 62
Age 72
Age 82
Alcohol−attributable mortality fractions (AAMF)
Year

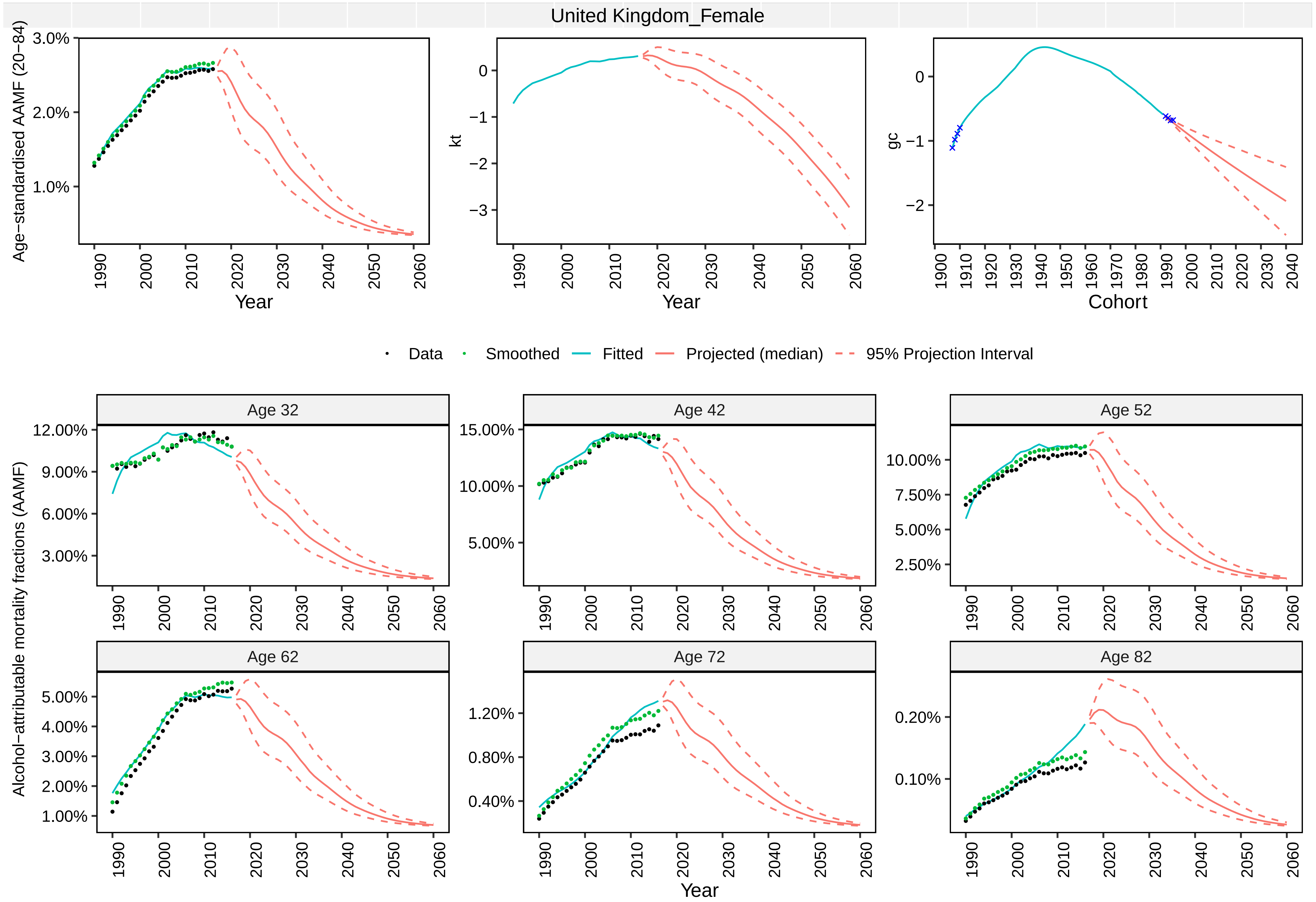

United Kingdom_Female
Age−standardised AAMF (20−84)
Year
kt
gc
Cohort
Data
Smoothed
Fitted
Projected (median)
95% Projection Interval
Age 32
Age 42
Age 52
Age 62
Age 72
Age 82
Alcohol−attributable mortality fractions (AAMF)
Year
